# A systematic review of microRNA expression profiling studies in human gastric cancer

**DOI:** 10.1002/cam4.246

**Published:** 2014-06-05

**Authors:** Sirjana Shrestha, Sheng-Da Hsu, Wei-Yun Huang, Hsi-Yuan Huang, WenLiang Chen, Shun-Long Weng, Hsien-Da Huang

**Affiliations:** 1 Department of Biological Science and Technology, National Chiao Tung UniversityHsinchu, 300, Taiwan; 2 Institute of Bioinformatics and Systems Biology, National Chiao Tung UniversityHsinchu, 300, Taiwan; 3 Center for Bioinformatics Research, National Chiao Tung UniversityHsinchu, 300, Taiwan; 4 Department of Obstetrics and Gynecology, Hsinchu Mackay Memorial HospitalHsinchu, 300, Taiwan; 5 Mackay Medicine, Nursing and Management CollegeTaipei, 112, Taiwan; 6 Department of Medicine, Mackay Medical CollegeNew Taipei City, 252, Taiwan; 7 Department of Biomedical Science and Environmental Biology, Kaohsiung Medical UniversityKaohsiung, 807, Taiwan

**Keywords:** Biomarker, gastric cancer, microRNA, miRNA target genes

## Abstract

Gastric cancer (GC) is the second leading cause of global cancer mortality. Most GC patients are diagnosed with advanced-stage disease and show extremely poor prognosis. All of the GC research has a common interest to search for the specific and sensitive biomarkers for early diagnosis of GC. Number of microRNAs play important role in GC. We carried out a systematic review of published miRNA profiling studies that compared the miRNA expression profiles between GC tissues and paired noncancerous gastric tissue. A vote-counting strategy was followed with the collection of information like total number of studies reporting differential expression of miRNA, total number of tissue samples used in the studies, direction of differential expression and fold change. A total of 352 differentially expressed microRNAs were reported in the 14 microRNA expression profiling studies that compared GC tissues with normal tissues with 120 microRNAs reported at least in two studies. In the group of consistently reported microRNAs, miR-21 was reported upregulated in 10 studies followed by miR-25, miR-92, and miR-223 upregulated in eight studies. MiR-375 and miR-148a were found downregulated in six and five studies, respectively, followed by miR-638 in four studies. MiR-107 and miR-103 were reported in nine and eight studies, respectively, but their expression were inconsistent. From this study, the most consistently reported upregulated microRNA was found to be miR-21. This systematic review study of human GC microRNA expression profiling studies would provide information on microRNAs with potential role as the biomarkers in gastric cancer.

## Introduction

Gastric cancer (GC) is the second leading cause of cancer-related death worldwide. About one million new cases per year of GC were estimated to have occurred 1. GC is diagnosed at advanced stage accompanied by extensive invasion and lymphatic metastasis 2. Thus, early detection of GC is a key measure to reduce the mortality and improve the prognosis of gastric cancer. Recent studies on diagnostic techniques and peri-operative management have increased the early detection of GC and decreased the mortality 3,4. Therefore, it is very important to increase the sensitivity and specificity of diagnostic markers and/or methods for the treatment and prevention of GC 5.

MicroRNAs are a class of ∼22 nucleotides noncoding RNAs that regulate the expression of target genes by interacting with complementary sites in the 3′ untranslated region of the target mRNAs 6. Studies have shown that miRNAs have been involved in the regulation of different biological processes, including apoptosis, proliferation, metabolism, cellular differentiation, embryogenesis, gene regulation 7,8. Recently microRNA expression profiling has been extensively used in order to screen the expression of the large number of microRNAs through extensive sample collections. Several microRNA expression profiling studies in cell lines, tissue samples, serum have revealed the number of microRNAs as the biomarkers in GC 9–11. These studies provide hundreds of differentially expressed miRNAs, and finally only a small number of them may have clinical use and act as diagnostic and prognostic biomarkers. Different profiling studies show inconsistency in the identified differentially expressed miRNAs. A logical method to identify the most consistently reported differentially expressed miRNAs is to search for the intersections of miRNAs identified in multiple independent studies. Griffith 12 and Chan 13 vote-counting strategy have cope with the challenges to collect and combine the results of those miRNAs expression profiling studies with different profiling platform, different methods to ascertain differential expression like normalization and significance level.

We carried out a systematic review from the published microRNAs expression profiling studies that compared the miRNA expression profiles in GC tissues with those in noncancerous gastric tissues. A vote-counting strategy was used which considered the total number of studies that reported differential expression, total number of tissue samples used in studies and the average fold change. We also presented the consistently reported differential miRNAs and the rank of the differentially expressed upregulated and downregulated miRNAs.

## Materials and Methods

### Literature survey

In order to find all relevant literatures, we used PubMed search engine for the GC microRNA expression profiling studies between the periods February 2006 and December 2012 14–27. Search term “miR and gastric cancer” was used. Papers with miRNA expression profiling studies that used tissue samples obtained from surgically resected GC tumors and corresponding noncancerous gastric tissues were selected.

### Study selection criteria

Selected study should meet the criteria: (1) they were miRNA expression profiling studies in GC patients, (2) they used GC and neighboring noncancerous gastric tissues for comparison, (3) use of microarray methods, (4) had information on cut off criteria of differentially expressed miRNAs, and (5) had reported total number of samples used in study. Thus, due to this inclusion criteria miRNA profiling studies using different cell lines, serum samples of GC patients, plasma samples, or using different miRNA technologies (RNA-seq and qPCR) and review articles were excluded.

### Data collection

From each selected paper, following information were retrieved: author, journal and year of publication, location of study, period of sample collection (if available), selection and characteristics of recruited GC patient, total samples used for the study, platform used for miRNA expression profiling, cut-off criteria of statistically differentially expressed miRNAs, total number of differentially expressed miRNAs, list of upregulated and downregulated miRNAs and their fold change (if available).

### Ranking

Each of the published miRNA expression profiling studies that compare miRNA expression between the GC and neighboring noncancerous gastric tissues provided a list of differentially expressed miRNAs. Then for systematic review the vote-counting strategy based method of ranking potential molecular markers as given by Griffith 12 and Chan 13 was used. MiRNAs were ranked on the basis of criteria as: (1) number of the studies that consistently reported the miRNA as differentially expressed, (2) the consistent direction of change of differentially expressed microRNA, and (3) total number of samples for comparison in agreement.

### Identify the experimentally validated microRNA target genes

To explore the target genes of miRNAs related to GC miRTarBase (mirtarbase.mbc.nctu.edu.tw), was used which is the recently established database with largest collection of more than 34,000 manually curated miRNA-target interactions, all of which are experimentally validated by luciferase reporter assay, western blot, or microarray experiments with overexpression or knockdown of miRNAs 28.

### Enrichment analysis

Enrichment analyses for gene ontology (GO) 29 terms and Kyoto encyclopedia of genes and genomes (KEGG) 30 pathways were carried out with Database for Annotation, Visualization, and Integrated Discovery (DAVID) 31. DAVID is a web-accessible program that integrates functional genomics annotations with intuitive graphical summaries. List of gene or protein identifiers are rapidly annotated and summarized according to shared categorical data for Gene Ontology, protein domain, and biochemical pathway membership. For this, we listed all the target genes of downregulated microRNAs. The top 20 GO terms and KEGG pathways showing association with target genes were listed with GO terms, KEGG pathway, number of genes in the term, number of genes in the pathways, and *P*-value.

## Results

### Included independent studies

**Figure 1:**
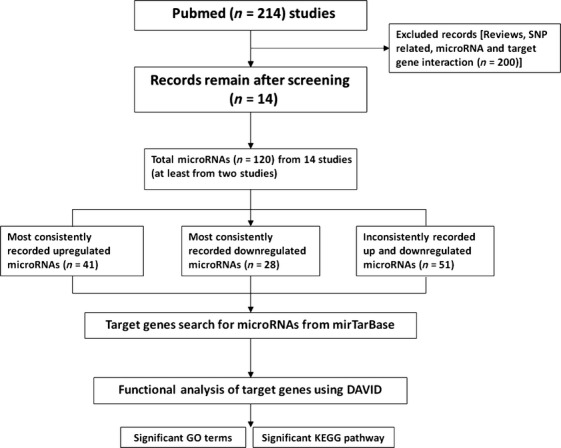
System flowchart for systematic review.

In total, 214 studies were recorded using PubMed. According to the inclusion and exclusion criteria, only 14 independent studies were included in our analysis. The system work flow that was used in our analysis was as shown in Figure1. The information of the selected studies was tabulated in Table1 in the descending order of their year of publication. These studies show the different platforms as well as various statistical and biocomputational analyses had been used for the microRNA expression profiling. The number of differentially expressed microRNAs ranges from 9 to 326. Two studies Carvalho 14, Oh 18 provided the top five and top 40 differentially expressed microRNAs, respectively, one study Tsukamoto 21 provided the differentially expressed microRNAs with the raw signal over 500 and one study Yao 23 provided the differentially expressed microRNAs above twofold changes. Nine of the 14 eligible studies provided fold change (FC) information of differentially expressed miRNAs.

**Table 1:** Characteristics of miRNA datasets in human gastric cancer.

Dataset	Year	Region	Tumor type	No. of samples (cancer/normal)	Platform (manufacturer)	Total miRNA	Differentially expressed miRNAs			
							Criteria	Up	Down	Total
Carvalho 14	2012	Netherlands	GCI	47 (37/10)	miRNAChip_human_v2 (National DNA-Microarray Facility, University of Aveiro, Portugal)	703	*P* < 0.05, FDR < +0.05	5[Table-fn tf1-1]	5[Table-fn tf1-1]	70
Kim 15	2011	Korea	GCI, GCD	124 (90/34)	LMT miRNA microarray (Agilent technologies)	1667	*P* < 0.005	62	63	125
Li 16	2011	China	GCI	12 (6/6)	miRCURY Array LNA microRNA Chip (v.14.0) (Exiqon)	904	*P* < 0.01 FC > 2	40	36	76
Li 17	2011	China	NR	20 (10/10)	TaqMan Human miRNA Array v1.0 (Applied Biosystems)	365	*P* < 0.05	16	6	22
Oh 18	2011	Singapore	GCI, GCD	80 (40/40)	Agilent Human miRNA Microarrays (V2, Agilent)	723	FDR < 0.01	40[Table-fn tf1-1]	40[Table-fn tf1-1]	146
Tchernitsa 19	2010	Germany	GCI	12 (6/6)	NCode TM MultiSpecies miRNA Microarray V1 (Invitrogen)	373	Significance of class comparison = 0.05	20	2	22
Ding 20	2010	China	GCI, GCD	12 (6/6)	*μ*Paraflo microfluidic chip (LC Sciences)	NR	*P* < 0.05	8	7	15
Tsukamoto 21	2010	Japan	GCI, GCD	27 (22/5)	G4470A Human MiRNA Microarray (Agilent technologies)	470	*P *< 0.05	33[Table-fn tf1-2]	6[Table-fn tf1-2]	102
Ueda 22	2010	Japan	GCI, GCD	353 (184/169)	microRNA microarray chip (OSU_CCC version 3.0, ArrayExpress)	326	*P* < 0.01	22	13	35
Yao 23	2009	China	NR	6 (3/3)	miRCURY LNA microarray Array(v.11.0) (Exiqon)	847	FC > 2	59[Table-fn tf1-3]	46[Table-fn tf1-3]	326
Luo 24	2009	China	NR	27 (24/3)	NR	328	*P* < 0.05	7	19	26
Liu 25	2009	China	NR	8 (4/4)	microRNA Microarray (Packard Biochip Technologies ScanArray Express microarray)	243	*P* < 0.05	4	5	9
Petrocca 26	2008	Italy	GCI	40 (20/20)	second generation miRNA microarray chips (V2)(Amersham BioScience Codelink)	250	Significance analysis of microarray (SAM)	14	5	19
Volinia 27	2006	USA	NR	41 (20/21)	miRNA microarray (Amersham BioScience Codelink)	190	FDR = 0.06	22	6	28

Only top most miRNAs selected.

miRNAs above with raw signal above 500 selected 21_._

miRNAs over twofold change provided (GCI, gastric cancer intestinal; GCD, gastric cancer diffuse, NR, not reported).

### Differentially expressed microRNAs

A total of 352 differentially expressed miRNAs were reported from 14 microRNA expression profiling studies that compared GC tissue with corresponding noncancerous gastric tissue, with 120 microRNAs reported in at least two studies.

### Consistently and inconsistently reported microRNAs

Among 352 microRNAs, 120 miRNAs (34.18%) were reported in at least two studies. Among 120 differentially expressed miRNAs, 69 miRNAs (57.5%) were with consistent direction of expression of which 41 were reported to be upregulated (Table2) and 28 downregulated (Table3). Fifty-one miRNAs (42.5%) were reported to be with inconsistent direction of expression (Table S1).

**Table 2:** Consistently reported upregulated miRNAs in profiling studies (gastric cancer tissue vs. normal).

miRNA	Reference	No. of tissue sample (cancer/normal)	miRNA family	Fold change	Median fold change
miR-21	10 15–22,26,27	721 (404/317)	mir-21	1.49–10.44	4.05
miR-25	8 15,17,19–22,26,27	629 (358/271)	mir-25	1.26–5.57	2.55
miR-92	8 15–17,19,21,22,26,27	629 (358/271)	mir-25	1.39–5.24	2.80
miR-223	8 15–18,21,23,26,27	350 (211/139)	mir-223	2.13–4.90	3.13
miR-106b	7 15,19–23,26	574 (331/243)	mir-17	1.60–4.30	2.00
miR-106a	7 15,16,20–23,26	574 (331/243)	mir-17	1.52–9.02	2.80
miR-18a	6 15,16,18,21–23	602 (345/257)	mir-17	1.70–10.66	2.27
miR-93	6 15,16,19,21–23	568 (328/240)	mir-17	1.49–8.27	2.40
miR-17	6 15,16,21–23,26	562 (325/237)	mir-17	1.61–9.01	3.08
miR-23a	5 17,18,21,25,27	176 (96/80)	mir-23	—	2.89
miR-191	5 15,17,22,25,27	546 (308/238)	mir-191	1.27–1.30	1.28
miR-19a	5 15,16,18,21,22	596 (342/254)	mir-19	1.50–7.80	3.99
miR-20a	5 15,16,18,21,22	596 (342/254)	mir-17	1.35–17.74	4.71
miR-27a	5 16,18,19,21,25	139 (78/61)	mir-27a	2.96–4.72	3.32
miR-214	5 15,17–19,27	165 (82/83)	mir-214	2.11–2.85	2.48
miR-100	4 17,23,25,27	107 (53/54)	mir-10	2.10–2.17	2.13
miR-20b	4 15,20–22	516 (302/214)	mir-17	1.28–6.84	1.84
miR-425-5p	3 15,21,22	504 (296/208)	mir-425	1.35–5.27	2.20
miR-7	3 19,21,27	80 (48/32)	mir-7	3.11–4.68	3.89
miR-215	3 18,21,27	148 (82/66)	mir-192	–	3.78
miR-135b	3 15,18,22	557 (314/243)	mir-135	1.59–1.60	1.59
miR-224	3 15,18,22	557 (314/243)	mir-224	2.20–3.85	3.02
miR-192	3 18,21,27	148 (82/66)	mir-192	–	3.74
miR-221	3 17,25,27	101 (50/51)	mir-221	–	2.40
miR-18b	3 15,16,18	216 (136/80)	mir-17	2.17–5.01	3.59
miR-200b	3 16,18,21	119 (68/51)	mir-8	2.40–2.57	2.48
miR-194	2 20,21	39 (28/11)	mir-194	2.13–5.16	3.64
miR-99b	2 23,27	47 (23/24)	mir-10	–	2.18
miR-10a	2 21,23	33 (25/8)	mir-10	2.49–4.77	3.63
miR-15a	2 18,21	107 (62/45)	mir-15	–	2.32
miR-199-5p	2 18,23	86 (43/43)	mir-199	–	2.98
miR-301	2 15,18	204 (130/74)	mir-130	–	1.82
miR-519d	2 15,23	130 (93/37)	mir-515	1.59–2.57	2.08
let-7i	2 15,18	204 (130/74)	let-7	–	1.20
miR-181d	2 15,22	477 (274/203)	mir-181	1.16–2.30	1.73
miR-185	2 15,23	130 (93/37)	mir-185	1.32–2.20	1.76
miR-181a	2 15,22	477 (274/203)	mir-181	1.20–2.20	1.70
miR-1259	2 16,23	18 (9/9)	–	3.00–19.89	11.44
miR-335	2 16,20	24 (12/12)	mir-335	2.01–5.81	3.91
miR-424	2 15,16	136 (96/40)	mir-322	1.52–3.37	2.44
miR-542-3p	2 16,23	18 (9/9)	mir-542	2.65–3.03	2.84

**Table 3:** Consistently reported downregulated miRNAs in profiling studies (gastric cancer tissue vs. normal).

miRNA	Reference	No. of tissue sample (cancer/normal)	miRNA family	Fold change	Median fold change
miR-375	6 15,16,18,20–22	608 (348/260)	mir-375	0.15–0.37	0.27
miR-148a	5 15,18,19,21,22	596 (342/254)	mir-148	0.19–0.47	0.23
miR-30d	5 15,16,18,20,22	581 (326/255)	mir-30	0.50–0.78	0.69
miR-638	4 18,20,21,23	125 (71/54)	mir-638	0.32–0.57	0.46
miR-29c	4 15,16,21,22	516 (302/214)	mir-29	0.16–0.70	0.40
miR-155	4 15,16,23,24	169 (123/46)	mir-155	0.36–0.66	0.42
miR-378	3 16,18,23	98 (49/49)	mir-378	0.25–0.29	0.27
miR-152	3 15,16,22	489 (280/209)	mir-148	0.44–0.78	0.70
miR-30c	3 15,20,22	489 (280/209)	mir-30	0.67–0.75	0.70
miR-218	3 22,26,27	434 (224/210)	mir-218	–	0.60
miR-133b	2 15,23	130 (93/37)	mir-133	0.07–0.32	0.19
miR-150	2 14,23	130 (93/37)	mir-150	0.46–0.49	0.47
miR-564	2 15,18	204 (130/74)	mir-564	–	0.72
miR-489	2 15,23	130 (93/37)	mir-489	0.41–0.71	0.56
miR-136	2 26,27	81 (40/41)	mir-136	–	0.60
miR-197	2 17,24	47 (34/13)	mir-197	–	–
miR-923	2 18,20	92 (46/46)	–	–	0.46
miR-490	2 23,24	33 (27/6)	mir-490	–	0.35
miR-146a	2 15,19	136 (96/40)	mir-146	0.24–0.39	0.31
miR-188	2 18,24	107 (64/43)	mir-188	–	–
miR-34b	2 15,23	130 (93/37)	mir-34	0.43–0.64	0.53
miR-370	2 18,24	107 (64/43)	mir-370	–	–
miR-139	2 16,18	92 (46/46)	mir-139	–	0.28
miR-513a-5p	2 16,18	92 (46/46)	mir-506	–	0.30
miR-494	2 16,18	92 (46/46)	mir-154	–	0.37
miR-320c	2 16,24	18 (9/9)	mir-320	0.42–0.47	0.44
miR-433	2 16,24	39 (30/9)	mir-433	–	0.48
miR-101	2 14,16	59 (43/16)	mir-101	0.22–0.49	0.35

In the group of consistently reported upregulated miRNAs, miR-21 was reported in 10 studies followed by three miRNAs, miR-25, miR-92 and miR-223 in eight studies. In the consistently reported downregulated miRNAs, miR-375 was reported in six studies followed by two miRNAs, miR-148a, and miR-30d in five studies. However, in the group of inconsistently reported miRNAs, miR-107 was reported in nine studies with upregulation in eight studies and downregulation in one study followed by miR-103 in eight studies with seven and one study showing up- and downregulation, respectively.

### Target genes of differentially expressed microRNAs

After we identified the total microRNAs that were downregulated, upregulated, and both down and upregulated in gastric cancer, we used the miRTarBase to search all of the target genes for the set of miRNAs. We identified 362 and 489 nonredundant target genes for the set of miRNAs downregulated and upregulated, respectively, in GC (Tables S2 and S3). MicroRNAs can act as both tumor suppressor and oncogenes. A total of 701 nonredundant target genes were identified for the set of miRNAs that were both upregulated and downregulated in GC (Table S4). Downregulated microRNAs play important role as tumor suppressors, so we focused on the set of downregulated microRNAs target genes.

### Enrichment analysis result

**Figure 2:**
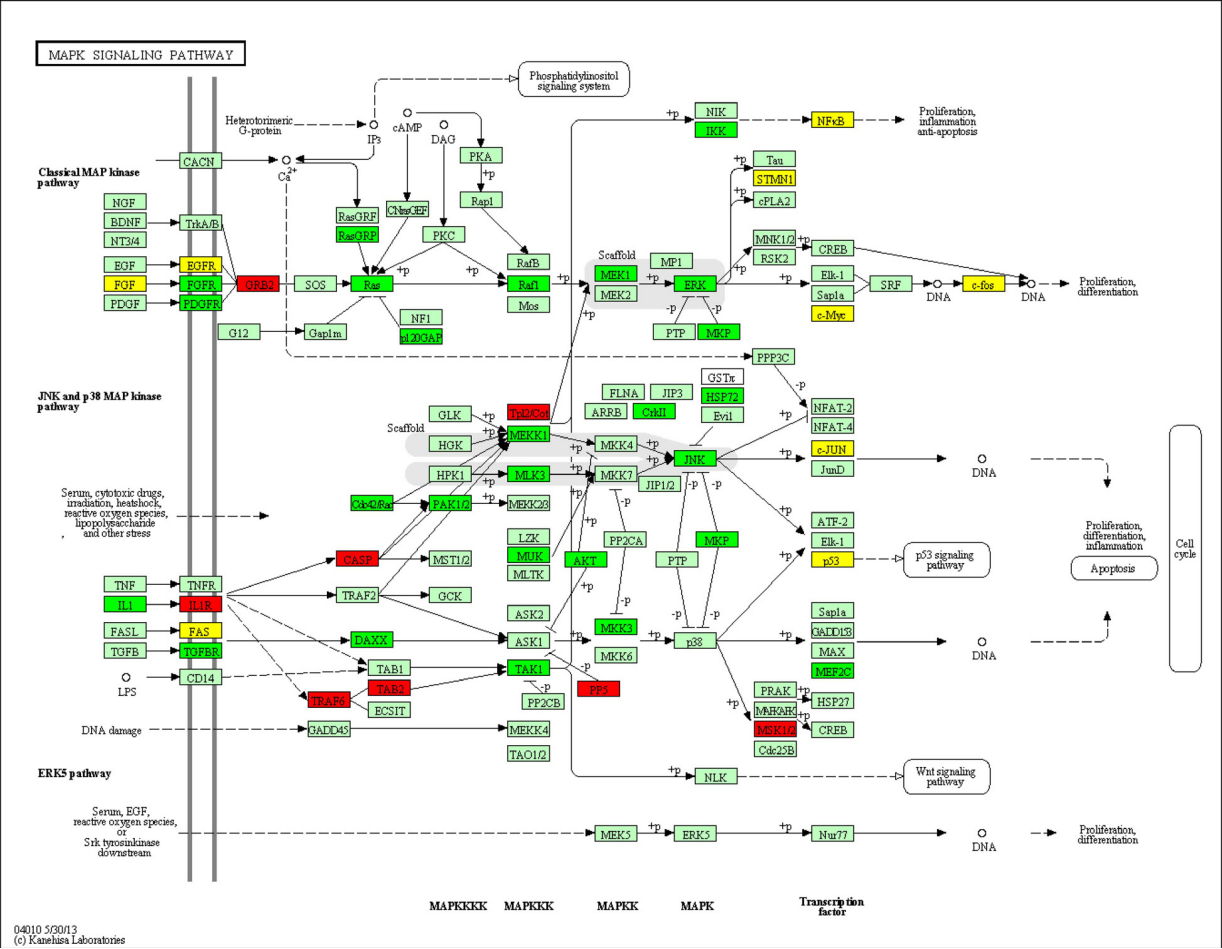
MAPK signaling pathway showing the target genes of microRNAs.

We used Database for Annotation, Visualization, and Integrated Discovery (DAVID) to find the molecular networks of downregulated microRNAs target genes. The top 20 GO terms and KEGG pathways showing significant association with target genes were listed with GO terms, KEGG pathway, number of genes in the terms, number of genes in the pathways, and *P*-value (Tables S5 and S6). The significant GO terms were related to regulation of cell proliferation, regulation of apoptosis, regulation of cell cycle (Table S5 and Fig. S1), and KEGG pathways were related to various types of cancer, focal adhesion, adherens junction, signaling pathways such as MAPK, toll-like receptor and p53 (Table S6 and Fig. S2). One of the signaling pathways, MAPK signaling pathway, plays important role in gastric cancer. Figure2 showed that genes like TRAF6, GRB2, and IL1R involved in MAPK signaling pathway were targeted by downregulated miRNAs marked in red boxes. Green boxes represent the genes targeted by upregulated miRNAs and yellow boxes indicate the genes targeted by both upregulated and downregulated miRNAs. We used KEGG API to produce color-coded pathways shown in Figure2.

## Discussion

As different microRNA expression profiling studies use different platforms and different processing methods, there is the lack of agreement among these studies. Vote counting is the most intuitive and simplest method for combining results from different studies to discover the patterns among those results. Although vote-counting method is statistically inefficient, but it is suitable for dealing with the issue that only the list of differentially expressed genes is available. Here, a possible solution for this problem is to determine the overlap among many studies using different platforms and observe which miRNAs are consistently reported as differentially expressed ones 12,13.

This type of systematic review had been done previously to determine differentially expressed genes in thyroid 12 and colorectal cancer 13 at gene level. Ma 32 carried out the systematic review in exploring candidate miRNA biomarkers in human colorectal cancer. Guan 33 carried out the meta-analysis of human lung cancer microRNA expression profiling studies comparing cancer tissues with normal tissues. The meta-analysis approach was suitable and effective solution for identification of statistically significant miRNA meta-signature by combining several miRNA expression studies 34. However, to our knowledge, no such systematic review of miRNA profiling studies has been performed in gastric cancer.

The present systematic review identified one most consistently reported upregulated miRNA, miRNA-21, which was consistently reported to be differentially expressed in 10 studies followed by miR-25, miR-92, and miR-223 in eight studies. MiR-375 was reported to be consistently downregulated in six studies followed by miR-148a and miR-30d in five studies.

The most consistently reported differentially expressed miRNA of upregulation in our systematic review was miR-21 which had an oncogenic activity. The upregulation of miR-21 significantly promoted cell proliferation and revealed a higher proportion of cells at S phase and knockdown of miR-21 expression resulted in cell-cycle arrest at G2/M phase and inhibited cell proliferation 35. Lee 36 showed higher expression of miR-21 in breast cancer tissues than in normal tissues and the high miR-21 expression was associated with mastectomy, larger tumor size, higher stage, higher grade, estrogen receptor (ER) negative, human epidermal growth factor receptor 2 (HER2) positive. From our study after miR-21, the most consistently reported upregulated microRNAs in eight studies were miR-25, miR-92, and miR-223. The up-regulation of miR-25 was significantly correlated with the status of lymph node metastasis and TNM (Tumor, Node, and Metastasis) stage and overexpression markedly promoted migration and invasion of esophageal squamous cell carcinoma 37. Previous miRNA expression analysis had shown high expression of miR-25 in colorectal carcinoma 38. miR-92a was implicated in human hepatocellular carcinoma development and miR-92a in human blood had the potential to be a noninvasive molecular marker for the diagnosis of human hepatocellular carcinoma 39. Expression profiling studies had shown that miR-92 is overexpressed in oral carcinoma 40. The miR-223 expression was significantly higher in cancerous tissues than in the corresponding normal tissues in esophageal squamous cell carcinoma and high miR-223 expression demonstrated a significantly poorer prognosis than those with low expression 41.

The most consistently reported differentially expressed microRNA of downregulation in our systematic review is miR-375 which has an antioncogenic activity. The overexpressed miR-375 suppressed cell proliferation, blocked G1-to S cell-cycle transition, and inhibited cell migration and invasion in human cervical SiHa and CaSki cells, suggesting that miR-375 is a candidate tumor suppressor 42. In this systematic review study, we found miR-148a and miR-30d were consistently reported downregulated in five studies. Liffers 43 found that miR-148a exhibited a significant fourfold downregulation in pancreatic ductal adenocarcinoma as opposed to normal pancreatic ductal cells and observed that stable lentiviral mediated overexpression of miR-148a in pancreatic cancer cell line, inhibited tumor cell growth and colony formation. Zhao 44 observed a decrease in miR-30d expression in the islets of diabetic db/db mice, in which MAP4K4 expression level was elevated and overexpression of miR-30d protected *β*-cells against TNF-*α* suppression on both insulin transcription and insulin secretion.

The MAPK pathway is an important pathway in causing the gastric cancer. From our study, we found one important gene, TNF receptor-associated factor 6 (TRAF6), involved in MAPK signaling pathway was targeted by downregulated microRNA. Comprehensive analysis using cDNA microarray showed that RUNX3 upregulated 17 apoptosis-related genes including TRAF6 in MKN-1 cells 45. A positive correlation between TRAF6 and ubiquitin expression was found suggesting TRAF6 may upregulate ubiquitin activity in cancer cachexia 46. Inhibition of TRAF6 in human lung cancer cells suppressed NF-*κ*B activation, anchorage-independent growth and tumor formation 47. The genes such as Ras, Raf, MEK, MAPK, ERK that were targeted by upregulated microRNAs in our analysis had significant role in the pathway. MAPK pathway had been often found to be deregulated in cancers and consists of several kinases (Ras, Raf, MEK) that were activated by phosphorylation upon ligand binding to a membrane receptor, ending up in the activation of several proteins involved in processes of cell invasion, apoptosis, transcription, survival, and drug resistance 48. Ras/MAPK activation was found to be associated with cell proliferation and gastric carcinomas 49. ERK1/2 the final effectors of this pathway, were also found to be activated in gastric cancers 50.

In our study, 51 microRNAs among 352 microRNAs were reported to be inconsistent expressed in at least two independent studies. An elegant way to identify inconsistent studies is to correlate all studies with each other (study–study inconsistent miRNA matrix). Table S7 shows that study 15, 23, 18, and 16 have the higher inconsistency with other studies; especially reference 15 reported 13 microRNAs inconsistent with reference 18. The study number is the same as the reference number shown in the main manuscript. Previous studies have shown that some differentially expressed miRNAs are reported in inconsistent direction among various studies when using microRNA arrays for a particular disease such as a cancer 34,51. The other three possible reasons for these observed inconsistencies are: the lack of sufficient relevant clinical data, the heterogeneous tissue samples, and the poor study design.

In conclusion, from our systematic review study we identified that miR-21 is the most consistently reported upregulated microRNA and miR-375 is the most consistently reported downregulated microRNA in gastric cancer. The findings of our present study may have important clinical significance. The results of this systematic review of GC miRNA expression profiling studies provide some clues of the potential biomarkers in gastric cancer. The most consistently reported differentially expressed microRNAs may be used as efficient biomarkers and therapeutic targets. Our study found several promising miRNAs that had been consistently reported. However, still more investigations are needed for the clinical studies focusing on these miRNAs in order to understand the potential roles of these microRNAs in gastric cancer.

## Conflict of Interest

None declared.

## Supporting Information

**Figure S1.** The top 20 significant GO terms for downregulated miRNAs target genes. **Figure S2.** The top 20 significant KEGG pathways for downregulated miRNAs target genes. **Table S1.** Inconsistently reported miRNAs in profiling studies (gastric cancer tissue vs. normal). **Table S2.** Experimentally validated target genes for microRNAs downregulated in gastric cancer. **Table S3.** Experimentally validated target genes for microRNAs upregulated in gastric cancer. **Table S4.** Experimentally validated target genes for microRNAs up- and downregulated in gastric cancer. **Table S5.** Gene ontology terms of target genes for downregulated miRNAs in gastric cancer. **Table S6.** KEGG pathways of target genes for downregulated miRNAs in gastric cancer. **Table S7.** The number of microRNAs mentioned in two references with inconsistent direction (study–study inconsistent miRNA matrix).

## References

[b1] Ferlay J, Shin HR, Bray F, Forman D, Mathers C, Parkin DM (2010). Estimates of worldwide burden of cancer in 2008: GLOBOCAN 2008. Int. J. Cancer.

[b2] Coburn NG (2009). Lymph nodes and gastric cancer. J. Surg. Oncol.

[b3] Konishi H, Ichikawa D, Komatsu S, Shiozaki A, Tsujiura M, Takeshita H (2012). Detection of gastric cancer-associated microRNAs on microRNA microarray comparing pre- and post-operative plasma. Br. J. Cancer.

[b4] Li BS, Zhao YL, Guo G, Li W, Zhu ED, Luo X (2012). Plasma microRNAs, miR-223, miR-21 and miR-218, as novel potential biomarkers for gastric cancer detection. PLoS One.

[b5] Jiang Z, Guo J, Xiao B, Miao Y, Huang R, Li D (2010). Increased expression of miR-421 in human gastric carcinoma and its clinical association. J. Gastroenterol.

[b6] Bartel DP (2004). MicroRNAs: genomics, biogenesis, mechanism, and function. Cell.

[b7] Esquela-Kerscher A, Slack FJ (2006). Oncomirs—microRNAs with a role in cancer. Nat. Rev. Cancer.

[b8] Alvarez-Garcia I, Miska EA (2005). MicroRNA functions in animal development and human disease. Development.

[b9] Zhu W, Zhu D, Lu S, Wang T, Wang J, Jiang B (2012). miR-497 modulates multidrug resistance of human cancer cell lines by targeting BCL2. Med. Oncol.

[b10] Guo J, Miao Y, Xiao B, Huan R, Jiang Z, Meng D (2009). Differential expression of microRNA species in human gastric cancer versus non-tumorous tissues. J. Gastroenterol. Hepatol.

[b11] Song MY, Pan KF, Su HJ, Zhang L, Ma JL, Li JY (2012). Identification of serum microRNAs as novel non-invasive biomarkers for early detection of gastric cancer. PLoS One.

[b12] Griffith OL, Melck A, Jones SJ, Wiseman SM (2006). Meta-analysis and meta-review of thyroid cancer gene expression profiling studies identifies important diagnostic biomarkers. J. Clin. Oncol.

[b13] Chan SK, Griffith OL, Tai IT, Jones SJ (2008). Meta-analysis of colorectal cancer gene expression profiling studies identifies consistently reported candidate biomarkers. Cancer Epidemiol. Biomarkers Prev.

[b14] Carvalho J, van Grieken NC, Pereira PM, Sousa S, Tijssen M, Buffart TE (2012). Lack of microRNA-101 causes E-cadherin functional deregulation through EZH2 up-regulation in intestinal gastric cancer. J. Pathol.

[b15] Kim CH, Kim HK, Rettig RL, Kim J, Lee ET, Aprelikova O (2011). miRNA signature associated with outcome of gastric cancer patients following chemotherapy. BMC Med. Genomics.

[b16] Li X, Luo F, Li Q, Xu M, Feng D, Zhang G (2011). Identification of new aberrantly expressed miRNAs in intestinal-type gastric cancer and its clinical significance. Oncol. Rep.

[b17] Li X, Zhang Y, Zhang H, Liu X, Gong T, Li M (2011). miRNA-223 promotes gastric cancer invasion and metastasis by targeting tumor suppressor EPB41L3. Mol. Cancer Res.

[b18] Oh HK, Tan AL, Das K, Ooi CH, Deng NT, Tan IB (2011). Genomic loss of miR-486 regulates tumor progression and the OLFM4 antiapoptotic factor in gastric cancer. Clin. Cancer Res.

[b19] Tchernitsa O, Kasajima A, Schafer R, Kuban RJ, Ungethum U, Gyorffy B (2010). Systematic evaluation of the miRNA-ome and its downstream effects on mRNA expression identifies gastric cancer progression. J. Pathol.

[b20] Ding L, Xu Y, Zhang W, Deng Y, Si M, Du Y (2010). MiR-375 frequently downregulated in gastric cancer inhibits cell proliferation by targeting JAK2. Cell Res.

[b21] Tsukamoto Y, Nakada C, Noguchi T, Tanigawa M, Nguyen LT, Uchida T (2010). MicroRNA-375 is downregulated in gastric carcinomas and regulates cell survival by targeting PDK1 and 14-3-3zeta. Cancer Res.

[b22] Ueda T, Volinia S, Okumura H, Shimizu M, Taccioli C, Rossi S (2010). Relation between microRNA expression and progression and prognosis of gastric cancer: a microRNA expression analysis. Lancet Oncol.

[b23] Yao Y, Suo AL, Li ZF, Liu LY, Tian T, Ni L (2009). MicroRNA profiling of human gastric cancer. Mol. Med. Report.

[b24] Luo H, Zhang H, Zhang Z, Zhang X, Ning B, Guo J (2009). Down-regulated miR-9 and miR-433 in human gastric carcinoma. J. Exp. Clin. Cancer Res.

[b25] Liu T, Tang H, Lang Y, Liu M, Li X (2009). MicroRNA-27a functions as an oncogene in gastric adenocarcinoma by targeting prohibitin. Cancer Lett.

[b26] Petrocca F, Visone R, Onelli MR, Shah MH, Nicoloso MS, de Martino I (2008). E2F1-regulated microRNAs impair TGFbeta-dependent cell-cycle arrest and apoptosis in gastric cancer. Cancer Cell.

[b27] Volinia S, Calin GA, Liu CG, Ambs S, Cimmino A, Petrocca F (2006). A microRNA expression signature of human solid tumors defines cancer gene targets. Proc. Natl Acad. Sci. USA.

[b28] Hsu SD, Lin FM, Wu WY, Liang C, Huang WC, Chan WL (2011). miRTarBase: a database curates experimentally validated microRNA-target interactions. Nucleic Acids Res.

[b29] Harris MA, Clark J, Ireland A, Lomax J, Ashburner M, Foulger R (2004). The gene ontology (GO) database and informatics resource. Nucleic Acids Res.

[b30] Kanehisa M, Goto S, Kawashima S, Okuno Y, Hattori M (2004). The KEGG resource for deciphering the genome. Nucleic Acids Res.

[b31] Dennis G, Sherman BT, Hosack DA, Yang J, Gao W, Lane HC (2003). DAVID: Database for Annotation, Visualization, and Integrated Discovery. Genome Biol.

[b32] Ma Y, Zhang P, Yang J, Liu Z, Yang Z, Qin H (2012). Candidate microRNA biomarkers in human colorectal cancer: systematic review profiling studies and experimental validation. Int. J. Cancer.

[b33] Guan P, Yin Z, Li X, Wu W, Zhou B (2012). Meta-analysis of human lung cancer microRNA expression profiling studies comparing cancer tissues with normal tissues. J. Exp. Clin. Cancer Res.

[b34] Vosa U, Vooder T, Kolde R, Vilo J, Metspalu A, Annilo T (2013). Meta-analysis of microRNA expression in lung cancer. Int. J. Cancer.

[b35] Zhong Z, Dong Z, Yang L, Gong Z (2012). miR-21 induces cell cycle at S phase and modulates cell proliferation by down-regulating hMSH2 in lung cancer. J. Cancer Res. Clin. Oncol.

[b36] Lee JA, Lee HY, Lee ES, Kim I, Bae JW (2011). Prognostic implications of microRNA-21 overexpression in invasive ductal carcinomas of the breast. J. Breast Cancer.

[b37] Xu X, Chen Z, Zhao X, Wang J, Ding D, Wang Z (2012). MicroRNA-25 promotes cell migration and invasion in esophageal squamous cell carcinoma. Biochem. Biophys. Res. Commun.

[b38] Nishida N, Nagahara M, Sato T, Mimori K, Sudo T, Tanaka F (2012). Microarray analysis of colorectal cancer stromal tissue reveals upregulation of two oncogenic miRNA clusters. Clin. Cancer Res.

[b39] Shigoka M, Tsuchida A, Matsudo T, Nagakawa Y, Saito H, Suzuki Y (2010). Deregulation of miR-92a expression is implicated in hepatocellular carcinoma development. Pathol. Int.

[b40] Scapoli L, Palmieri A, Lo Muzio L L, Pezzetti F, Rubini C, Girardi A (2010). MicroRNA expression profiling of oral carcinoma identifies new markers of tumor progression. Int. J. Immunopathol. Pharmacol.

[b41] Kurashige J, Watanabe M, Iwatsuki M, Kinoshita K, Saito S, Hiyoshi Y (2012). Overexpression of microRNA-223 regulates the ubiquitin ligase FBXW7 in oesophageal squamous cell carcinoma. Br. J. Cancer.

[b42] Wang F, Li Y, Zhou J, Xu J, Peng C, Ye F (2011). miR-375 is down-regulated in squamous cervical cancer and inhibits cell migration and invasion via targeting transcription factor SP1. Am. J. Pathol.

[b43] Liffers ST, Munding JB, Vogt M, Kuhlmann JD, Verdoodt B, Nambiar S (2011). MicroRNA-148a is down-regulated in human pancreatic ductal adenocarcinomas and regulates cell survival by targeting CDC25B. Lab. Invest.

[b44] Zhao X, Mohan R, Ozcan S, Tang X (2012). MicroRNA-30d induces insulin transcription factor MafA and insulin production by targeting mitogen-activated protein 4 kinase 4 (MAP4K4) in pancreatic beta-cells. J. Biol. Chem.

[b45] Nagahama Y, Ishimaru M, Osaki M, Inoue T, Maeda A, Nakada C (2008). Apoptotic pathway induced by transduction of RUNX3 in the human gastric carcinoma cell line MKN-1. Cancer Sci.

[b46] Sun YS, Ye ZY, Qian ZY, Xu XD, Hu JF (2012). Expression of TRAF6 and ubiquitin mRNA in skeletal muscle of gastric cancer patients. J. Exp. Clin. Cancer Res.

[b47] Starczynowski DT, Lockwood WW, Delehouzee S, Chari R, Wegrzyn J, Fuller M (2011). TRAF6 is an amplified oncogene bridging the RAS and NF-kappaB pathways in human lung cancer. J. Clin. Invest.

[b48] Kim EK, Choi EJ (2010). Pathological roles of MAPK signaling pathways in human diseases. Biochim. Biophys. Acta.

[b49] Regalo G, Resende C, Wen X, Gomes B, Duraes C, Seruca R (2010). C/EBP alpha expression is associated with homeostasis of the gastric epithelium and with gastric carcinogenesis. Lab. Invest.

[b50] Liang B, Wang S, Zhu XG, Yu YX, Cui ZR, Yu YZ (2005). Increased expression of mitogen-activated protein kinase and its upstream regulating signal in human gastric cancer. World J. Gastroenterol.

[b51] Gong X, Wu R, Wang H, Guo X, Wang D, Gu Y (2011). Evaluating the consistency of differential expression of microRNA detected in human cancers. Mol. Cancer Ther.

