# Supplementary material for: A systematic review of microRNA expression profiling studies in human gastric cancer
Source: Cancer Med. 2014 Jun 5;3(4):878–88. doi: 10.1002/cam4.246 (PMC4303155; doi:10.1002/cam4.246)
Supplement: Supplementary file 1 [file cam40003-0878-sd1.docx]

**SUPPLEMENTARY FIGURE LEGENDS**

**Supplementary Figure S1**. The top 20 significant GO terms for down-regulated miRNAs target genes

**Supplementary Figure S2**. The top 20 significant KEGG pathways for down-regulated miRNAs target genes

**SUPPLEMENTARY TABLE CAPTIONS**

**Supplementary Table S1.** Inconsistently reported miRNAs in profiling studies (gastric cancer tissue versus normal)

**Supplementary Table S2.** Experimentally validated target genes for microRNAs down-regulated in gastric cancer

**Supplementary Table S3.** Experimentally validated target genes for microRNAs up-regulated in gastric cancer

**Supplementary Table S4.** Experimentally validated target genes for microRNAs up- and down-regulated in gastric cancer

**Supplementary Table S5.** Gene ontology terms of target genes for down-regulated miRNAs in gastric cancer

**Supplementary Table S6.** KEGG pathways of target genes for down-regulated miRNAs in gastric cancer

**Supplementary Table S7.** The number of microRNAs mentioned in two references with inconsistent direction (study-study inconsistent miRNA matrix)

**Supplementary Figure S1**. The top 20 significant GO terms for down-regulated miRNAs target genes

**Supplementary Figure S2**. The top 20 significant KEGG pathways for down-regulated miRNAs target genes

**Supplementary Table S1.** Inconsistently reported miRNAs in profiling studies (gastric cancer tissue versus normal)

| miRNA name | Direction of  expression | Reference | | Total number of tissue  samples tested |  |
| --- | --- | --- | --- | --- | --- |
| miR-107 | ↑  ↓ | [15-19](#_ENREF_15), [21](#_ENREF_21), [26](#_ENREF_26), [27](#_ENREF_27)  [23](#_ENREF_23) | | 356  6 |  |
| miR-103 | ↑  ↓ | [15](#_ENREF_15), [17-19](#_ENREF_17), [21](#_ENREF_21), [26](#_ENREF_26), [27](#_ENREF_27)  [23](#_ENREF_23) | | 344  6 |  |
| miR-125b | ↑  ↓ | [17-19](#_ENREF_17), [26](#_ENREF_26), [27](#_ENREF_27)  [16](#_ENREF_16) | | 193  12 |  |
| miR-30a | ↑  ↓ | [24](#_ENREF_24)  [15](#_ENREF_15), [17](#_ENREF_17), [18](#_ENREF_18), [21](#_ENREF_21), [22](#_ENREF_22) | | 27  604 |  |
| miR-222 | ↑  ↓ | [16](#_ENREF_16), [17](#_ENREF_17), [19](#_ENREF_19), [21](#_ENREF_21), [27](#_ENREF_27)  [18](#_ENREF_18) | | 112  80 |  |
| miR-181b | ↑  ↓ | [15](#_ENREF_15), [22](#_ENREF_22), [26](#_ENREF_26)  [17](#_ENREF_17), [25](#_ENREF_25) | | 517  28 |  |
| miR-320 | ↑  ↓ | [15](#_ENREF_15), [19](#_ENREF_19), [21](#_ENREF_21), [24](#_ENREF_24)  [16](#_ENREF_16) | | 190  12 |  |
| miR-24 | ↑  ↓ | [16](#_ENREF_16), [19](#_ENREF_19), [21](#_ENREF_21), [27](#_ENREF_27)  [23](#_ENREF_23) | | 92  6 |  |
| miR-19b | ↑  ↓ | [15](#_ENREF_15), [16](#_ENREF_16), [21](#_ENREF_21), [22](#_ENREF_22)  [24](#_ENREF_24) | | 516  27 |  |
| miR-451 | ↑  ↓ | [15](#_ENREF_15), [16](#_ENREF_16), [18](#_ENREF_18)  [14](#_ENREF_14), [22](#_ENREF_22) | | 216  400 |  |
| let-7a | ↑  ↓ | [18](#_ENREF_18), [19](#_ENREF_19)  [15](#_ENREF_15), [17](#_ENREF_17) | | 92  144 |  |
| miR-183 | ↑  ↓ | [15](#_ENREF_15), [17](#_ENREF_17), [23](#_ENREF_23)  [25](#_ENREF_25) | | 142  8 |  |
| miR-125a | ↑  ↓ | [23](#_ENREF_23)  [15](#_ENREF_15), [16](#_ENREF_16), [18](#_ENREF_18) | | 6  216 |  |
| miR-96 | ↑  ↓ | [15](#_ENREF_15), [16](#_ENREF_16)  [26](#_ENREF_26), [27](#_ENREF_27) | | 136  81 |  |
| miR-34a | ↑  ↓ | [18](#_ENREF_18), [21](#_ENREF_21), [23](#_ENREF_23)  [15](#_ENREF_15) | | 113  124 |  |
| miR-210 | ↑  ↓ | [15](#_ENREF_15)  [17](#_ENREF_17), [25](#_ENREF_25) | | 124  28 |  |
| miR-145 | ↑  ↓ | [19](#_ENREF_19), [23](#_ENREF_23)  [15](#_ENREF_15) | | 18  124 |  |
| miR-575 | ↑  ↓ | [23](#_ENREF_23)  [18](#_ENREF_18), [24](#_ENREF_24) | | 6  107 |  |
| miR-146b | ↑  ↓ | [18](#_ENREF_18), [23](#_ENREF_23)  [15](#_ENREF_15) | | 86  124 |  |
| miR-345 | ↑  ↓ | [15](#_ENREF_15), [22](#_ENREF_22)  [18](#_ENREF_18) | | 477  80 |  |
| miR-30b | ↑  ↓ | [19](#_ENREF_19)  [15](#_ENREF_15), [22](#_ENREF_22) | | 12  477 |  |
| miR-181c | ↑  ↓ | [22](#_ENREF_22)  [18](#_ENREF_18), [25](#_ENREF_25) | | 353  88 |  |
| miR-28 | ↑  ↓ | [18](#_ENREF_18)  [15](#_ENREF_15), [18](#_ENREF_18) | | 80  204 |  |
| miR-182 | ↑  ↓ | [16](#_ENREF_16), [23](#_ENREF_23)  [25](#_ENREF_25) | | 18  8 |  |
| miR-486-5p | ↑  ↓ | [15](#_ENREF_15)  [16](#_ENREF_16), [18](#_ENREF_18) | | 124  92 |  |
| let-7g | ↑  ↓ | [18](#_ENREF_18)  [15](#_ENREF_15), [16](#_ENREF_16) | | 80  136 |  |
| miR-143 | ↑  ↓ | [19](#_ENREF_19)  [15](#_ENREF_15), [16](#_ENREF_16) | | 12  136 |  |
| miR-141 | ↑  ↓ | [16](#_ENREF_16)  [20](#_ENREF_20), [23](#_ENREF_23) | | 12  18 |  |
| miR-16 | ↑  ↓ | [18](#_ENREF_18), [21](#_ENREF_21)  [16](#_ENREF_16) | | 107  12 |  |
| miR-212 | ↑  ↓ | [24](#_ENREF_24)  [15](#_ENREF_15), [26](#_ENREF_26) | | 27  164 |  |
| miR-26b | ↑  ↓ | [24](#_ENREF_24)  [15](#_ENREF_15) | | 27  124 |  |
| miR-29b | ↑  ↓ | [18](#_ENREF_18)  [22](#_ENREF_22) | | 80  353 |  |
| miR-15b | ↑  ↓ | [21](#_ENREF_21)  [23](#_ENREF_23) | | 27  6 |  |
| miR-429 | ↑  ↓ | [21](#_ENREF_21)  [15](#_ENREF_15) | | 27  124 |  |
| miR-338 | ↑  ↓ | [23](#_ENREF_23)  [24](#_ENREF_24) | | 6  27 |  |
| miR-596 | ↑  ↓ | [15](#_ENREF_15)  [18](#_ENREF_18) | | 124  80 |  |
| miR-339 | ↑  ↓ | [15](#_ENREF_15)  [26](#_ENREF_26) | | 124  40 |  |
| miR-503 | ↑  ↓ | [15](#_ENREF_15)  [24](#_ENREF_24) | | 124  27 |  |
| miR-545 | ↑  ↓ | [15](#_ENREF_15)  [24](#_ENREF_24) | | 124  27 |  |
| miR-623 | ↑  ↓ | [15](#_ENREF_15)  [18](#_ENREF_18) | | 124  80 |  |
| miR-550 | ↑  ↓ | [15](#_ENREF_15)  [18](#_ENREF_18) | | 124  80 |  |
| miR-626 | ↑  ↓ | [23](#_ENREF_23)  [15](#_ENREF_15) | | 6  124 |  |
| miR-193b | ↑  ↓ | [23](#_ENREF_23)  [18](#_ENREF_18) | | 6  80 |  |
| let-7f | ↑  ↓ | [18](#_ENREF_18)  [15](#_ENREF_15) | | 80  124 |  |
| let-7d | ↑  ↓ | [18](#_ENREF_18)  [15](#_ENREF_15) | | 80  124 |  |
| let-7e | ↑  ↓ | [18](#_ENREF_18)  [15](#_ENREF_15) | | 80  124 |  |
| miR-138 | ↑  ↓ | [23](#_ENREF_23)  [27](#_ENREF_27) | | 6  41 |  |
| miR-203 | ↑  ↓ | [16](#_ENREF_16)  [15](#_ENREF_15) | 12  124 | | |
| miR-147b | ↑  ↓ | [23](#_ENREF_23)  [16](#_ENREF_16) | 6  12 | | |
| miR-196a* | ↑  ↓ | [23](#_ENREF_23)  [16](#_ENREF_16) | 6  12 | | |
| miR-33a | ↑  ↓ | [16](#_ENREF_16)  [14](#_ENREF_14) | 12  47 | | |

**Supplementary Table S2.** Experimentally validated target genes for microRNAs down-regulated in gastric cancer

| **MicroRNA** | **Target gene** | **Entrez gene ID** | **Gene name** | **Validation method** |
| --- | --- | --- | --- | --- |
| hsa-miR-101 | *APP* | 351 | amyloid beta (A4) precursor protein | qRT-PCR//Luciferase reporter assay//Western blot |
| hsa-miR-101 | *ARID1A* | 8289 | AT rich interactive domain 1A (SWI-like) | Western blot |
| hsa-miR-101 | *ATM* | 472 | ataxia telangiectasia mutated | Luciferase reporter assay//qRT-PCR |
| hsa-miR-101 | *ATP5B* | 506 | ATP synthase, H+ transporting, mitochondrial F1 complex, beta polypeptide | qRT-PCR//Western blot |
| hsa-miR-101 | *ATXN1* | 6310 | ataxin 1 | Luciferase reporter assay//qRT-PCR//Western blot |
| hsa-miR-101 | *COX2* | 4513 | mitochondrially encoded cytochrome c oxidase II | Microarray |
| hsa-miR-101 | *DUSP1* | 1843 | dual specificity phosphatase 1 | Luciferase reporter assay//qRT-PCR//Western blot |
| hsa-miR-101 | *EED* | 8726 | embryonic ectoderm development | Western blot |
| hsa-miR-101 | *EZH2* | 2146 | enhancer of zeste homolog 2 (Drosophila) | Immunoblot//Immunohistochemistry//Luciferase reporter assay//Microarray//qRT-PCR |
| hsa-miR-101 | *FBN2* | 2201 | fibrilin 2 | Western blot |
| hsa-miR-101 | *FOS* | 2353 | FBJ murine osteosarcoma viral oncogene homolog | Western blot |
| hsa-miR-101 | *MCL1* | 4170 | myeloid cell leukemia sequence 1 (BCL2-related) | Luciferase reporter assay//qRT-PCR//Western blot |
| hsa-miR-101 | *MYCN* | 4613 | v-muc myelocytomatosis viral related oncogene, neuroblastoma derived (avian) | Luciferase reporter assay |
| hsa-miR-101 | *PTGS2* | 5743 | prostaglandin-endoperoxidase synthase 2 (prostaglandin G/H synthase and cyclooxygenase) | qRT-PCR//Luciferase reporter assay//Western blot |
| hsa-miR-101 | *SUZ12* | 23512 | suppressor of zeste 12 homolog (Drosophila) | Western blot |
| hsa-miR-101 | *ATG4D* | 84971 | autophagy related 4D, cycteine peptidase | Luciferase reporter assay//Microarray//qRT-PCR//Western blot |
| hsa-miR-101 | *RAB5A* | 5868 | RAB5A, member RAS oncogene family | Luciferase reporter assay//Microarray//qRT-PCR//Western blot |
| hsa-miR-101 | *STMN1* | 3925 | stathmin 1 | Luciferase reporter assay//Microarray//qRT-PCR//Western blot |
| hsa-miR-133b | *BCL2L2* | 599 | BCL2-like 2 | Western blot |
| hsa-miR-133b | *FSCN1* | 6624 | fascin homolog 1, actin-bunding protein (Strongylocentrous purpuratus) | Luciferase reporter assay//qRT-PCR//Western blot |
| hsa-miR-133b | *IGF1R* | 3480 | insulin-like growth factor 1 receptor | Luciferase reporter assay |
| hsa-miR-133b | *KCNH2* | 3757 | potassium voltage-gated channel, subfamily H (eag-related), member 2 | Luciferase reporter assay//qRT-PCR//Western blot |
| hsa-miR-133b | *MCL1* | 4170 | myeloid cell leukemia sequence 1 (BCL2-related) | Western blot |
| hsa-miR-133b | *PITX3* | 5309 | paired-like homeodomain 3 | FACS//Luciferase reporter assay |
| hsa-miR-133b | *PKM2* | 5315 | pyruvate kinase, muscle | Immunohistochemistry |
| hsa-miR-146a | *BRCA1* | 672 | breast cancer 1, early onset | Luciferase reporter assay |
| hsa-miR-146a | *BRCA2* | 675 | breast cancer 2, early onset | Luciferase reporter assay |
| hsa-miR-146a | *CCNA2* | 890 | cyclin A2 | Microarray//qRT-PCR//Western blot |
| hsa-miR-146a | *CD40LG* | 959 | CD40 ligand | ELISA//Luciferase reporter assay//qRT-PCR//Western blot |
| hsa-miR-146a | *CDKN1A* | 1026 | cyclin-dependent kinase inhibitor 1A (p21, Cip1) | Luciferase reporter assay |
| hsa-miR-146a | *CDKN3* | 1033 | cyclin-dependent kinase inhibitor 3 | Western blot |
| hsa-miR-146a | *CFH* | 3075 | complement factor H | Western blot |
| hsa-miR-146a | *CXCR4* | 7852 | chemokine (C-X-C motif) receptor 4 | qRT-PCR//Luciferase reporter assay//Western blot |
| hsa-miR-146a | *ERBB4* | 2066 | v-erb-a erythroblastic leukemia viral oncogene homolog 4 (avian) | Luciferase reporter assay//Microarray//Western blot |
| hsa-miR-146a | *FADD* | 8772 | Fas (TNFRSF6)-associated via death domain | Luciferase reporter assay |
| hsa-miR-146a | *FAF1* | 11124 | Fas (TNFRSF6)-associated factor 1 | GFP reporter assay//qRT-PCR |
| hsa-miR-146a | *FAS* | 355 | Fas cell surface death receptor | Luciferase reporter assay//Western blot |
| hsa-miR-146a | *IL8* | 3576 | interleukin 8 | Western blot//Northern blot |
| hsa-miR-146a | *IRAK1* | 3654 | interleukin-1 receptor-associated kinase 1 | Luciferase reporter assay//Western blot//Northern blot//qRT-PCR |
| hsa-miR-146a | *IRAK2* | 3656 | interleukin-1 receptor-associated kinase 2 | qRT-PCR//Western blot |
| hsa-miR-146a | *KIF22* | 3835 | kinesin family member 22 | Western blot |
| hsa-miR-146a | *MTA2* | 9219 | metastasis associated family, member 2 | qRT-PCR//Western blot |
| hsa-miR-146a | *NFKB1* | 4790 | nuclear factor of kappa light polypeptide gene enhancer in B-cells 1 | Luciferase reporter assay |
| hsa-miR-146a | *PA2G4* | 5036 | proliferation-associated 2G4, 38kDa | Microarray//qRT-PCR//Western blot |
| hsa-miR-146a | *ROCK1* | 6093 | Rho-associated, coiled-coil containing protein kinase 1 | flow//Western blot//Northern blot |
| hsa-miR-146a | *TLR2* | 7097 | toll-like receptor 2 | qRT-PCR//flow//Luciferase reporter assay//Western blot |
| hsa-miR-146a | *TRAF6* | 7189 | TNF receptor-associated factor 6, E3 ubiquitin protein ligase | Western blot//Northern blot |
| hsa-miR-146a | *EGFR* | 1956 | epidermal growth factor receptor | Luciferase reporter assay//Western blot//qRT-PCR |
| hsa-miR-146a | *SMAD4* | 4089 | SMAD family member 4 | Immunohistochemistry//Luciferase reporter assay//Microarray//qRT-PCR//Western blot |
| hsa-miR-146a | *TLR4* | 7099 | toll-like receptor 4 | ELISA//Luciferase reporter assay//Microarray//qRT-PCR//Western blot |
| hsa-miR-148a | *CCKBR* | 887 | cholecystokinin B receptor | Luciferase reporter assay//Microarray |
| hsa-miR-148a | *DNMT1* | 1786 | DNA (cytosine-5)-methyltransferase 1 | Luciferase reporter assay//Flow//qRT-PCR//Western blot |
| hsa-miR-148a | *DNMT3B* | 1789 | DNA (cytosine-5-)-methyltransferase 3 beta | GFP reporter assay//Western blot//qRT-PCR |
| hsa-miR-148a | *HLA-G* | 3135 | major histocompatibility complex, class I, G | Luciferase reporter assay |
| hsa-miR-148a | *NR1I2* | 8856 | nuclear receptor subfamily1, group I, member 2 | Luciferase reporter assay//qRT-PCR |
| hsa-miR-148a | *RPS6KA5* | 9252 | ribosomal protein S6 kinase, 90kDa, polypeptide 5 | qRT-PCR//Luciferase reporter assay//Western blot |
| hsa-miR-148a | *TGIF2* | 60436 | TGFB-induced factor homeobox 2 | Luciferase reporter assay//Western blot |
| hsa-miR-150 | *EGR2* | 1959 | early growth response 2 | Luciferase reporter assay//semi-qRT-PCR//Western blot |
| hsa-miR-150 | *IGF2* | 3481 | insulin-like growth factor 2 (somatomedin A) | Luciferase reporter assay |
| hsa-miR-150 | *MYB* | 4602 | v-myb myeloblastosis viral oncogene homolog (avian) | Western blot |
| hsa-miR-150 | *P2RX7* | 5027 | purigenic receptor P2X, ligand-gated ion channel, 7 | qRT-PCR//Luciferase reporter assay |
| hsa-miR-150 | *VEGFA* | 7422 | vascular endothelial growth factor A | ELISA//Luciferase reporter assay |
| hsa-miR-150 | *MUC4* | 4585 | mucin 4, cell surface associated | Luciferase reporter assay//qRT-PCR//Western blot |
| hsa-miR-152 | *DNMT1* | 1786 | DNA (cytosine-5)-methyltransferase 1 | Luciferase reporter assay |
| hsa-miR-152 | *HLA-G* | 3135 | major histocompatibility complex, class I, G | Luciferase reporter assay |
| hsa-miR-155 | *AGTR1* | 185 | angiotensin II receptor, type 1 | Luciferase reporter assay//Northern blot//qRT-PCR//Western blot |
| hsa-miR-155 | *AMIGO2* | 347902 | adhesion molecule with Ig-like domain 2 | pSILAC |
| hsa-miR-155 | *ANKFY1* | 51479 | ankyrin repeat and FYVE domain containing 1 | pSILAC |
| hsa-miR-155 | *APC* | 324 | adenomatous polyposis coli | Luciferase reporter assay//Western blot |
| hsa-miR-155 | *ARFIP1* | 27236 | ADP-ribosylation factor interacting protein 1 | pSILAC |
| hsa-miR-155 | *ARFIP2* | 23647 | ADP-ribosylation factor interacting protein 2 | pSILAC |
| hsa-miR-155 | *ARID2* | 196528 | AT rich interactive domain 2 (ARID, RFX-like) | Luciferase reporter assay |
| hsa-miR-155 | *ARL10* | 285598 | ADP-ribosylation factor-like 10 | pSILAC |
| hsa-miR-155 | *ARL5B* | 221079 | ADP-ribosylation factor-like 5B | pSILAC |
| hsa-miR-155 | *ATG3* | 64422 | autophagy related 3 | pSILAC |
| hsa-miR-155 | *ATP6V1C1* | 528 | ATPase, H+ transporting, lysosomal 42kDa, V1 subunit C1 | pSILAC |
| hsa-miR-155 | *BACH1* | 571 | BTB and CNC homology 1, basic leucine zipper transcription factor 1 | Immunohistochemistry//In situ hybridization//Luciferase reporter assay//Microarray//qRT-PCR//Western blot |
| hsa-miR-155 | *BCAT1* | 586 | branched chain amino-acid transaminase 1, cytosolic | Microarray |
| hsa-miR-155 | *BET1* | 10282 | Bet1 golgi vesicular membrane trafficking protein | pSILAC |
| hsa-miR-155 | *BRPF3* | 27154 | bromodomain and PHD finger containing, 3 | pSILAC |
| hsa-miR-155 | *C3orf58* | 205428 | chromosome 3 open reading frame 58 | pSILAC |
| hsa-miR-155 | *C5orf41* | 153222 | CREB3 regulatory factor | Microarray |
| hsa-miR-155 | *CAMTA1* | 23261 | calmodulin binding transcription activator 1 | Microarray |
| hsa-miR-155 | *CBFB* | 865 | core-binding factor, beta subunit | pSILAC |
| hsa-miR-155 | *CDK5RAP3* | 80279 | CDK5 regulatory aubunit associated protein 3 | pSILAC |
| hsa-miR-155 | *CEBPB* | 1051 | CCAAT/enhancer binding protein (C/EBP), beta | Luciferase reporter assay//qRT-PCR |
| hsa-miR-155 | *CHAF1A* | 10036 | chromatin assembly factor 1, subunit A (p150) | pSILAC |
| hsa-miR-155 | *CLDN1* | 9076 | claudin 1 | pSILAC |
| hsa-miR-155 | *CTLA4* | 1493 | cytotoxic T-lymphocyte-associated protein 4 | Microarray |
| hsa-miR-155 | *CTNNB1* | 1499 | catenin (cadherin-associated protein), beta 1 88kDa | pSILAC |
| hsa-miR-155 | *CUL4B* | 8450 | cullin 4B | pSILAC |
| hsa-miR-155 | *CUX1* | 1523 | cut-like homebox 1 | Microarray//qRT-PCR |
| hsa-miR-155 | *CYP51A1* | 1595 | cytochrome P450, family 51, subfamily A, polypeptide 1 | pSILAC |
| hsa-miR-155 | *CYR61* | 3491 | cycteine-rich, angiogenic inducer, 61 | Luciferase reporter assay//qRT-PCR//Western blot |
| hsa-miR-155 | *DCAF7* | 10238 | DDB1 and CUL4 associated factor 7 | pSILAC |
| hsa-miR-155 | *DET1* | 55070 | de-etiolated homolog 1 (Arabidopsis) | Luciferase reporter assay |
| hsa-miR-155 | *DHX40* | 79665 | DEAH (Asp-Glu-Ala-His) box polypeptide 40 | pSILAC |
| hsa-miR-155 | *DNAJB1* | 3337 | DnaJ (Hsp40) homolog, subfamily B, member 1 | pSILAC |
| hsa-miR-155 | *DNAJC19* | 131118 | DnaJ (Hsp40) homolog, subfamily C, member 19 | pSILAC |
| hsa-miR-155 | *DOCK4* | 9732 | dedicator of cytokinesis 4 | Microarray |
| hsa-miR-155 | *DPP7* | 29952 | dipeptidyl-peptidase 7 | pSILAC |
| hsa-miR-155 | *DSG2* | 1829 | desmoglein 2 | pSILAC |
| hsa-miR-155 | *EDEM3* | 80267 | ER degradation enhancer, mannosidase alpha-like 3 | pSILAC |
| hsa-miR-155 | *EDN1* | 1906 | endothelin 1 | Luciferase reporter assay |
| hsa-miR-155 | *EHD1* | 10938 | EH-domain containing 1 | Microarray |
| hsa-miR-155 | *ERMP1* | 79956 | endoplasmic reticulum metallopeptidase 1 | pSILAC |
| hsa-miR-155 | *ETS1* | 2113 | v-ets erythroblastosis virus E26 oncogene homolog 1 (avian) | Luciferase reporter assay//Microarray//Northern blot//qRT-PCR//Western blot |
| hsa-miR-155 | *FADS1* | 3992 | fatty acid desaturase 1 | pSILAC |
| hsa-miR-155 | *FAR1* | 84188 | fatty acyl CoA reductase 1 | pSILAC |
| hsa-miR-155 | *FGF7* | 2252 | fibroblast growth factor 7 | Luciferase reporter assay |
| hsa-miR-155 | *FLI1* | 2313 | Friend leukemia virus integration 1 | Western blot |
| hsa-miR-155 | *FMNL2* | 114793 | formin-like 2 | pSILAC |
| hsa-miR-155 | *FOXO3* | 2309 | forkhead box O3 | qRT-PCR//Luciferase reporter assay//Western blot |
| hsa-miR-155 | *GNA13* | 10672 | guanine nucleotide binding protein (G protein), alpha 13 | pSILAC |
| hsa-miR-155 | *HIVEP2* | 3097 | human immunodeficiency virus type I enhancer binding protein 2 | Luciferase reporter assay |
| hsa-miR-155 | *HNRNPA3P1* | 10151 | heterogenous nuclear ribonucleoprotein A3 pseudogene 1 | Microarray |
| hsa-miR-155 | *HSD17B12* | 51144 | hydroxysteroid (17-beta) dehydrogenase 12 | pSILAC |
| hsa-miR-155 | *HSDL1* | 83693 | hydroxysteroid dehydrogenase like 1 | pSILAC |
| hsa-miR-155 | *ICAM1* | 3383 | intercellular adhesion molecule 1 | Luciferase reporter assay |
| hsa-miR-155 | *IFNGR1* | 3459 | interferon gamma receptor 1 | Flow//Luciferase reporter assay//Northern blot |
| hsa-miR-155 | *IKBIP* | 121457 | IKBKB interacting protein | pSILAC |
| hsa-miR-155 | *IKBKE* | 9641 | inhibitor of kappa light polypeptide gene enhancer in B-cells, kinase apsilon | qRT-PCR//Western blot |
| hsa-miR-155 | *INPP5D* | 3635 | inositol polyphosphate-5-phosphatase, 145kDa | qRT-PCR//Luciferase reporter assay//Western blot |
| hsa-miR-155 | *JARID2* | 3720 | jumonji, AT rich interactive domain 2 | qRT-PCR//Luciferase reporter assay |
| hsa-miR-155 | *KBTBD2* | 25948 | kelch repeat and BTB (POZ) domain containing 2 | Microarray |
| hsa-miR-155 | *KIAA0776* | 23376 | UFM1-specific ligase 1 | pSILAC |
| hsa-miR-155 | *KRAS* | 3845 | v-Ki-ras2 Kirsten rat sarcoma viral oncogene homolog | Microarray |
| hsa-miR-155 | *KRT80* | 144501 | keratin 80 | pSILAC |
| hsa-miR-155 | *LAT2* | 7462 | linker for activation of T cells family, member 2 | Microarray |
| hsa-miR-155 | *LCLAT1* | 253558 | lysocardiolipin acyltransferase 1 | pSILAC |
| hsa-miR-155 | *LDOC1* | 23641 | leucine zipper, down-regulated in cancer 1 | Luciferase reporter assay |
| hsa-miR-155 | *LOC150786* | 150786 | RAB6C-like | pSILAC |
| hsa-miR-155 | *LPL* | 4023 | lipoprotein lipase | pSILAC |
| hsa-miR-155 | *LY6K* | 54742 | lymphocyte antigen 6 complex, locus K | pSILAC |
| hsa-miR-155 | *MAFB* | 9935 | v-maf musculoaponeurotic fibrosarcoma oncogene homolog B (avian) | Microarray |
| hsa-miR-155 | *MATR3* | 9782 | matrin 3 | Luciferase reporter assay |
| hsa-miR-155 | *MECP2* | 4204 | methyl CpG binding protein 2 (Rett syndrome) | Immunohistochemistry//Luciferase reporter assay//qRT-PCR//Western blot |
| hsa-miR-155 | *MEIS1* | 4211 | Meis homebox 1 | Luciferase reporter assay//qRT-PCR//Western blot |
| hsa-miR-155 | *METTL7A* | 25840 | methyltransferase like 7A | pSILAC |
| hsa-miR-155 | *MLH1* | 4292 | mutL homolog 1, colon cancer, nonpolyposis type 2 (E.coli) | Luciferase reporter assay//Western blot//Northern blot |
| hsa-miR-155 | *MOSC1* | 64757 | mitochondrial amidoxime reducing component 1 | pSILAC |
| hsa-miR-155 | *MOSPD2* | 158747 | motile sperm domain containing 2 | pSILAC |
| hsa-miR-155 | *MPZL1* | 9019 | myelin protein zero-like 1 | pSILAC |
| hsa-miR-155 | *MSH2* | 4436 | mutS homolog 2, colon cancer, nonpolyposis type 1 (E.coli) | Luciferase reporter assay//Western blot//Northern blot |
| hsa-miR-155 | *MSH6* | 2956 | mutS homolog 6 (E.coli) | Luciferase reporter assay//Western blot//Northern blot |
| hsa-miR-155 | *MSI2* | 124540 | musashi RNA binding protein 2 | pSILAC |
| hsa-miR-155 | *MYB* | 4602 | v-myb myeloblastosis viral oncogene homolog (avian) | Immunohistochemistry//Luciferase reporter assay//Northern blot//qRT-PCR//Western blot |
| hsa-miR-155 | *MYO10* | 4651 | myosin X | Luciferase reporter assay//qRT-PCR//Western blot |
| hsa-miR-155 | *MYO1E* | 4643 | myosin IE | pSILAC |
| hsa-miR-155 | *NAMPT* | 10135 | nicotinamide phosphoribosyltransferase | Microarray |
| hsa-miR-155 | *NARS* | 4677 | asparaginyl-tRNA synthetase | pSILAC |
| hsa-miR-155 | *NFATC2IP* | 84901 | nuclear factor of activated T-cella, cytoplasmic, calcineurin-dependent 2 interacting protein | Luciferase reporter assay |
| hsa-miR-155 | *NT5E* | 4907 | 5'nucleatidase, ecto (CD73) | pSILAC |
| hsa-miR-155 | *PAPOLA* | 10914 | poly(A) polymerase alpha | Microarray |
| hsa-miR-155 | *PDE3A* | 5139 | phosphodiesterase 3A,cGMP-inhibited | pSILAC |
| hsa-miR-155 | *PDLIM5* | 10611 | PDZ and LIM domain 5 | pSILAC |
| hsa-miR-155 | *PELI1* | 57162 | pellino E3 ubiquitin protein ligase 1 | Microarray |
| hsa-miR-155 | *PHC2* | 1912 | polyhomeotic homolog 2 (Drosophila) | pSILAC |
| hsa-miR-155 | *PHF17* | 79960 | PHD finger protein 17 | Luciferase reporter assay |
| hsa-miR-155 | *PICALM* | 8301 | phosphatidylinositol binding clathrin assembly protein | pSILAC |
| hsa-miR-155 | *PKN2* | 5586 | protein kinase N2 | pSILAC |
| hsa-miR-155 | *PLXND1* | 23129 | plexin D1 | pSILAC |
| hsa-miR-155 | *PODXL* | 5420 | podocalyxin-like | pSILAC |
| hsa-miR-155 | *POLE3* | 54107 | polymerase (DNA directed), epsilon 3, accessory subunit | pSILAC |
| hsa-miR-155 | *POLE4* | 56655 | polymerase (DNA-directed), episilon 4, acessory subunit | pSILAC |
| hsa-miR-155 | *PPL* | 5493 | periplakin | pSILAC |
| hsa-miR-155 | *PPP5C* | 5536 | protein phosphatase 5, catalytic subunit | pSILAC |
| hsa-miR-155 | *PRAF2* | 11230 | PRA1 domain family, member 2 | pSILAC |
| hsa-miR-155 | *PRKCI* | 5584 | protein kinase C, iota | pSILAC |
| hsa-miR-155 | *PSMG1* | 8624 | proteasome (prosome, macropain) assembly chaperone 1 | pSILAC |
| hsa-miR-155 | *PTPRJ* | 5795 | protein tyrosine phosphatase, receptor type, J | pSILAC |
| hsa-miR-155 | *RAB23* | 51715 | RAB23, member RAS oncogene family | pSILAC |
| hsa-miR-155 | *RAB27B* | 5874 | RAB27B, mamber RAS oncogene family | pSILAC |
| hsa-miR-155 | *RAB34* | 83871 | RAB34, member RAS oncogene family | pSILAC |
| hsa-miR-155 | *RAB5C* | 5878 | RAB5C, member RAS oncogene family | pSILAC |
| hsa-miR-155 | *RAB6A* | 5870 | RAB6A, member RAS oncogene family | pSILAC |
| hsa-miR-155 | *RAI14* | 26064 | retinoic acid induced 14 | pSILAC |
| hsa-miR-155 | *RCN2* | 5955 | reticulocalbin 2, EF-hand calcium binding domain | pSILAC |
| hsa-miR-155 | *RCOR1* | 23186 | REST corepressor 1 | pSILAC |
| hsa-miR-155 | *RHEB* | 6009 | Ras homolog enriched in brain | pSILAC |
| hsa-miR-155 | *RHOA* | 387 | ras homolog family member A | Luciferase reporter assay |
| hsa-miR-155 | *RUNX2* | 860 | runt-related transcription factor 2 | Luciferase reporter assay//qRT-PCR//Western blot |
| hsa-miR-155 | *SACM1L* | 22908 | SAC1 suppressor of actin mutations 1-like (yeast) | pSILAC |
| hsa-miR-155 | *SCAMP1* | 9522 | secretory carrier membrane protein 1 | pSILAC |
| hsa-miR-155 | *SDCBP* | 6386 | syndecan binding protein (syntenin) | pSILAC |
| hsa-miR-155 | *SELE* | 6401 | selectin E | Luciferase reporter assay |
| hsa-miR-155 | *SERTAD2* | 9792 | SERTA domain containing 2 | Microarray |
| hsa-miR-155 | *SH3BP4* | 23677 | SH3 domain binding protein 4 | pSILAC |
| hsa-miR-155 | *SKI* | 6497 | v-ski sarcoma viral oncogen homolog (avian) | Immunohistochemistry//Luciferase reporter assay//Northern blot//qRT-PCR//Western blot |
| hsa-miR-155 | *SLC25A40* | 55972 | solute carrier family 25, member 40 | pSILAC |
| hsa-miR-155 | *SLC30A1* | 7779 | solute carrier family 30(zinc transporter), member 1 | pSILAC |
| hsa-miR-155 | *SLC39A10* | 57181 | solute carrrier family 39 (zinc transporter), member 10 | Microarray |
| hsa-miR-155 | *SMAD1* | 4086 | SMAD family member 1 | Luciferase reporter assay//qRT-PCR//Western blot |
| hsa-miR-155 | *SMAD2* | 4087 | SMAD family member 2 | Luciferase reporter assay//qRT-PCR//Western blot |
| hsa-miR-155 | *SMAD5* | 4090 | SMAD family member 5 | Luciferase reporter assay |
| hsa-miR-155 | *SNAP29* | 9342 | synaptosomal associated protein, 29kDa | pSILAC |
| hsa-miR-155 | *SOCS1* | 8651 | suppressor of cytokine signaling 1 | ELISA//Luciferase reporter assay//qRT-PCR//Western blot |
| hsa-miR-155 | *SPI1* | 6688 | spleen focus forming viru (SFFV) proviral integration oncogene spi1 | Luciferase reporter assay |
| hsa-miR-155 | *SYNE2* | 23224 | spectrin repeat containing, nuclear envelope 2 | pSILAC |
| hsa-miR-155 | *SYPL1* | 6856 | synaptophysin-like 1 | pSILAC |
| hsa-miR-155 | *TAB2* | 23118 | TGF-beta activated kinase 1/MAP3K7 binding protein 2 | Luciferase reporter assay//Microarray//qRT-PCR//Western blot |
| hsa-miR-155 | *TACSTD2* | 4070 | tumor-associated calcium signal transducer 2 | pSILAC |
| hsa-miR-155 | *TBCA* | 6902 | tubulin folding cofactor A | pSILAC |
| hsa-miR-155 | *TLE4* | 7091 | transducin-like enhancer of split 4 (E(sp1) homolog, Drosophila) | Microarray |
| hsa-miR-155 | *TM6SF1* | 53346 | transmembrane 6 superfamily member 1 | Luciferase reporter assay |
| hsa-miR-155 | *TNFRSF10A* | 8797 | tumor necrosis factor receptor superfamily, member 10a | pSILAC |
| hsa-miR-155 | *TP53INP1* | 94241 | tumor protein p53 inducible nuclear protein 1 | Western blot//Luciferase reporter assay |
| hsa-miR-155 | *TRAM1* | 23471 | translocation associated membrane protein 1 | pSILAC |
| hsa-miR-155 | *TRIM32* | 22954 | tripartite motif containing 32 | pSILAC |
| hsa-miR-155 | *TRIP13* | 9319 | thyroid hormone receptor interactor 13 | pSILAC |
| hsa-miR-155 | *TSHZ3* | 57616 | teashirt zinc finger homebox 3 | Luciferase reporter assay |
| hsa-miR-155 | *TWF1* | 5756 | twinfilin actin-binding protein 1 | pSILAC |
| hsa-miR-155 | *TXNDC12* | 51060 | thioredoxin domain containing 12 (endoplasmic reticulum) | pSILAC |
| hsa-miR-155 | *TXNRD1* | 7296 | thioredoxin reductase 1 | pSILAC |
| hsa-miR-155 | *UBE2J1* | 51465 | ubiquitin-conjugating enzyme E2, J1 | pSILAC |
| hsa-miR-155 | *VAMP3* | 9341 | vesicle-associated membrane protein 3 | pSILAC |
| hsa-miR-155 | *VEZF1* | 7716 | vascular endothelial zinc finger 1 | pSILAC |
| hsa-miR-155 | *WDFY1* | 57590 | WD repeat and FYVE domain containing 1 | pSILAC |
| hsa-miR-155 | *ZIC3* | 7547 | Zic family member 3 | Luciferase reporter assay |
| hsa-miR-155 | *ZNF236* | 7776 | Zinc finger protein 236 | Microarray |
| hsa-miR-155 | *ZNF652* | 22834 | Zinc finger protein 652 | Luciferase reporter assay |
| hsa-miR-155 | *CKAP5* | 9793 | cytoskeleton associated protein 5 | Luciferase reporter assay//qRT-PCR//Western blot |
| hsa-miR-155 | *CSF1R* | 1436 | colony stimulating factor 1 receptor | ELISA//Luciferase reporter assay//qRT-PCR//Western blot |
| hsa-miR-155 | *CSNK1A1* | 1452 | casein kinase 1, alpha 1 | Luciferase reporter assay |
| hsa-miR-155 | *ETS1* | 2113 | v-ets erythroblastosis virus E26 oncogene homolog 1 (avian) | Luciferase reporter assay//Northern blot//qRT-PCR//Western blot |
| hsa-miR-155 | *GCSAM* | 257144 | germinal center-associated, signaling and motility | Luciferase reporter assay//Western blot |
| hsa-miR-155 | *JUN* | 3725 | jun proto-oncogene | Luciferase reporter assay//qRT-PCR//Western blot |
| hsa-miR-155 | *KDM3A* | 55818 | lysine (K)-specific demethylase 3A | In situ hybridization//Luciferase reporter assay//qRT-PCR//Western blot |
| hsa-miR-155 | *PKIA* | 5569 | protein kinase (cAMP-dependent, catalytic) inhibitor alpha | ChIP-seq//Immunoblot//Luciferase reporter assay//qRT-PCR |
| hsa-miR-155 | *RNF123* | 63891 | ring finger protein 123 | ELISA//Luciferase reporter assay//qRT-PCR//Western blot |
| hsa-miR-155 | *SOX6* | 55553 | SRY (sex determining region Y)-box 6 | Luciferase reporter assay//qRT-PCR//Western blot |
| hsa-miR-155 | *UQCRFS1* | 7386 | ubiquinol-cytochrome c reductase, Rieske iron-sulfur polypeptide 1 | Luciferase reporter assay//Western blot |
| hsa-miR-188 | *UBE2I* | 7329 | ubiquitin-conjugating enzyme E2I | qRT-PCR//Western blot |
| hsa-miR-197 | *ABCC3* | 8714 | ATP-binding cassette, sub-family C (CFTR/MRP), member 3 | Microarray |
| hsa-miR-197 | *ACVR1* | 90 | activin A receptor, type I | Microarray//qRT-PCR |
| hsa-miR-197 | *AGR2* | 10551 | anterior gradient 2 homolog (xenopus laevis) | Microarray |
| hsa-miR-197 | *ALMS1* | 7840 | Alstorm syndrome 1 | Microarray |
| hsa-miR-197 | *ATF6B* | 1388 | activating transcription factor 6 beta | Microarray |
| hsa-miR-197 | *C1orf38* | 9473 | thymocyte selection associated family member 2 | Microarray |
| hsa-miR-197 | *CDK10* | 8558 | cyclin-dependent kinase 10 | Microarray |
| hsa-miR-197 | *CES1* | 1066 | carboxylesterase 1 | Microarray |
| hsa-miR-197 | *CHIC2* | 26511 | cysteine-rich hydrophobic domain 2 | Microarray |
| hsa-miR-197 | *CLIC1* | 1192 | chloride intracellular channel 1 | Microarray |
| hsa-miR-197 | *CPNE6* | 9362 | copine VI (neuronal) | Microarray |
| hsa-miR-197 | *CPSF1* | 29894 | cleavage and polyadenylation specific factor 1, 160kDa | Microarray |
| hsa-miR-197 | *CYLD* | 1540 | cylindromatosis (turban tumor syndrome) | Microarray |
| hsa-miR-197 | *DCBLD2* | 131566 | discoidin, CUB and LCCL domain containing 2 | Microarray |
| hsa-miR-197 | *DPH1* | 1801 | diphthamide biosynthesis 1 | Microarray |
| hsa-miR-197 | *DPYSL3* | 1809 | dihydropyrimidinase-like 3 | Microarray |
| hsa-miR-197 | *EHD2* | 30846 | EH-domain containing 2 | Microarray |
| hsa-miR-197 | *FBXW7* | 55294 | F-box and WD repeat domain containing 7, E3 ubiquitin protein ligase | Microarray |
| hsa-miR-197 | *FOXO3* | 2309 | forkhead box O3 | Microarray |
| hsa-miR-197 | *FUS* | 2521 | fused in sarcoma | Microarray |
| hsa-miR-197 | *GALT* | 2592 | galactose-1-phophate uridylyltransferase | Microarray |
| hsa-miR-197 | *GOLGB1* | 2804 | golgin B1 | Microarray |
| hsa-miR-197 | *GORASP2* | 26003 | golgi reassembly stacking protein 2, 55kDa | Microarray |
| hsa-miR-197 | *HMGN1* | 3150 | high mobility group nucleasome binding domain 1 | Microarray |
| hsa-miR-197 | *HNF4A* | 3172 | hepatocyte nuclear factor 4, alpha | Microarray |
| hsa-miR-197 | *HNRNPD* | 3184 | heterogenous nuclear ribonucleoprotein D (AU-rich element RNA binding protein 1, 37kDa) | Microarray |
| hsa-miR-197 | *HPN* | 3249 | hepsin | Microarray |
| hsa-miR-197 | *IER3* | 8870 | immediate early response 3 | Microarray |
| hsa-miR-197 | *IGF2AS* | 51214 | IGF2 antisense RNA | Microarray |
| hsa-miR-197 | *IL1R1* | 3554 | interleukin 1 receptor, type I | Microarray |
| hsa-miR-197 | *ISYNA1* | 51477 | inositol-3-phosphatase synthase 1 | Microarray |
| hsa-miR-197 | *KLC1* | 3831 | kinesin light chain 1 | Microarray |
| hsa-miR-197 | *KLF10* | 7071 | Kruppel-like factor 10 | Microarray |
| hsa-miR-197 | *LRP4* | 4038 | low density lipoprotein receptor-related protein 4 | Microarray |
| hsa-miR-197 | *MED16* | 10025 | mediator complex subunit 16 | Microarray |
| hsa-miR-197 | *MMP23A* | 8511 | matrix metallopeptidase 23A (pseudogene) | Microarray |
| hsa-miR-197 | *NEK4* | 6787 | NIMA-related kinase 4 | Microarray |
| hsa-miR-197 | *PEX13* | 5194 | peroxisomal biogenesis factor 13 | Microarray |
| hsa-miR-197 | *PHF20* | 51230 | PHD finger protein 20 | Microarray |
| hsa-miR-197 | *PIPOX* | 51268 | pipecolic acid oxidase | Microarray |
| hsa-miR-197 | *PRKAR2A* | 5576 | protein kinase, cAMP-dependent, regulatory, type II, alpha | Microarray |
| hsa-miR-197 | *PRKD2* | 25865 | protein kinase D2 | Microarray |
| hsa-miR-197 | *RAB28* | 9364 | RAB28, member RAS oncogene family | Microarray |
| hsa-miR-197 | *RAD51* | 5888 | RAD51 homolog (S.cervisiae) | Microarray |
| hsa-miR-197 | *RBM4* | 5936 | RNA binding motif protein 4 | Microarray |
| hsa-miR-197 | *RFX1* | 5989 | regulatory factor X, 1 (influences HLA class II expression) | Microarray |
| hsa-miR-197 | *RXRB* | 6257 | retinoid X receptor, beta | Microarray |
| hsa-miR-197 | *SNX1* | 6642 | sorting nexin 1 | Microarray |
| hsa-miR-197 | *TAF4B* | 6875 | TAF4b RNA polymerase II, TATA box protein (TBP)-associated factor, 105kDa | Microarray |
| hsa-miR-197 | *TSPAN3* | 10099 | tetraspanin 3 | Microarray//qRT-PCR |
| hsa-miR-197 | *TSPYL1* | 7259 | TSPY-like 1 | Microarray |
| hsa-miR-197 | *TUSC2* | 11334 | tumor suppressor cancdidate 2 | Luciferase reporter assay//Western blot |
| hsa-miR-197 | *UMPS* | 7372 | uridine monophosphate synthetase | Microarray |
| hsa-miR-197 | *USO1* | 8615 | USO1 vesicle transport factor | Microarray |
| hsa-miR-197 | *WDR6* | 11180 | WD epeat domain 6 | Microarray |
| hsa-miR-197 | *ZNF175* | 7728 | zinc finger protein 175 | Microarray |
| hsa-miR-197 | *ZNF208* | 7757 | zinc finger protein 208 | Microarray |
| hsa-miR-197 | *ZNF302* | 55900 | zinc finger protein 302 | Microarray |
| hsa-miR-218 | *ACTN1* | 87 | actinin, alpha 1 | Luciferase reporter assay//qRT-PCR |
| hsa-miR-218 | *BIRC6* | 57448 | baculoviral IAP repeat containing 6 | Luciferase reporter assay//qRT-PCR |
| hsa-miR-218 | *CDKN1B* | 1027 | cyclin-dependent kinase inhibitor 1B (p27, Kip1) | Luciferase reporter assay |
| hsa-miR-218 | *EBP* | 10682 | emopamil binding protein (sterol isomerase) | qRT-PCR//Western blot |
| hsa-miR-218 | *EFNA1* | 1942 | ephrin-A1 | qRT-PCR//Western blot |
| hsa-miR-218 | *IKBKB* | 3551 | inhibitor of kappa light polypeptide gene enhancer in B-cells, kinase beta | Luciferase reporter assay//qRT-PCR//Western blot |
| hsa-miR-218 | *LAMB3* | 3914 | laminin, beta 3 | qRT-PCR//Western blot//Northern blot |
| hsa-miR-218 | *LASP1* | 3927 | LIM and SH3 protein 1 | qRT-PCR |
| hsa-miR-218 | *MAFG* | 4097 | v-maf musculoaponeurotic fibrosarcoma oncogene homolog G (avian) | qRT-PCR |
| hsa-miR-218 | *MBNL2* | 10150 | muscleblind-like splicing regulator 2 | qRT-PCR//Western blot |
| hsa-miR-218 | *MRPS27* | 23107 | mitochondrial ribosomal protein S27 | qRT-PCR//Western blot |
| hsa-miR-218 | *NACC1* | 112939 | nucleus accumbens associated 1, BEN and BTB (POZ) domain containing | qRT-PCR |
| hsa-miR-218 | *NFE2L1* | 4779 | nuclear factor (erythroid-derived)-like 1 | qRT-PCR |
| hsa-miR-218 | *NUP93* | 9688 | nucleoporin 93kDa | qRT-PCR//Western blot |
| hsa-miR-218 | *SP1* | 6667 | Sp1 transcription factor | Immunoblot//Luciferase reporter assay//qRT-PCR |
| hsa-miR-218 | *STAM2* | 10254 | signal transducing adaptor molecule (SH3 domain and ITAM motif) 2 | Luciferase reporter assay//qRT-PCR |
| hsa-miR-218 | *VOPP1* | 81552 | vesicular, overexpressed in cancer, prosurvival protein 1 | qRT-PCR |
| hsa-miR-218 | *BIRC5* | 332 | baculoviral IAP repeat containing 5 | Immunoblot//Luciferase reporter assay//Microarray//qRT-PCR |
| hsa-miR-218 | *GJA1* | 2697 | gap junction protein, alpha 1, 43kDa | Immunoblot//Luciferase reporter assay//Microarray//qRT-PCR |
| hsa-miR-218 | *RICTOR* | 253260 | RPTOR independent companion of MTOR, complex 2 | Luciferase reporter assay//qRT-PCR//Western blot |
| hsa-miR-218 | *ROBO1* | 6091 | roundabout, axon guidance receptor, homolog 1 (Drosophila) | Immunoblot//Luciferase reporter assay//Microarray//qRT-PCR |
| hsa-miR-29c | *BCL2* | 596 | B-cell CLL/lymphoma 2 | Luciferase reporter assay//qRT-PCR//Western blot |
| hsa-miR-29c | *CDK6* | 1021 | cyclin dependent kinase 6 | Luciferase reporter assay//qRT-PCR//Western blot |
| hsa-miR-29c | *COL15A1* | 1306 | collagen, type XV, alpha 1 | Luciferase reporter assay//qRT-PCR |
| hsa-miR-29c | *COL1A1* | 1277 | collagen, type I, alpha 1 | Luciferase reporter assay//Immunohistochemistry//qRT-PCR |
| hsa-miR-29c | *COL1A2* | 1278 | collagen, type I, alpha 2 | Luciferase reporter assay//Immunohistochemistry//qRT-PCR |
| hsa-miR-29c | *COL3A1* | 1281 | collagen, type III, alpha 1 | Luciferase reporter assay |
| hsa-miR-29c | *COL4A1* | 1282 | collagen, type IV, alpha 1 | Luciferase reporter assay |
| hsa-miR-29c | *COL4A2* | 1284 | collagen, type IV, alpha 2 | Luciferase reporter assay |
| hsa-miR-29c | *DNMT3A* | 1788 | DNA (cytosine-5-)-methyltransferase 3 alpha | Luciferase reporter assay//Western blot//qRT-PCR |
| hsa-miR-29c | *DNMT3B* | 1789 | DNA (cytosine-5-)-methyltransferase 3 beta | Luciferase reporter assay//Western blot//qRT-PCR |
| hsa-miR-29c | *FBN1* | 2200 | fibrillin 1 | Luciferase reporter assay//qRT-PCR |
| hsa-miR-29c | *FGA* | 2243 | fibrinogen alpha chain | Luciferase reporter assay |
| hsa-miR-29c | *FGB* | 2244 | fibrinogen beta chain | Luciferase reporter assay |
| hsa-miR-29c | *FGG* | 2266 | fibrinogen gamma chain | Luciferase reporter assay |
| hsa-miR-29c | *GAPDH* | 2597 | glyceraldehyde-3-phosphate dehydrogenase | Luciferase reporter assay |
| hsa-miR-29c | *LAMC1* | 3915 | laminin, gamma 1 (formely LAMB2) | Luciferase reporter assay |
| hsa-miR-29c | *MCL1* | 4170 | myeloid cell leukemia sequence 1 (BCL2-related) | Luciferase reporter assay//qRT-PCR//Western blot |
| hsa-miR-29c | *MMP15* | 4324 | matrix metallopeptidase 15 (membrane-inserted) | Luciferase reporter assay//qRT-PCR |
| hsa-miR-29c | *MMP24* | 10893 | matrix metallopeptidase 24 (membrane-inserted) | Luciferase reporter assay//qRT-PCR |
| hsa-miR-29c | *SPARC* | 6678 | secreted protein, acidic, cysteine-rich (osteonectin) | Luciferase reporter assay//Immunohistochemistry//qRT-PCR |
| hsa-miR-29c | *SRSF10* | 10772 | serine/arginine-rich splicing factor 10 | Luciferase reporter assay |
| hsa-miR-29c | *TDG* | 6996 | thymine-DNA glycosylase | Luciferase reporter assay |
| hsa-miR-29c | *CDC42* | 998 | cell division cycle 42 | Luciferase reporter assay//Western blot |
| hsa-miR-29c | *COL21A1* | 81578 | collagen, type XXI, alpha 1 | Luciferase reporter assay |
| hsa-miR-29c | *COL7A1* | 1294 | collagen, type VII, alpha 1 | Luciferase reporter assay |
| hsa-miR-29c | *IGFBP1* | 3484 | insulin-like growth factor binding protein 1 | Luciferase reporter assay |
| hsa-miR-29c | *PPP1R13B* | 23368 | protein phosphatase 1, regulatory subunit 13B | Luciferase reporter assay//Western blot |
| hsa-miR-29c | *TFAP2C* | 7022 | transcription factor AP-2 gamma (activating enhancer binding protein 2 gamma) | Luciferase reporter assay |
| hsa-miR-29c | *WNT4* | 54361 | wingless-related MMTV integration site 4 | Luciferase reporter assay |
| hsa-miR-30c | *MUC17* | 140453 | mucin 17, cell surface associated | Immunohistochemistry//Microarray//qRT-PCR |
| hsa-miR-30c | *SERPINE1* | 5054 | serpin peptidase inhibitor, clade E (nexin, plasminogen activator inhibitor type 1), member 1 | Luciferase reporter assay |
| hsa-miR-30c | *UBE2I* | 7329 | ubiquitin-conjugating enzyme E2I | Luciferase reporter assay//qRT-PCR//Western blot |
| hsa-miR-30c | *SMAD1* | 4086 | SMAD family member 1 | Luciferase reporter assay//Western blot |
| hsa-miR-30d | *GNAI2* | 2771 | guanine nucleotide binding protein (G protein), alpha inhibiting activity polypeptide 2 | Western blot |
| hsa-miR-30d | *TP53* | 7157 | tumor protein p53 | Luciferase reporter assay//qRT-PCR//Western blot |
| hsa-miR-30d | *CASP3* | 836 | caspase 3, apoptosis-related cysteine peptidase | Luciferase reporter assay//Western blot |
| hsa-miR-30d | *SMAD1* | 4086 | SMAD family member 1 | Luciferase reporter assay//Western blot |
| hsa-miR-34b | *CDK4* | 1019 | cyclin-dependent kinase 4 | Microarray//Western blot//RTPCR |
| hsa-miR-34b | *CDK6* | 1021 | cyclin dependent kinase 6 | Luciferase reporter assay//Western blot |
| hsa-miR-34b | *MET* | 4233 | met proto-oncogene (hepatocyte growth factor receptor) | Microarray//Western blot//RTPCR |
| hsa-miR-34b | *MYC* | 4609 | v-myc myelocytomatosis viral oncogene homolog (avian) | Luciferase reporter assay//Western blot |
| hsa-miR-34b | *VEGFA* | 7422 | vascular endothelial growth factor A | ELISA//Luciferase reporter assay |
| hsa-miR-34b | *ZAP70* | 7535 | zeta-chain (TCR) associated protein kinase 70kDa | Immunoblot//Luciferase reporter assay |
| hsa-miR-370 | *CPT1A* | 1374 | carnitine palmitoyltransferase 1A (liver) | Luciferase reporter assay//qRT-PCR//Western blot |
| hsa-miR-370 | *HMGA2* | 8091 | high mobility group AT-hook 2 | Luciferase reporter assay |
| hsa-miR-370 | *MAP3K8* | 1326 | mitogen-activated protein kinase kinase kinase 8 | Luciferase reporter assay//Western blot |
| hsa-miR-375 | *ELAVL4* | 1996 | ELAV (embryonic lethal, abnormal vision, Drosophila)-like 4 | GFP reporter assay//Immunohistochemistry//Microarray//qRT-PCR//Western blot |
| hsa-miR-375 | *JAK2* | 3717 | Janus kinase 2 | Luciferase reporter assay//Western blot |
| hsa-miR-375 | *MTDH* | 92140 | metadherin | Immunofluorescence//Luciferase reporter assay//qRT-PCR//Western blot |
| hsa-miR-375 | *PDK1* | 5163 | pyruvate dehydrogenase kinase, isozyme 1 | Immunohistochemistry//Luciferase reporter assay//qRT-PCR//Western blot |
| hsa-miR-375 | *PLAG1* | 5324 | pleiomorphic adenoma gene 1 | Luciferase reporter assay |
| hsa-miR-375 | *RASD1* | 51655 | RAS, dexamethasone-induced 1 | Luciferase reporter assay//Microarray//qRT-PCR |
| hsa-miR-375 | *TIMM8A* | 1678 | translocase of inner mitochondrial membrane 8 homolog A (yeast) | Luciferase reporter assay |
| hsa-miR-375 | *YAP1* | 10413 | Yes-associated protein 1 | Luciferase reporter assay//Microarray//qRT-PCR |
| hsa-miR-375 | *YY1AP1* | 55249 | YY1 associated protein 1 | Luciferase reporter assay//Western blot |
| hsa-miR-378 | *GALNT7* | 51809 | UDP-N-acetyl-alpha-D-galactosamine:polypeptide N-acetygalactosaminyltransferase 7 (GaINAc-T7) | GFP reporter assay//Luciferase reporter assay//Western blot |
| hsa-miR-378 | *MYC* | 4609 | v-myc myelocytomatosis viral oncogene homolog (avian) | Immunoblot//Luciferase reporter assay//qRT-PCR |
| hsa-miR-378 | *NPNT* | 255743 | nephronectin | GFP reporter assay//Luciferase reporter assay//Western blot |
| hsa-miR-378 | *TOB2* | 10766 | transducer of ERBB2, 2 | Immunoblot//Luciferase reporter assay//qRT-PCR |
| hsa-miR-378 | *VEGFA* | 7422 | vascular endothelial growth factor A | ELISA//Luciferase reporter assay |
| hsa-miR-433 | *FGF20* | 26281 | fibroblast growth factor 20 | Luciferase reporter assay//Western blot |
| hsa-miR-433 | *GRB2* | 2885 | growth factor receptor-bound protein 2 | Luciferase reporter assay//Western blot |
| hsa-miR-489 | *PTPN11* | 5781 | protein tyrosine phosphatase, non-receptor type 11 | Luciferase reporter assay//Western blot |
| hsa-miR-494 | *CDK6* | 1021 | cyclin dependent kinase 6 | Luciferase reporter assay//Microarray//qRT-PCR//Western blot |
| hsa-miR-494 | *PTEN* | 5728 | phosphatase and tensin homolog | qRT-PCR//Luciferase reporter assay//Western blot |
| hsa-miR-513a-5p | *CD274* | 29126 | CD274 molecule | Luciferase reporter assay//Western blot |

List of experimentally validated targets from miRTarBase for miRNAs downregulated in gastric cancer. Total 378 redundant target genes with 362 non-redundant target genes are listed with MicroRNA, Target gene, Entrez gene ID, Gene name and Validation method

Abbreviation: ChiP: chromatin immunoprecipitation, ELISA: enzyme-linked immunosorbent assay, GFP: green fluorescent protein, pSILAC: pulsed stable isotope labeling with amino acids in culture, qRT-PCR: quantitative RT-PCR

**Supplementary Table S3.** Experimentally validated target genes for microRNAs up-regulated in gastric cancer

| MicroRNA | Target gene | Entrez gene ID | Gene name | Validation method |
| --- | --- | --- | --- | --- |
| hsa-let-7i | *TLR4* | 7099 | toll-like receptor 4 | Luciferase reporter assay//qRT-PCR//Western blot |
| hsa-let-7i | *IL13* | 3596 | interleukin 13 | Luciferase reporter assay |
| hsa-let-7i | *SOCS1* | 8651 | SOCS1 | Luciferase reporter assay |
| hsa-miR-100 | *ATM* | 472 | ataxia telangiectasia mutated | Luciferase reporter assay//qRT-PCR//Western blot |
| hsa-miR-100 | *EGR2* | 1959 | early growth response 2 | Microarray//qRT-PCR |
| hsa-miR-100 | *FGFR3* | 2261 | fibroblast growth factor receptor 3 | Luciferase reporter assay//qRT-PCR//Western blot |
| hsa-miR-100 | *ID1* | 3397 | inhibitor of DNA binding 1, dominant negative helix-loop-helix protein | Microarray//qRT-PCR |
| hsa-miR-100 | *MMP13* | 4322 | matrix metallopeptidase 13 (collagenase) | Microarray//qRT-PCR//Western blot |
| hsa-miR-100 | *PLK1* | 5347 | polo-like kinase 1 | Luciferase reporter assay//qRT-PCR//Western blot |
| hsa-miR-100 | *IGF1R* | 3480 | insulin-like growth factor receptor | Luciferase reporter assay |
| hsa-miR-106a | *APP* | 351 | amyloid beta (A4) precursor protein | Luciferase reporter assay |
| hsa-miR-106a | *ARID4B* | 51742 | AT rich interactive domain 4B (RBP1-like) | Luciferase reporter assay |
| hsa-miR-106a | *CDKN1A* | 1026 | cyclin-dependent kinase inhibitor 1A (p21, Cip1) | qRT-PCR//Luciferase reporter assay//Western blot |
| hsa-miR-106a | *E2F1* | 1869 | E2F transcription factor 1 | Immunohistochemistry//Luciferase reporter assay//Microarray//Western blot |
| hsa-miR-106a | *HIPK3* | 10114 | homeodomain interacting protein kinase 3 | Luciferase reporter assay |
| hsa-miR-106a | *IL10* | 3586 | interleukin 10 | Luciferase reporter assay//qRT-PCR |
| hsa-miR-106a | *MYLIP* | 29116 | myosin regulatory light chain interacting protein | Luciferase reporter assay |
| hsa-miR-106a | *RB1* | 5925 | retinoblastoma 1 | Microarray |
| hsa-miR-106a | *RUNX1* | 861 | runt-related transcription factor 1 | Luciferase reporter assay//qRT-PCR//Western blot |
| hsa-miR-106a | *VEGFA* | 7422 | vascular endothelial growth factor A | ELISA//Luciferase reporter assay |
| hsa-miR-106a | *FAS* | 355 | Fas cell surface death receptor | Luciferase reporter assay |
| hsa-miR-106b | *APP* | 351 | amyloid beta (A4) precursor protein | Luciferase reporter assay//Western blot |
| hsa-miR-106b | *CCND1* | 595 | cyclin D1 | Luciferase reporter assay |
| hsa-miR-106b | *CCND2* | 894 | cyclin D2 | Luciferase reporter assay |
| hsa-miR-106b | *CDKN1A* | 1026 | cyclin-dependent kinase inhibitor 1A (p21, Cip1) | Luciferase reporter assay |
| hsa-miR-106b | *E2F1* | 1869 | E2F transcription factor 1 | Luciferase reporter assay |
| hsa-miR-106b | *E2F3* | 1871 | E2F transcription factor 3 | Luciferase reporter assay |
| hsa-miR-106b | *ITCH* | 83737 | itchy E3 ubiquitin protein ligase | Luciferase reporter assay |
| hsa-miR-106b | *KAT2B* | 8850 | K(lysine) acetyltransferase 2B | Western blot//Luciferase reporter assay |
| hsa-miR-106b | *MAPK9* | 5601 | mitogen-activated protein kinase 9 | Luciferase reporter assay |
| hsa-miR-106b | *PTEN* | 5728 | phosphatase and tensin homolog | Luciferase reporter assay |
| hsa-miR-106b | *RB1* | 5925 | retinoblastoma 1 | immunohistochemistry//Microarray |
| hsa-miR-106b | *RBL1* | 5933 | retinoblastoma-like 1 (p107) | Luciferase reporter assay |
| hsa-miR-106b | *RBL2* | 5934 | retinoblastoma-like 2 (p130) | Luciferase reporter assay |
| hsa-miR-106b | *TCEAL1* | 9338 | transcription elongation factor A (SII)-like 1 | Luciferase reporter assay//qRT-PCR//Western blot |
| hsa-miR-106b | *VEGFA* | 7422 | vascular endothelial growth factor A | ELISA//Luciferase reporter assay |
| hsa-miR-106b | *WEE1* | 7465 | WEE1 homolog (S.pombe) | Luciferase reporter assay |
| hsa-miR-106b | *EOMES* | 8320 | eomesodermin | Luciferase reporter assay |
| hsa-miR-10a | *BTRC* | 8945 | beta-transducin repeat containing E3 ubiquitin protein ligase | Luciferase reporter assay//Microarray//qRT-PCR//Western blot |
| hsa-miR-10a | *HOXA1* | 3198 | homeobox A1 | Luciferase reporter assay//qRT-PCR//Western blot |
| hsa-miR-10a | *MAP3K7* | 6885 | mitogen-activated protein kinase kinase kinase 7 | Luciferase reporter assay//Microarray//qRT-PCR//Western blot |
| hsa-miR-10a | *NCOR2* | 9612 | nuclear receptor corepressor 2 | qRT-PCR//Western blot |
| hsa-miR-10a | *USF2* | 7392 | upstream transcription factor 2, c-fos interacting | Luciferase reporter assay//qRT-PCR//Western blot |
| hsa-miR-10a | *SRSF1* | 6426 | serine/arginine-rich splicing factor 1 | Luciferase reporter assay |
| hsa-miR-10a | *TRA2B* | 6434 | transformer 2 beta homolog (Drosophila) | Luciferase reporter assay |
| hsa-miR-135b | *APC* | 324 | adenomatous polyposis coli | Luciferase reporter assay//qRT-PCR |
| hsa-miR-135b | *BGLAP* | 632 | bone gamma-carboxyglutamate (gla) protein | qRT-PCR |
| hsa-miR-135b | *IBSP* | 3381 | integrin-binding sialoprotein | qRT-PCR |
| hsa-miR-135b | *KLF4* | 9314 | Kruppel-like factor 4 (gut) | Luciferase reporter assay//Microarray//qRT-PCR |
| hsa-miR-135b | *MAFB* | 9935 | v-maf musculoaponeurotic fibrosarcoma oncogene homolog B (avian) | Luciferase reporter assay//Microarray//qRT-PCR |
| hsa-miR-135b | *RUNX2* | 860 | runt-related transcription factor 2 | qRT-PCR |
| hsa-miR-135b | *SP7* | 121340 | Sp7 transcription factor | qRT-PCR |
| hsa-miR-15a | *ACTR1A* | 10121 | ARP1 actin-related protein 1 homolog A, centractin alpha (yeast) | proteomics analysis |
| hsa-miR-15a | *ANAPC16* | 119504 | anaphase promoting complex subunit 16 | Microarray |
| hsa-miR-15a | *APP* | 351 | amyloid beta (A4) precursor protein | Luciferase reporter assay |
| hsa-miR-15a | *ASXL2* | 55252 | additional sex combs like 2 (Drosophila) | Microarray |
| hsa-miR-15a | *BACE1* | 23621 | beta-site APP-cleaving enzyme 1 | Luciferase reporter assay |
| hsa-miR-15a | *BCL2* | 596 | B-cell CLL/lymphoma 2 | qRT-PCR |
| hsa-miR-15a | *BMI1* | 648 | BMI1 polycomb ring finger oncogene | Luciferase reporter assay//Western blot |
| hsa-miR-15a | *BRCA1* | 672 | breast cancer 1, early onset | Luciferase reporter assay |
| hsa-miR-15a | *C14orf109* | 26175 | transmembrane protein 251 | Microarray |
| hsa-miR-15a | *C17orf80* | 55028 | chromosome 17 open reading frame 80 | Microarray |
| hsa-miR-15a | *C2orf43* | 60526 | chromosome 2 open reading frame 43 | Microarray |
| hsa-miR-15a | *C2orf74* | 339804 | chromosome 2 open reading frame 74 | Microarray |
| hsa-miR-15a | *C4orf27* | 54969 | chromosome 4 open reading frame 27 | Microarray |
| hsa-miR-15a | *CADM1* | 23705 | cell adhesion molecule 1 | Reporter assay//qRT-PCR |
| hsa-miR-15a | *CARD8* | 22900 | caspase recruitment domain family, member 8 | Microarray |
| hsa-miR-15a | *CCDC111* | 201973 | coiled-coil domain containing 111 | Microarray |
| hsa-miR-15a | *CCDC76* | 54482 | tRNA methyltransferase 13 homolog (S. cerevisiae) | Microarray |
| hsa-miR-15a | *CCND1* | 595 | cyclin D1 | qRT-PCR//Luciferase reporter assay//Western blot |
| hsa-miR-15a | *CCND2* | 894 | cyclin D2 | Luciferase reporter assay |
| hsa-miR-15a | *CCNE1* | 898 | cyclin E1 | qRT-PCR//Luciferase reporter assay//Western blot |
| hsa-miR-15a | *CDC14B* | 8555 | cell division cycle 14B | Microarray |
| hsa-miR-15a | *CDC25A* | 993 | cell division cycle 25A | Luciferase reporter assay |
| hsa-miR-15a | *CENPJ* | 55835 | centromere protein J | Microarray |
| hsa-miR-15a | *CEP63* | 80254 | centrosomal protein 63kDa | Microarray |
| hsa-miR-15a | *CHUK* | 1147 | conserved helix-loop-helix ubiquitous kinase | Luciferase reporter assay//qRT-PCR//Western blot |
| hsa-miR-15a | *CREBL2* | 1389 | cAMP responsive element binding protein-like 2 | Microarray |
| hsa-miR-15a | *DMTF1* | 9988 | cyclin D binding myb-like transcription factor 1 | Luciferase reporter assay |
| hsa-miR-15a | *ECHDC1* | 55862 | enoyl CoA hydratase domain containing 1 | Microarray |
| hsa-miR-15a | *FAM122C* | 159091 | family with sequence similarity 122C | Microarray |
| hsa-miR-15a | *FAM69A* | 388650 | family with sequence similarity 69, member A | Microarray |
| hsa-miR-15a | *GOLGA5* | 9950 | golgin A5 | Microarray |
| hsa-miR-15a | *GOLPH3L* | 55204 | golgi phosphoprotein 3-like | Microarray |
| hsa-miR-15a | *GTF2H1* | 2965 | general transcription factor IIH, polypeptide 1, 62kDa | Microarray |
| hsa-miR-15a | *H3F3B* | 3021 | H3 histone, family 3B (H3.3B) | Microarray |
| hsa-miR-15a | *HACE1* | 57531 | HECT domain and ankyrin repeat containing E3 ubiquitin protein ligase 1 | Microarray |
| hsa-miR-15a | *HDHD2* | 84064 | haloacid dehalogenase-like hydrolase domain containing 2 | Microarray |
| hsa-miR-15a | *HERC6* | 55008 | HECT and RLD domain containing E3 ubiquitin protein ligase family member 6 | Microarray |
| hsa-miR-15a | *HRSP12* | 10247 | heat-responsive protein 12 | Microarray |
| hsa-miR-15a | *HSDL2* | 84263 | hydroxysteroid dehydrogenase like 2 | Microarray |
| hsa-miR-15a | *HSP90B1* | 7184 | heat shock protein 90kDa beta (Grp94), member 1 | Microarray |
| hsa-miR-15a | *HSPA1A* | 3303 | heat shock 70kDa protein 1A | Microarray |
| hsa-miR-15a | *JUN* | 3725 | jun proto-oncogene | Microarray |
| hsa-miR-15a | *MCL1* | 4170 | myeloid cell leukemia sequence 1 (BCL2-related) | Microarray |
| hsa-miR-15a | *MSH2* | 4436 | mutS homolog, colon cancer, nonpolyposis type 1 (E.coli) | Microarray |
| hsa-miR-15a | *MYB* | 4602 | v-myb myeloblastosis viral oncogene homolog (avian) | Luciferase reporter assay//Western blot |
| hsa-miR-15a | *NFKB1* | 4790 | nuclear factor of kappa light polypeptide gene enhancer in B-cells | Microarray//qRT-PCR |
| hsa-miR-15a | *NIPAL2* | 79815 | NIPA-like domain containing 2 | Microarray |
| hsa-miR-15a | *NT5DC1* | 221294 | 5'-nucleotidase domain containing 1 | Microarray |
| hsa-miR-15a | *OMA1* | 115209 | OMA1 zinc metallopeptidase | Microarray |
| hsa-miR-15a | *OSGEPL1* | 64172 | O-sialoglycoprotein endopeptidase-like 1 | Microarray |
| hsa-miR-15a | *PDCD4* | 27250 | programmed cell death 4 (neoplastic transformation inhibitor) | qRT-PCR |
| hsa-miR-15a | *PDCD6IP* | 10015 | programmed cell death 6 interacting protein | Microarray |
| hsa-miR-15a | *PHKB* | 5257 | phosphorylase kinase, beta | Microarray |
| hsa-miR-15a | *PMS1* | 5378 | PMS1 postmeiotic segregation increased 1 (S. cerevisiae) | Microarray |
| hsa-miR-15a | *PNN* | 5411 | pinin, desmosome associated protein | Microarray |
| hsa-miR-15a | *PRIM1* | 5557 | primase, DNA, polypeptide 1 (49kDa) | Microarray |
| hsa-miR-15a | *PWWP2A* | 114825 | PWWP domain containing 2A | Microarray |
| hsa-miR-15a | *RAB21* | 23011 | RAB21, member RAS oncogene family | qRT-PCR |
| hsa-miR-15a | *RAB9B* | 51209 | RAB9B, member RAS oncogene family | proteomics analysis |
| hsa-miR-15a | *RAD51C* | 5889 | RAD51 homolog C (S. cerevisiae) | Microarray |
| hsa-miR-15a | *RHOT1* | 55288 | ras homolog family member T1 | Microarray |
| hsa-miR-15a | *RNASEL* | 6041 | ribonuclease L (2',5'-oligoisoadenylate synthetase-dependent) | Microarray |
| hsa-miR-15a | *SKAP2* | 8935 | src kinase associated phosphoprotein 2 | Microarray//qRT-PCR |
| hsa-miR-15a | *SLC35A1* | 10559 | solute carrier family 35 (CMP-sialic acid transporter), member A1 | Microarray |
| hsa-miR-15a | *SLC35B3* | 51000 | solute carrier family 35, member B3 | Microarray |
| hsa-miR-15a | *TIA1* | 7072 | TIA1 cytotoxic granule-associated RNA binding protein | Microarray |
| hsa-miR-15a | *TMEM184B* | 25829 | transmembrane protein 184B | Luciferase reporter assay |
| hsa-miR-15a | *TP53* | 7157 | tumor protein p53 | Immunoblot//Luciferase reporter assay |
| hsa-miR-15a | *TPI1* | 7167 | triosephosphate isomerase 1 | proteomics analysis |
| hsa-miR-15a | *TSPYL2* | 64061 | TSPY-like 2 | Luciferase reporter assay |
| hsa-miR-15a | *UCP2* | 7351 | uncoupling protein 2 (mitochondrial, proton carrier) | Luciferase reporter assay//qRT-PCR |
| hsa-miR-15a | *UGDH* | 7358 | UDP-glucose 6-dehydrogenase | Microarray |
| hsa-miR-15a | *UGP2* | 7360 | UDP-glucose pyrophosphorylase 2 | Microarray |
| hsa-miR-15a | *VEGFA* | 7422 | vascular endothelial growth factor A | ELISA//Luciferase reporter assay |
| hsa-miR-15a | *VPS45* | 11311 | vacuolar protein sorting 45 homolog (S. cerevisiae) | Microarray |
| hsa-miR-15a | *WIPF1* | 7456 | WAS/WASL interacting protein family, member 1 | Microarray |
| hsa-miR-15a | *WNT3A* | 89780 | wingless-type MMTV integration site family, member 3A | Luciferase reporter assay |
| hsa-miR-15a | *WT1* | 7490 | Wilms tumor 1 | qRT-PCR//proteomics analysis |
| hsa-miR-15a | *ZNF559* | 84527 | zinc finger protein 559 | Microarray |
| hsa-miR-15a | *CLCN3* | 1182 | chloride channel, voltage-sensitive 3 | Luciferase reporter assay//Microarray//Northern blot//qRT-PCR//Western blot |
| hsa-miR-15a | *CRKL* | 1399 | v-crk sarcoma virus CT10 oncogene homolog (avian)-like | Luciferase reporter assay//Microarray//Northern blot//qRT-PCR//Western blot |
| hsa-miR-15a | *FGF7* | 2252 | fibroblast growth factor 7 | Luciferase reporter assay//qRT-PCR |
| hsa-miR-15a | *MN1* | 4330 | meningioma (disrupted in balanced translocation) 1 | Luciferase reporter assay//Microarray//Northern blot//qRT-PCR//Western blot |
| hsa-miR-15a | *WNT3A* | 89780 | wingless-type MMTV integration site family, member 3A | Luciferase reporter assay//qRT-PCR |
| hsa-miR-17 | *APP* | 351 | amyloid beta (A4) precursor protein | Luciferase reporter assay//Western blot |
| hsa-miR-17 | *BCL2* | 596 | B-cell CLL/lymphoma 2 | Luciferase reporter assay |
| hsa-miR-17 | *BCL2L11* | 10018 | BCL2-like 11 (apoptosis facilitator) | Luciferase reporter assay//Western blot |
| hsa-miR-17 | *BMPR2* | 659 | bone morphogenetic protein receptor, type II (serine/threonine kinase) | Luciferase reporter assay//qRT-PCR//Western blot |
| hsa-miR-17 | *CCL1* | 6346 | chemokine (C-C motif) ligand 1 | Luciferase reporter assay |
| hsa-miR-17 | *CCND1* | 595 | cyclin D1 | Luciferase reporter assay//qRT-PCR//Western blot |
| hsa-miR-17 | *CCND2* | 894 | cyclin D2 | Luciferase reporter assay |
| hsa-miR-17 | *CDKN1A* | 1026 | cyclin-dependent kinase inhibitor 1A (p21, Cip1) | qRT-PCR//Luciferase reporter assay//Western blot |
| hsa-miR-17 | *DNAJC27* | 51277 | DnaJ (Hsp40) homolog, subfamily C, member 27 | Luciferase reporter assay |
| hsa-miR-17 | *E2F1* | 1869 | E2F transcription factor 1 | qRT-PCR |
| hsa-miR-17 | *E2F3* | 1871 | E2F transcription factor 3 | Luciferase reporter assay |
| hsa-miR-17 | *FBXO31* | 79791 | F-box protein 31 | Luciferase reporter assay |
| hsa-miR-17 | *GPR137B* | 7107 | G-protein coupled receptor 137B | Luciferase reporter assay |
| hsa-miR-17 | *ICAM1* | 3383 | intercellular adhesion molecule 1 | Luciferase reporter assay |
| hsa-miR-17 | *JAK1* | 3716 | Janus kinase 1 | Luciferase reporter assay//Western blot |
| hsa-miR-17 | *MAP3K12* | 7786 | mitogen-activated protein kinase kinase kinase 12 | Luciferase reporter assay |
| hsa-miR-17 | *MAPK9* | 5601 | mitogen-activated protein kinase 9 | qRT-PCR//Luciferase reporter assay//Western blot |
| hsa-miR-17 | *MEF2D* | 4209 | myocyte enhancer factor 2D | Luciferase reporter assay |
| hsa-miR-17 | *MUC17* | 140453 | mucin 17, cell surface associated | Immunohistochemistry//Microarray//qRT-PCR |
| hsa-miR-17 | *MYC* | 4609 | v-myc myelocytomatosis viral oncogene homolog (avian) | Western blot |
| hsa-miR-17 | *NCOA3* | 8202 | nuclear receptor coactivator 3 | Luciferase reporter assay//Northern blot//qRT-PCR//Western blot |
| hsa-miR-17 | *NPAT* | 4863 | nuclear protein, ataxia-telangiectasia locus | Luciferase reporter assay |
| hsa-miR-17 | *OBFC2A* | 64859 | oligonucleotide/oligosaccharide-binding fold containing 2A | Luciferase reporter assay |
| hsa-miR-17 | *PKD2* | 5311 | polycystic kidney disease 2 (autosomal dominant) | Luciferase reporter assay//qRT-PCR//Western blot |
| hsa-miR-17 | *PTEN* | 5728 | phosphatase and tensin homolog | Luciferase reporter assay//qRT-PCR//Western blot |
| hsa-miR-17 | *PTPRO* | 5800 | protein tyrosine phosphatase, receptor type, O | Luciferase reporter assay//Western blot |
| hsa-miR-17 | *RB1* | 5925 | retinoblastoma 1 | Luciferase reporter assay |
| hsa-miR-17 | *RBL1* | 5933 | retinoblastoma-like 1 (p107) | Luciferase reporter assay |
| hsa-miR-17 | *RBL2* | 5934 | retinoblastoma-like 2 (p130) | Luciferase reporter assay |
| hsa-miR-17 | *RUNX1* | 861 | runt-related transcription factor 1 | Luciferase reporter assay |
| hsa-miR-17 | *SELE* | 6401 | selectin E | Luciferase reporter assay |
| hsa-miR-17 | *SMAD4* | 4089 | SMAD family member 4 | Luciferase reporter assay//Microarray |
| hsa-miR-17 | *TGFBR2* | 7048 | transforming growth factor, beta receptor II (70/80kDa) | Immunoblot//Luciferase reporter assay//Microarray//qRT-PCR//Western blot |
| hsa-miR-17 | *THBS1* | 7057 | thrombospondin 1 | Microarray//qRT-PCR |
| hsa-miR-17 | *TNFSF12* | 8742 | tumor necrosis factor (ligand) superfamily, member 12 | Luciferase reporter assay |
| hsa-miR-17 | *VEGFA* | 7422 | vascular endothelial growth factor A | ELISA//Luciferase reporter assay |
| hsa-miR-17 | *WEE1* | 7465 | WEE1 homolog (S.pombe) | Luciferase reporter assay |
| hsa-miR-17 | *YES1* | 7525 | v-yes-1 Yamaguchi sarcoma viral oncogene homolog 1 | Luciferase reporter assay |
| hsa-miR-17 | *ZNFX1* | 57169 | zinc finger, NFX1-type containing 1 | Luciferase reporter assay |
| hsa-miR-17 | *PTEN* | 5728 | phosphatase and tensin homolog | Luciferase reporter assay//Western blot |
| hsa-miR-181a | *ATM* | 472 | ataxia telangiectasia mutated | Immunoblot//Luciferase reporter assay//qRT-PCR |
| hsa-miR-181a | *BCL2* | 596 | B-cell CLL/lymphoma 2 | Luciferase reporter assay//qRT-PCR//Western blot |
| hsa-miR-181a | *BCL2L11* | 10018 | BCL2-like 11 (apoptosis facilitator) | Luciferase reporter assay//qRT-PCR//Western blot |
| hsa-miR-181a | *CDKN1B* | 1027 | cyclin-dependent kinase inhibitor 1B (p27, Kip1) | Luciferase reporter assay//Western blot |
| hsa-miR-181a | *CDX2* | 1045 | caudal type homeobox 2 | Luciferase reporter assay |
| hsa-miR-181a | *DDIT4* | 54541 | DNA-damage-inducible transcript 4 | Immunoblot//Luciferase reporter assay//qRT-PCR |
| hsa-miR-181a | *GATA6* | 2627 | GATA binding protein 6 | Luciferase reporter assay |
| hsa-miR-181a | *HIPK2* | 28996 | homeodomain interacting protein kinase 2 | Immunoblot//Luciferase reporter assay//qRT-PCR |
| hsa-miR-181a | *HRAS* | 3265 | v-Ha-ras Harvey rat sarcoma viral oncogene homolog | Luciferase reporter assay//qRT-PCR//Western blot |
| hsa-miR-181a | *KAT2B* | 8850 | K(lysine) acetyltransferase 2B | Western blot//Luciferase reporter assay |
| hsa-miR-181a | *NLK* | 51701 | nemo-like kinase | Luciferase reporter assay |
| hsa-miR-181a | *PLAG1* | 5324 | pleiomorphic adenoma gene 1 | Western blot//Luciferase reporter assay//Microarray |
| hsa-miR-181a | *PROX1* | 5629 | prospero homeobox 1 | Luciferase reporter assay//qRT-PCR//Western blot |
| hsa-miR-181a | *ZNF763* | 284390 | zinc finger protein 763 | Luciferase reporter assay//Microarray//Western blot |
| hsa-miR-181a | *BCL2* | 596 | B-cell CLL/lymphoma 2 | Luciferase reporter assay |
| hsa-miR-181a | *DUSP5* | 1847 | dual specificity phosphatase 5 | Luciferase reporter assay//Western blot |
| hsa-miR-181a | *DUSP6* | 1848 | dual specificity phosphatase 6 | Luciferase reporter assay//Western blot |
| hsa-miR-181a | *PRAP1* | 118471 | poly (ADP-ribose) polymerase 1 | Luciferase reporter assay |
| hsa-miR-181a | *PTPN11* | 5781 | protein tyrosine phosphatase, non-receptor type 11 | Luciferase reporter assay//Western blot |
| hsa-miR-181a | *PTPN22* | 26191 | protein tyrosine phosphatase, non-receptor type 22 (lymphoid) | Luciferase reporter assay//Western blot |
| hsa-miR-181a | *RALA* | 5898 | v-ral simian leukemia viral oncogene homolog A (ras related) | Flow//Luciferase reporter assay//Microarray//qRT-PCR//Western blot |
| hsa-miR-181a | *RNF2* | 6045 | ring finger protein 2 | Luciferase reporter assay//Microarray//qRT-PCR//Western blot |
| hsa-miR-181a | *SIRT1* | 23411 | sirtuin 1 | Immunoblot//Luciferase reporter assay//qRT-PCR |
| hsa-miR-181d | *BCL2* | 596 | B-cell CLL/lymphoma 2 | Luciferase reporter assay//qRT-PCR//Western blot |
| hsa-miR-181d | *HRAS* | 3265 | v-Ha-ras Harvey rat sarcoma viral oncogene homolog | FACS//Immunohistochemistry//Luciferase reporter assay//qRT-PCR//Western blot |
| hsa-miR-185 | *AKT1* | 207 | v-akt murine thymoma viral oncogene homolog 1 | Flow//Immunoblot//Microarray//qRT-PCR |
| hsa-miR-185 | *CCNE1* | 898 | cyclin E1 | Flow//Immunoblot//Microarray//qRT-PCR |
| hsa-miR-185 | *CDC42* | 998 | cell division cycle 42 | Luciferase reporter assay//qRT-PCR//Western blot |
| hsa-miR-185 | *CDK6* | 1021 | cyclin-dependent kinase 6 | Flow//Immunoblot//Microarray//qRT-PCR//Western blog |
| hsa-miR-185 | *CORO2B* | 10391 | coronin, actin binding protein, 2B | Flow//Immunoblot//Microarray//qRT-PCR |
| hsa-miR-185 | *HMGA2* | 8091 | high mobility group AT-hook 2 | Flow//Immunoblot//Microarray//qRT-PCR |
| hsa-miR-185 | *NTRK3* | 4916 | neurotrophic tyrosine kinase, receptor, type 3 | Luciferase reporter assay |
| hsa-miR-185 | *RHOA* | 387 | ras homolog family member A | Luciferase reporter assay//qRT-PCR//Western blot |
| hsa-miR-185 | *SIX1* | 6495 | SIX homeobox 1 | Luciferase reporter assay//qRT-PCR//Quantitative proteomic approach//Western blot |
| hsa-miR-18a | *CCNL1* | 57018 | cyclin L1 | Microarray//Northern blot |
| hsa-miR-18a | *CSRNP3* | 80034 | cysteine-serine-rich nuclear protein 3 | Microarray//Northern blot |
| hsa-miR-18a | *CTGF* | 1490 | connective tissue growth factor | Western blot//Microarray//Northern blot |
| hsa-miR-18a | *ESR1* | 2099 | estrogen receptor 1 | Western blot |
| hsa-miR-18a | *HSF2* | 3298 | heat shock transcription factor 2 | Immunofluorescence//In situ hybridization//Luciferase reporter assay//qRT-PCR//Western blot |
| hsa-miR-18a | *NCOA3* | 8202 | nuclear receptor coactivator 3 | Western blot |
| hsa-miR-18a | *NR3C1* | 2908 | nuclear receptor subfamily, group C, member 1 (glucocorticoid receptor) | Luciferase reporter assay |
| hsa-miR-18a | *PTEN* | 5728 | phosphatase and tensin homolog | Luciferase reporter assay//qRT-PCR//Western blot |
| hsa-miR-18a | *SMAD4* | 4089 | SMAD family member 4 | Immunoblot//Luciferase reporter assay//Microarray//qRT-PCR//Western blot |
| hsa-miR-18a | *SPPL3* | 121665 | signal peptide peptidase like 3 | Microarray//Northern blot |
| hsa-miR-18a | *TGFBR2* | 7048 | transforming growth factor, beta receptor II (70/80kDa) | Luciferase reporter assay//Microarray |
| hsa-miR-18a | *THRA* | 7067 | thyroid hormone receptor, alpha | Microarray//Northern blot |
| hsa-miR-18a | *TNFSF11* | 8600 | tumor necrosis factor (ligand) superfamily, member 11 | Microarray//Northern blot |
| hsa-miR-18a | *TSC22D3* | 1831 | TSC22 domain family, member 3 | qRT-PCR |
| hsa-miR-18a | *ATM* | 472 | ataxia telangiectasia mutated | Luciferase reporter assay//qRT-PCR//Western blot |
| hsa-miR-18b | *ESR1* | 2099 | estrogen receptor 1 | Luciferase reporter assay//Microarray |
| hsa-miR-191 | *IL1A* | 3552 | interleukin 1, alpha | Immunohistochemistry//Immunoprecipitaion//Luciferase reporter assay//Microarray//qRT-PCR |
| hsa-miR-191 | *SOX4* | 6659 | SRY (sex determining region Y)-box 4 | Immunohistochemistry//Immunoprecipitaion//Luciferase reporter assay//Microarray//qRT-PCR |
| hsa-miR-191 | *TMC7* | 79905 | transmembrane channel-like 7 | Immunohistochemistry//Immunoprecipitaion//Luciferase reporter assay//Microarray//qRT-PCR |
| hsa-miR-191 | *MDM4* | 4194 | Mdm4 p53 binding protein homolog (mouse) | Luciferase reporter assay |
| hsa-miR-191 | *NDST1* | 3340 | N-deacetylase/N-sulfotransferase (heparan glucosaminyl) 1 | GFP reporter assay//qRT-PCR//Western blot |
| hsa-miR-192 | *ABCA8* | 10351 | ATP-binding cassette, sub family A (ABC1), member 8 | Microarray |
| hsa-miR-192 | *ABCC3* | 8714 | ATP-binding cassette, sub-family C (CFTR/MRP), member 3 | Microarray |
| hsa-miR-192 | *ABCG2* | 9429 | ATP-binding cassette, sub family G (WHITE), member 2 | Microarray |
| hsa-miR-192 | *AKAP9* | 10142 | A kinase (PRKA) anchor protein (yotiao) 9 | Microarray |
| hsa-miR-192 | *ALCAM* | 214 | activated leukocyte cell adhesion molecule | Microarray |
| hsa-miR-192 | *ATP10D* | 57205 | ATPase, class V, type 10D | Microarray |
| hsa-miR-192 | *ATXN7* | 6314 | ataxin 7 | Microarray |
| hsa-miR-192 | *B3GALNT1* | 8706 | beta- 1,3-N-acetylgalactosaminyltransferase 1 (globoside blood group) | Microarray |
| hsa-miR-192 | *BARD1* | 580 | BRCA1 associated RING domain 1 | Microarray |
| hsa-miR-192 | *BCL2* | 596 | B-cell CLL/lymphoma 2 | Luciferase reporter assay//qRT-PCR |
| hsa-miR-192 | *BRD3* | 8019 | bromodomain containing 3 | Microarray |
| hsa-miR-192 | *C1D* | 10438 | C1D nuclear receptor corepressor | Microarray |
| hsa-miR-192 | *CADM1* | 23705 | cell adhesion molecule 1 | Microarray |
| hsa-miR-192 | *CD164* | 8763 | CD164 molecule, sialomucin | Microarray |
| hsa-miR-192 | *CDC7* | 8317 | cell division cycle 7 | Luciferase reporter assay//qRT-PCR//Western blot |
| hsa-miR-192 | *CDKN1B* | 1027 | cyclin-dependent kinase inhibitor 1B (p27, Kip1) | Luciferase reporter assay |
| hsa-miR-192 | *CLIC1* | 1192 | chloride intracellular channel 1 | Microarray |
| hsa-miR-192 | *CUL3* | 8452 | cullin 3 | Microarray |
| hsa-miR-192 | *CUL5* | 8065 | cullin 5 | Luciferase reporter assay//qRT-PCR//Western blot |
| hsa-miR-192 | *DDOST* | 1650 | dolichyl-diphosphooligosaccharide-protein glysosyltransferase subunit (non-catalytic) | Microarray |
| hsa-miR-192 | *DDX3X* | 1654 | DEAD (Asp-Glu-Ala-Asp) box polypeptide 3, X-linked | Microarray |
| hsa-miR-192 | *DLG5* | 9231 | discs, large homolog 5 (Drosophila) | Luciferase reporter assay//qRT-PCR |
| hsa-miR-192 | *DTL* | 51514 | denticleless E3 ubiquitin protein ligase homolog (Drosophila) | Luciferase reporter assay//qRT-PCR |
| hsa-miR-192 | *E2F5* | 1875 | E2F transcription factor 5, p130-binding | Microarray |
| hsa-miR-192 | *EGR1* | 1958 | early growth response 1 | Microarray |
| hsa-miR-192 | *ENOSF1* | 55556 | enolase superfamily member 1 | Microarray |
| hsa-miR-192 | *ENTPD3* | 956 | ectonucleoside triphosphate diphosphohydrolase 3 | Microarray |
| hsa-miR-192 | *ERCC3* | 2071 | excision repair cross-complementing rodent repair deficiency, complementation group 3 | Luciferase reporter assay//qRT-PCR//Western blot |
| hsa-miR-192 | *ERLIN2* | 11160 | ER lipid raft associated 2 | Microarray |
| hsa-miR-192 | *GOLGA6A* | 342096 | golgin A6 family, member A | Microarray |
| hsa-miR-192 | *GRIA1* | 2890 | glutamate receptor, ionotropic, AMPA1 (alpha 1) | Microarray |
| hsa-miR-192 | *HOXA10* | 3206 | homeobox A10 | Luciferase reporter assay//qRT-PCR |
| hsa-miR-192 | *HRH1* | 3269 | histamine receptor H1 | Luciferase reporter assay//qRT-PCR |
| hsa-miR-192 | *HSP90B1* | 7184 | heat shock protein 90kDa beta (Grp94), member 1 | Microarray |
| hsa-miR-192 | *KIDINS220* | 57498 | kinase D-interacting substrate, 220kDa | Microarray |
| hsa-miR-192 | *KIF20B* | 9585 | kinesin family member 20B | Luciferase reporter assay//qRT-PCR |
| hsa-miR-192 | *LMNB2* | 84823 | lamin B2 | Luciferase reporter assay//qRT-PCR//Western blot |
| hsa-miR-192 | *LOXL2* | 4017 | ysyl oxidase-like 2 | Microarray |
| hsa-miR-192 | *MAD2L1* | 4085 | MAD2 mitotic arrest deficient-like 1 (yeast) | Luciferase reporter assay//qRT-PCR//Western blot |
| hsa-miR-192 | *MAP3K1* | 4214 | mitogen-activated protein kinase kinase kinase 1, E3 ubiquitin protein ligase | Microarray |
| hsa-miR-192 | *MCM10* | 55388 | minichromosome maintenance complex component 10 | Luciferase reporter assay//qRT-PCR |
| hsa-miR-192 | *MFSD10* | 10227 | major facilitator superfamily domain containing 10 | Microarray |
| hsa-miR-192 | *MIS12* | 79003 | MIS12, MIND kinetochore complex component, homolog (S. pombe) | Luciferase reporter assay//qRT-PCR |
| hsa-miR-192 | *MSN* | 4478 | moesin | Microarray |
| hsa-miR-192 | *ODC1* | 4953 | ornithine decarboxylase, structural 1 | Microarray |
| hsa-miR-192 | *PANX1* | 24145 | pannexin 1 | Microarray |
| hsa-miR-192 | *PDE2A* | 5138 | phosphodiesterase 2A, cGMP-stimulated | Microarray |
| hsa-miR-192 | *PERP* | 64065 | PERP, TP53 apoptosis effector | Microarray |
| hsa-miR-192 | *PIK3R4* | 30849 | phosphoinositide-3-kinase, regulatory subunit 4 | Microarray |
| hsa-miR-192 | *PIM1* | 5292 | pim-1 oncogene | Luciferase reporter assay//qRT-PCR |
| hsa-miR-192 | *PRPF38A* | 84950 | PRP38 pre-mRNA processing factor 38 (yeast) domain containing A | Luciferase reporter assay//qRT-PCR |
| hsa-miR-192 | *PTP4A3* | 11156 | protein tyrosine phosphatase type IVA, member 3 | Microarray |
| hsa-miR-192 | *RAB2A* | 5862 | RAB2A, member RAS oncogene family | Microarray |
| hsa-miR-192 | *RABGAP1* | 23637 | RAB GTPase activating protein 1 | Microarray |
| hsa-miR-192 | *RACGAP1* | 29127 | Rac GTPase activating protein 1 | Luciferase reporter assay//qRT-PCR |
| hsa-miR-192 | *RANBP3* | 8498 | RAN binding protein 3 | Microarray |
| hsa-miR-192 | *RBL2* | 5934 | retinoblastoma-like 2 (p130) | Microarray |
| hsa-miR-192 | *RRM1* | 6240 | ribonucleotide reductase M1 | Microarray |
| hsa-miR-192 | *SEMA4D* | 10507 | sema domain, immunoglobulin domain (Ig), transmembrane domain (TM) and short cytoplasmic domain, (semaphorin) 4D | Microarray |
| hsa-miR-192 | *SEPT10* | 151011 | septin 10 | Luciferase reporter assay//qRT-PCR |
| hsa-miR-192 | *SETD4* | 54093 | SET domain containing 4 | Microarray |
| hsa-miR-192 | *SMARCB1* | 6598 | SWI/SNF related, matrix associated, actin dependent regulator of chromatin, subfamily b, member 1 | Luciferase reporter assay//qRT-PCR |
| hsa-miR-192 | *SPARC* | 6678 | secreted protein, acidic, cysteine-rich (osteonectin) | Microarray |
| hsa-miR-192 | *STX7* | 8417 | syntaxin 7 | Microarray |
| hsa-miR-192 | *TFG* | 10342 | TRK-fused gene | Microarray |
| hsa-miR-192 | *TRAPPC2P1* | 10597 | trafficking protein particle complex 2 pseudogene 1 | Luciferase reporter assay |
| hsa-miR-192 | *WDR44* | 54521 | WD repeat domain 44 | Microarray |
| hsa-miR-192 | *WNK1* | 65125 | WNK lysine deficient protein kinase 1 | Luciferase reporter assay//Northern blot//qRT-PCR//Western blot |
| hsa-miR-192 | *XPA* | 7507 | xeroderma pigmentosum, complementation group A | Microarray |
| hsa-miR-192 | *ACVR2B* | 93 | activin A receptor, type IIB | Luciferase reporter assay//Western blot |
| hsa-miR-192 | *ERCC4* | 2072 | excision repair cross-complementing rodent repair deficiency, complementation group 4 | Luciferase reporter assay//qRT-PCR//Western blot |
| hsa-miR-192 | *RB1* | 5925 | retinoblastoma 1 | Immunoblot//Luciferase reporter assay//qRT-PCR//Western blot |
| hsa-miR-194 | *CDH2* | 1000 | cadherin 2, type 1, N-cadherin (neuronal) | Luciferase reporter assay//qRT-PCR//Western blot |
| hsa-miR-194 | *DNMT3A* | 1788 | DNA (cytosine-5-)-methyltransferase 3 alpha | Luciferase reporter assay//qRT-PCR//Western blot |
| hsa-miR-194 | *EP300* | 2033 | E1A binding protein p300 | Microarray |
| hsa-miR-194 | *HBEGF* | 1839 | heparin-binding EGF-like growth factor | Luciferase reporter assay//qRT-PCR//Western blot |
| hsa-miR-194 | *IGF1R* | 3480 | insulin-like growth factor receptor | Luciferase reporter assay//qRT-PCR//Western blot |
| hsa-miR-194 | *ITGA9* | 3680 | integrin, alpha 9 | Luciferase reporter assay//qRT-PCR//Western blot |
| hsa-miR-194 | *PTPN12* | 5782 | protein tyrosine phosphatase, non-receptor type 12 | Luciferase reporter assay//qRT-PCR//Western blot |
| hsa-miR-194 | *PTPN13* | 5783 | protein tyrosine phosphatase, non-receptor type 13 (APO-1/CD95 (Fas)-associated phosphatase) | Luciferase reporter assay//qRT-PCR//Western blot |
| hsa-miR-194 | *RAC1* | 5879 | ras-related C3 botulinum toxin substrate 1 (rho family, small GTP binding protein Rac1) | Luciferase reporter assay//qRT-PCR//Western blot |
| hsa-miR-194 | *SOCS2* | 8835 | suppressor of cytokine signaling 2 | Luciferase reporter assay//qRT-PCR//Western blot |
| hsa-miR-194 | *ACVR2B* | 93 | activin A receptor, type IIB | Luciferase reporter assay//Western blot |
| hsa-miR-194 | *SOX5* | 6660 | SRY (sex determining region Y)-box 5 | Luciferase reporter assay//qRT-PCR//Western blot |
| hsa-miR-199a-5p | *CCNL1* | 57018 | cyclin L1 | Microarray |
| hsa-miR-199a-5p | *DDR1* | 780 | discoidin domain receptor tyrosine kinase 1 | Luciferase reporter assay//qRT-PCR//Western blot |
| hsa-miR-199a-5p | *EDN1* | 1906 | endothelin 1 | Luciferase reporter assay |
| hsa-miR-199a-5p | *ETS2* | 2114 | v-ets erythroblastosis virus E26 oncogene homolog 2 (avian) | Microarray//Northern blot |
| hsa-miR-199a-5p | *EZH2* | 2146 | enhancer of zeste homolog 2 (Drosophila) | Luciferase reporter assay//Northern blot |
| hsa-miR-199a-5p | *HIF1A* | 3091 | hypoxia inducible factor 1, alpha subunit (basic helix-loop-helix transcription factor) | Luciferase reporter assay//qRT-PCR//Western blot |
| hsa-miR-199a-5p | *IKBKB* | 3551 | inhibitor of kappa light polypeptide gene enhancer in B-cells, kinase beta | Reporter assay//Western blot |
| hsa-miR-199a-5p | *JUNB* | 3726 | jun B proto-oncogene | Microarray//Northern blot |
| hsa-miR-199a-5p | *LIF* | 3976 | leukemia inhibitory factor | ELISA//Luciferase reporter assay |
| hsa-miR-199a-5p | *MAP3K11* | 4296 | mitogen-activated protein kinase kinase kinase 11 | Luciferase reporter assay//qRT-PCR//Western blot |
| hsa-miR-199a-5p | *MECP2* | 4204 | methyl CpG binding protein 2 (Rett syndrome) | Microarray//Northern blot |
| hsa-miR-199a-5p | *MED6* | 10001 | mediator complex subunit 6 | Microarray//Northern blot |
| hsa-miR-199a-5p | *SMARCA2* | 6595 | SWI/SNF related, matrix associated, actin dependent regulator of chromatin, subfamily a, member 2 | Luciferase reporter assay//qRT-PCR//Western blot |
| hsa-miR-199a-5p | *SOX9* | 6662 | SRY (sex determining region Y)-box 9 | qRT-PCR |
| hsa-miR-19a | *ARIH2* | 10425 | ariadne homolog 2 (Drosophila) | qRT-PCR |
| hsa-miR-19a | *ATXN1* | 6310 | ataxin 1 | Luciferase reporter assay//qRT-PCR//Western blot |
| hsa-miR-19a | *BCL2L11* | 10018 | BCL2-like 11 (apoptosis facilitator) | Luciferase reporter assay |
| hsa-miR-19a | *BMPR2* | 659 | bone morphogenetic protein receptor, type II (serine/threonine kinase) | Luciferase reporter assay//qRT-PCR//Western blot |
| hsa-miR-19a | *CCND1* | 595 | cyclin D1 | flow//Luciferase reporter assay//Microarray//qRT-PCR//Western blot |
| hsa-miR-19a | *ERBB4* | 2066 | v-erb-a erythroblastic leukemia viral oncogene homolog 4 (avian) | Luciferase reporter assay//Microarray |
| hsa-miR-19a | *ESR1* | 2099 | estrogen receptor 1 | Luciferase reporter assay//qRT-PCR//Western blot |
| hsa-miR-19a | *HOXA5* | 3202 | homebox A5 | Luciferase reporter assay |
| hsa-miR-19a | *KAT2B* | 8850 | K(lysine) acetyltransferase 2B | Western blot//Luciferase reporter assay |
| hsa-miR-19a | *MECP2* | 4204 | methyl CpG binding protein 2 (Rett syndrome) | Luciferase reporter assay |
| hsa-miR-19a | *NR4A2* | 4929 | nuclear receptor subfamily 4, group A, member 2 | Luciferase reporter assay//Microarray |
| hsa-miR-19a | *PRMT5* | 10419 | protein arginine methyltransferase 5 | Western blot |
| hsa-miR-19a | *PTEN* | 5728 | phosphatase and tensin homolog | qRT-PCR//Western blot |
| hsa-miR-19a | *SMAD4* | 4089 | SMAD family member 4 | Luciferase reporter assay//Microarray |
| hsa-miR-19a | *SOCS1* | 8651 | suppressor of cytokine signaling 1 | Luciferase reporter assay//Western blot |
| hsa-miR-19a | *TGFBR2* | 7048 | transforming growth factor, beta receptor II (70/80kDa) | Luciferase reporter assay//Microarray |
| hsa-miR-19a | *CUL5* | 8065 | cullin 5 | qRT-PCR//Western blot |
| hsa-miR-19a | *KIT* | 3815 | v-kit Hardy-Zuckerman 4 feline sarcoma viral oncogene homolog | Luciferase reporter assay//Microarray//Northern blot//qRT-PCR//Western blot |
| hsa-miR-200b | *BAP1* | 8314 | BRCA1 associated protein-1 (ubiquitin carboxy-terminal hydrolase) | Microarray |
| hsa-miR-200b | *EP300* | 2033 | E1A binding protein p300 | Microarray |
| hsa-miR-200b | *ETS1* | 2113 | v-ets erythroblastosis virus E26 oncogene homolog 1 (avian) | Immunocytochemistry//Luciferase reporter assay//qRT-PCR//Western blot |
| hsa-miR-200b | *FN1* | 2335 | fibronectin 1 | Microarray |
| hsa-miR-200b | *GATA4* | 2626 | GATA binding protein 4 | Microarray |
| hsa-miR-200b | *MATR3* | 9782 | matrin 3 | Luciferase reporter assay//Microarray//qRT-PCR |
| hsa-miR-200b | *PTPN12* | 5782 | protein tyrosine phosphatase, non-receptor type 12 | Northern blot//qRT-PCR//Western blot |
| hsa-miR-200b | *RERE* | 473 | arginine-glutamic acid dipeptide (RE) repeats | Luciferase reporter assay |
| hsa-miR-200b | *SIP1* | 8487 | gem (nuclear organelle) associated protein 2 | Luciferase reporter assay//Microarray//Western blot |
| hsa-miR-200b | *WASF3* | 10810 | WAS protein family, member 3 | Luciferase reporter assay//qRT-PCR |
| hsa-miR-200b | *ZEB1* | 6935 | zinc finger E-box binding homeobox 1 | Immunohistochemistry//Luciferase reporter assay//qRT-PCR |
| hsa-miR-200b | *ZEB2* | 9839 | zinc finger E-box binding homeobox 2 | Immunohistochemistry//Luciferase reporter assay//qRT-PCR |
| hsa-miR-200b | *ZFPM2* | 23414 | zinc finger protein, multitype 2 | Luciferase reporter assay//Western blot |
| hsa-miR-200b | *BMI1* | 648 | BMI1 polycomb ring finger oncogene | Luciferase reporter assay//Microarray//qRT-PCR//Western blot |
| hsa-miR-200b | *E2F3* | 1871 | E2F transcription factor 3 | Flow//Luciferase reporter assay//Western blot |
| hsa-miR-200b | *FLT1* | 2321 | fms-related tyrosine kinase 1 | Luciferase reporter assay//Western blot |
| hsa-miR-200b | *KDR* | 3791 | kinase insert domain receptor (a type III receptor tyrosine kinase) | Luciferase reporter assay//Western blot |
| hsa-miR-200b | *RND3* | 390 | Rho family GTPase 3 | Luciferase reporter assay |
| hsa-miR-200b | *RNF2* | 6045 | ring finger protein 2 | Luciferase reporter assay//Microarray//qRT-PCR//Western blot |
| hsa-miR-200b | *VEGFA* | 7422 | vascular endothelial growth factor A | Luciferase reporter assay//Western blot |
| hsa-miR-20a | *APP* | 351 | amyloid beta (A4) precursor protein | Luciferase reporter assay//Western blot |
| hsa-miR-20a | *BCL2* | 596 | B-cell CLL/lymphoma 2 | Luciferase reporter assay |
| hsa-miR-20a | *BMPR2* | 659 | bone morphogenetic protein receptor, type II (serine/threonine kinase) | Luciferase reporter assay//qRT-PCR//Western blot |
| hsa-miR-20a | *BNIP2* | 663 | BCL2/adenovirus E1B 19kDa interacting protein2 | qRT-PCR//Western blot |
| hsa-miR-20a | *CCND1* | 595 | cyclin D1 | Luciferase reporter assay//qRT-PCR//Western blot |
| hsa-miR-20a | *CCND2* | 894 | cyclin D2 | Luciferase reporter assay |
| hsa-miR-20a | *CDKN1A* | 1026 | cyclin-dependent kinase inhibitor 1A (p21, Cip1) | qRT-PCR//Luciferase reporter assay//Western blot |
| hsa-miR-20a | *E2F1* | 1869 | E2F transcription factor 1 | Western blot |
| hsa-miR-20a | *E2F3* | 1871 | E2F transcription factor 3 | Luciferase reporter assay |
| hsa-miR-20a | *HIF1A* | 3091 | hypoxia inducible factor 1, alpha subunit (basic helix-loop-helix transcription factor) | Luciferase reporter assay//Western blot//Northern blot |
| hsa-miR-20a | *MAP3K12* | 7786 | mitogen-activated protein kinase kinase kinase 12 | Luciferase reporter assay |
| hsa-miR-20a | *MAPK9* | 5601 | mitogen-activated protein kinase 9 | Luciferase reporter assay |
| hsa-miR-20a | *MEF2D* | 4209 | myocyte enhancer factor 2D | Luciferase reporter assay |
| hsa-miR-20a | *MUC17* | 140453 | mucin 17, cell surface associated | Immunohistochemistry//Microarray//qRT-PCR |
| hsa-miR-20a | *MYC* | 4609 | v-myc myelocytomatosis viral oncogene homolog (avian) | Western blot |
| hsa-miR-20a | *NRAS* | 4893 | neuroblastoma RAS viral (v-ras) oncogene homolog | Western blot |
| hsa-miR-20a | *PTEN* | 5728 | phosphatase and tensin homolog | Luciferase reporter assay//qRT-PCR//Western blot |
| hsa-miR-20a | *RB1* | 5925 | retinoblastoma 1 | Luciferase reporter assay |
| hsa-miR-20a | *RBL1* | 5933 | retinoblastoma-like 1 (p107) | Luciferase reporter assay |
| hsa-miR-20a | *RBL2* | 5934 | retinoblastoma-like 2 (p130) | Luciferase reporter assay |
| hsa-miR-20a | *RUNX1* | 861 | runt-related transcription factor 1 | Luciferase reporter assay//qRT-PCR//Western blot |
| hsa-miR-20a | *SMAD4* | 4089 | SMAD family member 4 | Microarray |
| hsa-miR-20a | *TGFBR2* | 7048 | transforming growth factor, beta receptor II (70/80kDa) | Immunoblot//Luciferase reporter assay//Microarray//qRT-PCR//Western blot |
| hsa-miR-20a | *THBS1* | 7057 | thrombospondin 1 | Microarray//qRT-PCR |
| hsa-miR-20a | *VEGFA* | 7422 | vascular endothelial growth factor A | ELISA//Luciferase reporter assay |
| hsa-miR-20a | *WEE1* | 7465 | WEE1 homolog (S.pombe) | Luciferase reporter assay |
| hsa-miR-20a | *EGLN3* | 112399 | egl nine homolog 3 (C.elegans) | Luciferase reporter assay//Western blot |
| hsa-miR-20a | *IRF2* | 3660 | interferon regulatory factor 2 | Luciferase reporter assay//Microarray//Northern blot//qRT-PCR//Western blot |
| hsa-miR-20a | *KIT* | 3815 | v-kit Hardy-Zuckerman 4 feline sarcoma viral oncogene homolog | Luciferase reporter assay//Microarray//Northern blot//qRT-PCR//Western blot |
| hsa-miR-20b | *ARID4B* | 51742 | AT rich interactive domain 4B (RBP1-like) | Luciferase reporter assay |
| hsa-miR-20b | *BAMBI* | 25805 | BMP and activin membrane-bound inhibitor homolog (Xenopus laevis) | Luciferase reporter assay//qRT-PCR |
| hsa-miR-20b | *CDKN1A* | 1026 | cyclin-dependent kinase inhibitor 1A (p21, Cip1) | qRT-PCR//Luciferase reporter assay//Western blot |
| hsa-miR-20b | *CRIM1* | 51232 | cysteine rich transmembrane BMP regulator 1 (chordin-like) | Luciferase reporter assay//qRT-PCR |
| hsa-miR-20b | *ESR1* | 2099 | estrogen receptor 1 | Western blot |
| hsa-miR-20b | *HIF1A* | 3091 | hypoxia inducible factor 1, alpha subunit (basic helix-loop-helix transcription factor) | qRT-PCR//ELISA//ChIP//Western blot |
| hsa-miR-20b | *HIPK3* | 10114 | homeodomain interacting protein kinase 3 | Luciferase reporter assay |
| hsa-miR-20b | *MUC17* | 140453 | mucin 17, cell surface associated | Immunohistochemistry//Microarray//qRT-PCR |
| hsa-miR-20b | *MYLIP* | 29116 | myosin regulatory light chain interacting protein | Luciferase reporter assay |
| hsa-miR-20b | *PPARG* | 5468 | peroxisome proliferator-activated receptor gamma | Luciferase reporter assay//qRT-PCR |
| hsa-miR-20b | *STAT3* | 6774 | signal transducer and activator of transcription 3 (acute-phase response factor) | qRT-PCR//ELISA//ChIP//Western blot |
| hsa-miR-20b | *VEGFA* | 7422 | vascular endothelial growth factor A | ELISA//Luciferase reporter assay |
| hsa-miR-20b | *EFNB2* | 1948 | ephrin-B2 | Luciferase reporter assay |
| hsa-miR-20b | *EPHB4* | 2050 | EPH receptor B4 | Luciferase reporter assay |
| hsa-miR-21 | *ANKRD46* | 157567 | ankyrin repeat domain 46 | Immunoblot//Immunohistochemistry//Luciferase reporter assay//Microarray//qRT-PCR//Western blot |
| hsa-miR-21 | *APAF1* | 317 | apoptotic peptidase activating factor 1 | Luciferase reporter assay//qRT-PCR//Western blot |
| hsa-miR-21 | *BASP1* | 10409 | brain abundant, membrane attached signal protein 1 | Luciferase reporter assay//Quantitative proteomic approach |
| hsa-miR-21 | *BCL2* | 596 | B-cell CLL/lymphoma 2 | Luciferase reporter assay//Western blot |
| hsa-miR-21 | *BMPR2* | 659 | bone morphogenetic protein receptor, type II (serine/threonine kinase) | Luciferase reporter assay//qRT-PCR//Western blot |
| hsa-miR-21 | *BTG2* | 7832 | BTG family, member 2 | semi-qRT-PCR//GFP reporter assay//Western blot |
| hsa-miR-21 | *CCR1* | 1230 | chemokine (C-C motif) receptor 1 | Luciferase reporter assay |
| hsa-miR-21 | *CDC25A* | 993 | cell division cycle 25A | Microarray//Northern blot//qRT-PCR |
| hsa-miR-21 | *CDK2AP1* | 8099 | cyclin-dependent kinase 2 associated protein 1 | Luciferase reporter assay//qRT-PCR//Western blot |
| hsa-miR-21 | *DAXX* | 1616 | death-domain associated protein | Luciferase reporter assay//qRT-PCR//Western blot |
| hsa-miR-21 | *DERL1* | 79139 | derlin 1 | Luciferase reporter assay//Quantitative proteomic approach |
| hsa-miR-21 | *E2F1* | 1869 | E2F transcription factor 1 | qRT-PCR//Western blot |
| hsa-miR-21 | *E2F2* | 1870 | E2F transcription factor 2 | Northern blot |
| hsa-miR-21 | *EGFR* | 1956 | epidermal growth factor receptor | Luciferase reporter assay//Microarray//Western blot |
| hsa-miR-21 | *EIF2S1* | 1965 | eukaryotic translation initiation factor 2, subunit 1 alpha, 35kDa | Quantitative proteomic approach |
| hsa-miR-21 | *EIF4A2* | 1974 | eukaryotic translation initiation factor 4A2 | Immunoblot//Immunohistochemistry//Luciferase reporter assay//Microarray//qRT-PCR//Western blot |
| hsa-miR-21 | *ERBB2* | 2064 | v-erb-b2 erythroblastic leukemia viral oncogene homolog, neuro/glioblastoma derived oncogene homolog (avian) | qRT-PCR//Western blot |
| hsa-miR-21 | *FMOD* | 2331 | fibromodulin | Western blot |
| hsa-miR-21 | *HNRNPK* | 3190 | heterogenous nuclear ribonucleoprotein K | Luciferase reporter assay//qRT-PCR |
| hsa-miR-21 | *ICAM1* | 3383 | intercellular adhesion molecule 1 | Luciferase reporter assay//Microarray//qRT-PCR |
| hsa-miR-21 | *IL1B* | 3553 | interleukin 1, beta | Luciferase reporter assay//Microarray//qRT-PCR |
| hsa-miR-21 | *ISCU* | 23479 | iron-sulfur cluster scaffold homolog (E.coli) | Immunoblot//Immunohistochemistry//Luciferase reporter assay//qRT-PCR//Western blot |
| hsa-miR-21 | *JAG1* | 182 | jagged 1 | Flow//Immunoblot//Luciferase reporter assay//Microarray//qRT-PCR |
| hsa-miR-21 | *JMY* | 133746 | junction mediating and regulatory protein, p53 cofactor | Luciferase reporter assay//qRT-PCR |
| hsa-miR-21 | *LRRFIP1* | 9208 | leucine rich repeat (in FLII) interacting protein 1 | semi-qRT-PCR//Luciferase reporter assay//Western blot |
| hsa-miR-21 | *MARCKS* | 4082 | myristoylated alanine-rich protein kinase C substrate | Luciferase reporter assay//Western blot |
| hsa-miR-21 | *MEF2C* | 4208 | myocyte enhancer factor 2C | Immunofluorescence//In situ hybridization//Luciferase reporter assay |
| hsa-miR-21 | *MSH2* | 4436 | mutS homolog, colon cancer, nonpolyposis type 1 (E.coli) | Luciferase reporter assay//Northern blot//qRT-PCR//Western blot |
| hsa-miR-21 | *MSH6* | 2956 | mutS homolog 6 (E.coli) | Luciferase reporter assay//Northern blot//qRT-PCR//Western blot |
| hsa-miR-21 | *MTAP* | 4507 | methylthioadenosine phosphorylase | Western blot//Northern blot |
| hsa-miR-21 | *MYC* | 4609 | v-myc myelocytomatosis viral oncogene homolog (avian) | Northern blot |
| hsa-miR-21 | *NCAPG* | 64151 | non-SMC condensin I complex, subunit G | Luciferase reporter assay//Quantitative proteomic approach |
| hsa-miR-21 | *NCOA3* | 8202 | nuclear receptor coactivator 3 | Luciferase reporter assay |
| hsa-miR-21 | *NFIB* | 4781 | nuclear factor I/B | Luciferase reporter assay//qRT-PCR//Western blot |
| hsa-miR-21 | *PCBP1* | 5093 | poly(rC) binding protein 1 | Quantitative proteomic approach//Luciferase reporter assay |
| hsa-miR-21 | *PDCD4* | 27250 | programmed cell death 4 (neoplastic transformation inhibitor) | Luciferase reporter assay//Westren blot |
| hsa-miR-21 | *PDHA2* | 5161 | pyruvate dehydrogenase (lipoamide) alpha 2 | Quantitative proteomic approach |
| hsa-miR-21 | *PLAT* | 5327 | plasminogen activator, tissue | Luciferase reporter assay//Microarray//qRT-PCR |
| hsa-miR-21 | *PLOD3* | 8985 | procollagen-lysine, 2-oxoglutarate 5-dioxygenase 3 | Luciferase reporter assay//Quantitative proteomic approach |
| hsa-miR-21 | *PPIF* | 10105 | peptidylprolyl isomerase F | Luciferase reporter assay//qRT-PCR |
| hsa-miR-21 | *PTEN* | 5728 | phosphatase and tensin homolog | Luciferase reporter assay//Microarray//Northern blot//qRT-PCR//Western blot |
| hsa-miR-21 | *PTX3* | 5806 | pentraxin 3, long | Luciferase reporter assay |
| hsa-miR-21 | *RASA1* | 5921 | RAS p21 protein activator (GTPase activating protein) 1 | Luciferase reporter assay//Western blot |
| hsa-miR-21 | *RASGRP1* | 10125 | RAS guanyl releasing protein 1 (calcium and DAG-regulated) | qRT-PCR//Luciferase reporter assay//Western blot |
| hsa-miR-21 | *RECK* | 8434 | reversion-inducing-cysteine-rich protein with kazal motifs | Luciferase reporter assay//qRT-PCR//Western blot |
| hsa-miR-21 | *REST* | 5978 | RE1-silencing transcription factor | Luciferase reporter assay |
| hsa-miR-21 | *RHOB* | 388 | ras homolog family member B | Luciferase reporter assay//qRT-PCR//Western blot |
| hsa-miR-21 | *RPS7* | 6201 | ribosomal protein S7 | qRT-PCR//Quantitative proteomic approach |
| hsa-miR-21 | *RTN4* | 57142 | reticulon 4 | Luciferase reporter assay//Quantitative proteomic approach |
| hsa-miR-21 | *SERPINB5* | 5268 | serpin peptidase inhibitor, clade B (ovalbumin), member 5 | Luciferase reporter assay//Western blot |
| hsa-miR-21 | *SOX5* | 6660 | SRY (sex determining region Y)-box 5 | Western blot//Northern blot |
| hsa-miR-21 | *SPATS2L* | 26010 | spermatogenesis associated, serine-rich 2-like | Quantitative proteomic approach |
| hsa-miR-21 | *SPRY2* | 10253 | sprouty homolog 2 (Drosophila) | Luciferase reporter assay |
| hsa-miR-21 | *TGFBI* | 7045 | transforming growth factor, beta-induced, 68kDa | Luciferase reporter assay//Western blot |
| hsa-miR-21 | *TGFBR2* | 7048 | transforming growth factor, beta receptor II (70/80kDa) | Luciferase assay//qRT-PCR//Western blot |
| hsa-miR-21 | *TGFBR3* | 7049 | transforming growth factor, beta receptor III | Luciferase reporter assay//qRT-PCR |
| hsa-miR-21 | *TGIF1* | 7050 | TGF-induced factor homebox 1 | Western blot |
| hsa-miR-21 | *TIAM1* | 7074 | T-cell lymphoma invasion and metastasis 1 | Luciferase reporter assay//Microarray//Northern blot//qRT-PCR |
| hsa-miR-21 | *TIMP3* | 7078 | TIMP metallopeptidase inhibitor 3 | Luciferase reporter assay |
| hsa-miR-21 | *TM9SF3* | 56889 | transmembrane 9 superfamily member 3 | Luciferase reporter assay |
| hsa-miR-21 | *TNFAIP3* | 7128 | tumor necrosis factor, alpha-induced protein 3 | Luciferase reporter assay |
| hsa-miR-21 | *TOPORS* | 10210 | topoisomerase I binding, arginine/serine-rich, E3 ubiquitin protein ligase | Luciferase reporter assay//qRT-PCR |
| hsa-miR-21 | *TP53BP2* | 7159 | tumor protein p53 binding protein, 2 | Luciferase reporter assay//qRT-PCR |
| hsa-miR-21 | *TP63* | 8626 | tumor protein p63 | Luciferase reporter assay//qRT-PCR//Western blot |
| hsa-miR-21 | *TPM1* | 7168 | tropomyosin 1 (alpha) | Luciferase reporter assay//Western blot |
| hsa-miR-21 | *WFS1* | 7466 | Wolfram syndrome 1 (wolframin) | Quantitative proteomic approach |
| hsa-miR-21 | *WIBG* | 84305 | within bgcn homolog (Drosophila) | Quantitative proteomic approach |
| hsa-miR-21 | *ANP32A* | 8125 | acidic (leucine-rich) nuclear phosphoprotein 32 family, member A | Luciferase reporter assay//qRT-PCR//Western blot |
| hsa-miR-21 | *CCL20* | 6364 | chemokine (C-C motif) ligand 20 | ELISA//Immunofluorescence//Luciferase reporter assay |
| hsa-miR-21 | *DOCK4* | 9732 | dedicator of cytokinesis 4 | Immunoblot//Luciferase reporter assay//qRT-PCR |
| hsa-miR-21 | *DOCK5* | 80005 | dedicator of cytokinesis 5 | Immunoblot//Luciferase reporter assay//qRT-PCR |
| hsa-miR-21 | *DOCK7* | 85440 | dedicator of cytokinesis 7 | Immunoblot//Luciferase reporter assay//qRT-PCR |
| hsa-miR-21 | *DUSP10* | 11221 | dual specificity phosphatase 10 | Luciferase reporter assay//qRT-PCR//Western blot |
| hsa-miR-21 | *NFKB1* | 4790 | nuclear factor of kappa light polypeptide gene enhancer in B-cells | ChIP-seq |
| hsa-miR-21 | *PIAS3* | 10401 | protein inhibitor of activated STAT, 3 | Luciferase reporter assay |
| hsa-miR-21 | *PPARA* | 5465 | peroxisome proliferator-activated receptor alpha | Luciferase reporter assay |
| hsa-miR-21 | *SMARCA4* | 6597 | SWI/SNF related, matrix associated, actin dependent regulator of chromatin, subfamily a, member 4 | Luciferase reporter assay//qRT-PCR//Western blot |
| hsa-miR-21 | *SP1* | 6667 | Sp1 transcription factor | Luciferase reporter assay//Western blot |
| hsa-miR-214 | *DAPK1* | 1612 | death-associated protein kinase 1 | Microarray//qRT-PCR |
| hsa-miR-214 | *EZH2* | 2146 | enhancer of zeste homolog 2 (Drosophila) | Luciferase reporter assay//qRT-PCR//Nortern blot//Western blot |
| hsa-miR-214 | *MAP2K3* | 5606 | mitogen-activated protein kinase kinase 3 | GFP reporter assay//Microarray//qRT-PCR//Western blot |
| hsa-miR-214 | *MAPK8* | 5599 | mitogen-activated protein kinase 8 | GFP reporter assay//Microarray//qRT-PCR//Western blot |
| hsa-miR-214 | *PLXNB1* | 5364 | plexin B1 | GFP reporter assay//Immunohistochemistry//qRT-PCR//Western blot |
| hsa-miR-214 | *POU4F2* | 5458 | POU class 4 homeobox 2 | Flow//GFP reporter assay//Northern blot//qRT-PCR//Western blot |
| hsa-miR-214 | *PTEN* | 5728 | phosphatase and tensin homolog | Western blot//qRT-PCR//Luciferase reporter assay |
| hsa-miR-214 | *SRGAP1* | 57522 | SLIT-ROBO Rho GTPase activating protein 1 | In situ hybridization//Luciferase reporter assay//qRT-PCR//western blot |
| hsa-miR-214 | *ING4* | 51147 | inhibitor of growth family, member 4 | Luciferase reporter assay//qRT-PCR |
| hsa-miR-214 | *XBP1* | 7494 | X-box binding protein 1 | ELISA//Flow//Luciferase reporter assay//qRT-PCR//Western blot |
| hsa-miR-215 | *ACVR2B* | 93 | activin A receptor, type IIB | Luciferase reporter assay//Western blot |
| hsa-miR-215 | *WNK1* | 65125 | WNK lysine deficient protein kinase 1 | Luciferase reporter assay//Northern blot//qRT-PCR//Western blot |
| hsa-miR-221 | *BBC3* | 27113 | BCL2 binding component 3 | Immunohistochemistry//In situ hybridization//Luciferase reporter assay//Northern blot//Western blot |
| hsa-miR-221 | *BMF* | 90427 | Bcl2 modifying factor | Luciferase reporter assay//qRT-PCR//Western blot |
| hsa-miR-221 | *BNIP3* | 664 | BCL2/adenovirus E1B 19kDa interacting protein 3 | Luciferase reporter assay//Microarray//qRT-PCR//Western blot |
| hsa-miR-221 | *CDKN1B* | 1027 | cyclin-dependent kinase inhibitor 1B (p27, Kip1) | Luciferase reporter assay//Western blot |
| hsa-miR-221 | *CDKN1C* | 1028 | cyclin-dependent kinase inhibitor 1C (p57, Kip2) | Reporter assay |
| hsa-miR-221 | *CORO1A* | 11151 | coronin, actin binding protein 1A | Western blot |
| hsa-miR-221 | *DDIT4* | 54541 | DNA-damage-inducible transcript 4 | Luciferase reporter assay//Microarray//qRT-PCR//Western blot |
| hsa-miR-221 | *ESR1* | 2099 | estrogen receptor 1 | Luciferase reporter assay//qRT-PCR//Western blot |
| hsa-miR-221 | *FOS* | 2353 | FBJ osteosarcoma oncogene | qRT-PCR//Luciferase reporter assay//Western blot//Northern blot |
| hsa-miR-221 | *FOXO3* | 2309 | forkhead box O3 | qRT-PCR//ChIP//Luciferase reporter assay//Western blot//Northern blot |
| hsa-miR-221 | *HOXB5* | 3215 | homeobox B5 | Gluc assay |
| hsa-miR-221 | *ICAM1* | 3383 | intercellular adhesion molecule 1 | Luciferase reporter assay//Western blot |
| hsa-miR-221 | *KIT* | 3815 | v-kit Hardy-Zuckerman 4 feline sarcoma viral oncogene homolog | Luciferase reporter assay//qRT-PCR//Western blot |
| hsa-miR-221 | *NAIP* | 4671 | NLR family, apoptosis inhibitory protein | Western blot |
| hsa-miR-221 | *PTEN* | 5728 | phosphatase and tensin homolog | FACS//Flow//Immunohistochemistry//Luciferase reporter assay//qRT-PCRNorthern blot//Western blot |
| hsa-miR-221 | *SELE* | 6401 | selectin E | Luciferase reporter assay |
| hsa-miR-221 | *SSSCA1* | 10534 | Sjogren syndrome/scleroderma autoantigen 1 | Western blot |
| hsa-miR-221 | *TCEAL1* | 9338 | transcription elongation factor A (SII)-like 1 | Western blot |
| hsa-miR-221 | *TICAM1* | 148022 | intercellular adhesion molecule 1 | Luciferase reporter assay//Northern blot//qRT-PCR//Western blot |
| hsa-miR-221 | *TNFSF10* | 8743 | tumor necrosis factor (ligand) superfamily, member 10 | Western blot |
| hsa-miR-221 | *TP53* | 7157 | tumor protein p53 | Western blot |
| hsa-miR-221 | *DICER1* | 23405 | dicer 1, ribonuclease type III | Luciferase reporter assay |
| hsa-miR-221 | *DIRAS3* | 9077 | DIRAS family, GTP-binding RAS-like 3 | Immunohistochemistry//Luciferase reporter assay//qRT-PCR//Western blot |
| hsa-miR-221 | *ETS1* | 2113 | v-ets erythroblastosis virus E26 oncogene homolog 1 (avian) | Luciferase reporter assay//Northern blot//qRT-PCR//Western blot |
| hsa-miR-221 | *TIMP3* | 7078 | TIMP metallopeptidase inhibitor 3 | Flow//Immunohistochemistry//Luciferase reporter assay//qRT-PCR//Western blot |
| hsa-miR-223 | *CHUK* | 1147 | conserved helix-loop-helix ubiquitous kinase | Luciferase reporter assay//qRT-PCR//Western blot |
| hsa-miR-223 | *E2F1* | 1869 | E2F transcription factor 1 | Luciferase reporter assay//Western blot |
| hsa-miR-223 | *LMO2* | 4005 | LIM domain only 2 (rhombotin-like 1) | Luciferase reporter assay//qRT-PCR//Western blot |
| hsa-miR-223 | *MEF2C* | 4208 | myocyte enhancer factor 2C | Luciferase reporter assay |
| hsa-miR-223 | *NFIA* | 4774 | nuclear factor I/A | Luciferase reporter assay |
| hsa-miR-223 | *NFIX* | 4784 | nuclear factor I/X (CCAAT-binding transcription factor) | Luciferase reporter assay |
| hsa-miR-223 | *RHOB* | 388 | ras homolog family member B | Luciferase reporter assay//Western blot |
| hsa-miR-223 | *STMN1* | 3925 | stathmin 1 | Luciferase reporter assay//qRT-PCR//Western blot |
| hsa-miR-223 | *EPB41L3* | 23136 | erythrocyte membrane protein band 4.1-like 3 | Luciferase reporter assay//Western blot |
| hsa-miR-223 | *FBXW7* | 55294 | F-box and WD repeat domain containing 7, E3 ubiquitin protein ligase | Luciferase reporter assay//qRT-PCR//Western blot |
| hsa-miR-223 | *IGF1R* | 3480 | insulin-like growth factor receptor | Luciferase reporter assay//Western blot |
| hsa-miR-223 | *SLC2A4* | 6517 | solute carrier family 2 (facilitated glucose transporter), member 4 | Flow//Immunoblot//Immunofluorescence//Immunohistochemistry//Immunoprecipitaion//In situ hybridization//Luciferase reporter assay//Northern blot//qRT-PCR |
| hsa-miR-224 | *AP2M1* | 1173 | adaptor-related protein complex 2, mu 1 subunit | Luciferase reporter assay |
| hsa-miR-224 | *API5* | 8539 | apoptosis inhibitor 5 | Western blot//Luciferase reporter assay//qRT-PCR |
| hsa-miR-224 | *CD40* | 958 | CD40 molecule, TNF receptor superfamily member 5 | Microarray//qRT-PCR |
| hsa-miR-224 | *CDC42* | 998 | cell division cycle 42 | Luciferase reporter assay//qRT-PCR//Western blot |
| hsa-miR-224 | *CXCR4* | 7852 | chemokine (C-X-C motif) receptor 4 | Luciferase reporter assay//qRT-PCR//Western blot |
| hsa-miR-224 | *EDNRA* | 1909 | endothelial receptor type A | Immunofluorescence//In situ hybridization//Luciferase reporter assay//Northern blot//qRT-PCR//Western blot |
| hsa-miR-224 | *EYA4* | 2070 | eyes absent homolog 4 (Drosophila) | Immunofluorescence//In situ hybridization//Luciferase reporter assay//Northern blot//qRT-PCR//Western blot |
| hsa-miR-224 | *FOSB* | 2354 | FBJ murine osteosarcoma viral oncogene homolog B | Luciferase reporter assay |
| hsa-miR-224 | *KLK10* | 5655 | kallikrein-related peptidase 10 | qRT-PCR//Luciferase reporter assay |
| hsa-miR-224 | *NCOA6* | 23054 | nuclear receptor coactivator 6 | Luciferase reporter assay |
| hsa-miR-224 | *NIT1* | 4817 | nitrilase 1 | Luciferase reporter assay |
| hsa-miR-224 | *PDGFRB* | 5159 | platelet-derived growth factor receptor, beta polypeptide | Microarray//Northern blot |
| hsa-miR-224 | *RAB9B* | 51209 | RAB9B, member RAS oncogene family | Microarray//Northern blot |
| hsa-miR-224 | *DIO1* | 1733 | deiodinase, iodothyronine, type I | Luciferase reporter assay//qRT-PCR//Western blot |
| hsa-miR-224 | *SMAD4* | 4089 | SMAD family member 4 | Luciferase reporter assay//Western blot |
| hsa-miR-23a | *ATAT1* | 79969 | alpha tubulin acetyltransferase 1 | Luciferase reporter assay |
| hsa-miR-23a | *CXCL12* | 6387 | chemokine (C-x-C motif) ligand 12 | Luciferase reporter assay |
| hsa-miR-23a | *HES1* | 3280 | hairy and enhancer of split, (Drosophila) | Immunofluorescence//Luciferase reporter assay//Northern blot//Western blot |
| hsa-miR-23a | *IL6R* | 3570 | interleukin 6 receptor | GFP reporter assay//Microarray//qRT-PCR//Western blot |
| hsa-miR-23a | *POU4F2* | 5458 | POU class 4 homeobox 2 | Luciferase reporter assay |
| hsa-miR-23a | *FOXO3* | 2309 | forkhead box O3 | Immunoblot//Luciferase reporter assay//qRT-PCR |
| hsa-miR-23a | *G6PC* | 2538 | glucose-6-phosphatase, catalytic subunit | Luciferase reporter assay |
| hsa-miR-23a | *PPARGC1A* | 10891 | peroxisome proliferator-activated receptor gamma, coactivator 1 alpha | Luciferase reporter assay |
| hsa-miR-25 | *BCL2L11* | 10018 | BCL2-like 11 (apoptosis facilitator) | Luciferase reporter assay//Microarray//Western blot |
| hsa-miR-25 | *CDKN1C* | 1028 | cyclin-dependent kinase inhibitor 1C (p57, Kip2) | qRT-PCR//Luciferase reporter assay//Western blot |
| hsa-miR-25 | *KAT2B* | 8850 | K(lysine) acetyltransferase 2B | Western blot//Luciferase reporter assay |
| hsa-miR-25 | *KLF4* | 9314 | Kruppel-like factor 4 (gut) | Western blot |
| hsa-miR-25 | *PRMT5* | 10419 | protein arginine methyltransferase 5 | Western blot |
| hsa-miR-25 | *TP53* | 7157 | tumor protein p53 | Luciferase reporter assay//qRT-PCR//Western blot |
| hsa-miR-25 | *CCL26* | 10344 | chemokine (C-C motif) ligand 26 | Luciferase reporter assay//qRT-PCR//Western blot |
| hsa-miR-25 | *CDH1* | 999 | cadherin 1, type 1, E-cadherin (epithelial) | Luciferase reporter assay//qRT-PCR//Western blot |
| hsa-miR-25 | *WDR4* | 10785 | WD repeat domain 4 | Luciferase reporter assay//qRT-PCR//Western blot |
| hsa-miR-27a | *FOXO1* | 2308 | forkhead box O1 | Immunohistochemistry//Luciferase reporter assay//qRT-PCR//Western blot |
| hsa-miR-27a | *HIPK2* | 28996 | homeodomain interacting protein kinase 2 | qRT-PCR//Western blot |
| hsa-miR-27a | *MYT1* | 4661 | myelin transcription factor 1 | Western blot |
| hsa-miR-27a | *PHB* | 5245 | prohibitin | ChIP-seq//Luciferase reporter assay//Northern blot//qRT-PCR//Western blot |
| hsa-miR-27a | *SP1* | 6667 | Sp1 transcription factor | Western blot |
| hsa-miR-27a | *SP3* | 6670 | Sp3 transcription factor | Western blot |
| hsa-miR-27a | *SP4* | 6671 | Sp4 transcription factor | Western blot |
| hsa-miR-27a | *SPRY2* | 10253 | sprouty homolog 2 (Drosophila) | Immunohistochemistry//Luciferase reporter assay//Microarray//qRT-PCR//Western blot |
| hsa-miR-27a | *THRB* | 7068 | thyroid hormone receptor, beta | Luciferase reporter assay//Northern blot//Western blot |
| hsa-miR-27a | *ZBTB10* | 65986 | zinc finger and BTB domain containing 10 | Western blot |
| hsa-miR-27a | *APC* | 324 | adenomatous polyposis coli | Luciferase reporter assay//Western blot |
| hsa-miR-27a | *FBXW7* | 55294 | F-box and WD repeat domain containing 7, E3 ubiquitin protein ligase | Luciferase reporter assay//Western blot |
| hsa-miR-27a | *IGF1* | 3479 | insulin-like growth factor 1 (somatomedin C) | Luciferase reporter assay |
| hsa-miR-27a | *MMP13* | 4322 | matrix metallopeptidase 13 (collagenase) | qRT-PCR |
| hsa-miR-27a | *PAX3* | 5077 | paired box 3 | Luciferase reporter assay |
| hsa-miR-27a | *WEE1* | 7465 | WEE1 homolog (S.pombe) | GFP reporter assay//Luciferase reporter assay//qRT-PCR |
| hsa-miR-301 | *MEOX2* | 4223 | mesenchyme homeobox 2 | Luciferase reporter assay//qRT-PCR//Western blot |
| hsa-miR-301 | *NKRF* | 55922 | NFKB repressing factor | Luciferase reporter assay//Microarray//qRT-PCR//Western blot |
| hsa-miR-301 | *SERPINE1* | 5054 | serpin peptidase inhibitor, clade E (nexin, plasminogen activator inhibitor type 1), member 1 | Luciferase reporter assay |
| hsa-miR-335 | *ARPC5L* | 81873 | actin related protein 2/3 complex, subunit 5-like | Luciferase reporter assay |
| hsa-miR-335 | *MERTK* | 10461 | c-mer proto-oncogene tyrosine kinase | Luciferase reporter assay//Microarray |
| hsa-miR-335 | *PTPRN2* | 5799 | protein tyrosine phosphatase, receptor type, N polypeptide 2 | Luciferase reporter assay//Microarray |
| hsa-miR-335 | *RASA1* | 5921 | RAS p21 protein activator (GTPase activating protein) 1 | Luciferase reporter assay |
| hsa-miR-335 | *RB1* | 5925 | retinoblastoma 1 | Luciferase reporter assay//Western blot |
| hsa-miR-335 | *RUNX2* | 860 | runt-related transcription factor 2 | Luciferase reporter assay//Microarray//qRT-PCR//Western blot |
| hsa-miR-335 | *SOX4* | 6659 | SRY (sex determining region Y)-box 4 | Immunohistochemistry//Luciferase reporter assay//Microarray |
| hsa-miR-335 | *TNC* | 3371 | tenascin C | Immunohistochemistry//Luciferase reporter assay//Microarray |
| hsa-miR-335 | *UBE2F* | 140739 | ubiquitin-conjugating enzyme E2F (putative) | Luciferase reporter assay |
| hsa-miR-335 | *BCL2L2* | 599 | BCL2-like 2 | Luciferase reporter assay//qRT-PCR//Western blot |
| hsa-miR-335 | *LRG1* | 116844 | leucine-rich alpha-2-glycoprotein 1 | Immunofluorescence//Immunoprecipitaion//Luciferase reporter assay//Microarray//qRT-PCR//Western blot |
| hsa-miR-335 | *MAPK1* | 5594 | mitogen-activated protein kinase 1 | Immunofluorescence//Immunoprecipitaion//Luciferase reporter assay//Microarray//qRT-PCR//Western blot |
| hsa-miR-335 | *TFF2* | 7032 | trefoil factor 2 | Luciferase reporter assay//qRT-PCR//Western blot |
| hsa-miR-424 | *ANLN* | 54443 | anillin, actin binding protein | Luciferase reporter assay |
| hsa-miR-424 | *ATF6* | 22926 | activating transcription factor 6 | Luciferase reporter assay |
| hsa-miR-424 | *CCND1* | 595 | cyclin D1 | qRT-PCR//flow//Luciferase reporter assay//Western blot |
| hsa-miR-424 | *CCND3* | 896 | cyclin D3 | qRT-PCR//flow//Luciferase reporter assay//Western blot |
| hsa-miR-424 | *CCNE1* | 898 | cyclin E1 | qRT-PCR//flow//Western blot |
| hsa-miR-424 | *CCNF* | 899 | cyclin F | Luciferase reporter assay |
| hsa-miR-424 | *CDC14A* | 8556 | cell division cycle 14A | Luciferase reporter assay |
| hsa-miR-424 | *CDC25A* | 993 | cell division cycle 25A | Luciferase reporter assay |
| hsa-miR-424 | *CDK6* | 1021 | cyclin-dependent kinase 6 | qRT-PCR//flow//Luciferase reporter assay//Western blot |
| hsa-miR-424 | *CHEK1* | 1111 | checkpoint kinase 1 | Luciferase reporter assay |
| hsa-miR-424 | *CUL2* | 8453 | cullin 2 | ChIP-seq//Luciferase reporter assay//Microarray//qRT-PCR//Western blot |
| hsa-miR-424 | *FGFR1* | 2260 | fibroblast growth factor receptor 1 | Luciferase reporter assay |
| hsa-miR-424 | *HIF1A* | 3091 | hypoxia inducible factor 1, alpha subunit (basic helix-loop-helix transcription factor) | ChIP-seq//Luciferase reporter assay//Microarray//qRT-PCR//Western blot |
| hsa-miR-424 | *ITPR1* | 3708 | inositol 1,4,5-trisphosphate receptor, type 1 | Luciferase reporter assay |
| hsa-miR-424 | *KIF23* | 9493 | kinesin family member 23 | Luciferase reporter assay |
| hsa-miR-424 | *MAP2K1* | 5604 | mitogen-activated protein kinase kinase 1 | Luciferase reporter assay |
| hsa-miR-424 | *MYB* | 4602 | v-myb myeloblastosis viral oncogene homolog (avian) | Immunohistochemistry//Luciferase reporter assay//Northern blot//qRT-PCR//Western blot |
| hsa-miR-424 | *NFIA* | 4774 | nuclear factor I/A | Luciferase reporter assay//Northern blot//Western blot |
| hsa-miR-424 | *PIAS1* | 8554 | protein inhibitor of activated STAT, 1 | Luciferase reporter assay |
| hsa-miR-424 | *PLAG1* | 5324 | pleiomorphic adenoma gene 1 | Western blot//Luciferase reporter assay//Microarray |
| hsa-miR-424 | *SIAH1* | 6477 | siah E3 ubiquitin protein ligase 1 | Immunohistochemistry//Luciferase reporter assay//Northern blot//qRT-PCR//Western blot |
| hsa-miR-424 | *SPI1* | 6688 | spleen focus forming virus (SFFV) proviral integration oncogene spi1 | ChIP-seq//Luciferase reporter assay//Microarray//qRT-PCR//Western blot |
| hsa-miR-424 | *WEE1* | 7465 | WEE1 homolog (S.pombe) | Luciferase reporter assay |
| hsa-miR-519d | *AKT3* | 10000 | v-akt murine thymoma viral oncogene homolog 3 (protein kinase B, gamma) | Luciferase reporter assay//Western blot |
| hsa-miR-519d | *CDKN1A* | 1026 | cyclin-dependent kinase inhibitor 1A (p21, Cip1) | qRT-PCR//Luciferase reporter assay//Western blot |
| hsa-miR-519d | *PPARA* | 5465 | peroxisome proliferator-activated receptor alpha | Luciferase reporter assay//qRT-PCR//Western blot |
| hsa-miR-519d | *PTEN* | 5728 | phosphatase and tensin homolog | Luciferase reporter assay//Western blot |
| hsa-miR-519d | *TIMP2* | 7077 | TIMP metallopeptidase inhibitor 2 | Luciferase reporter assay//Western blot |
| hsa-miR-542-3p | *BIRC5* | 332 | baculoviral IAP repeat containing 5 | Luciferase reporter assay//Microarray//qRT-PCR//Western blot |
| hsa-miR-7 | *ABCC1* | 4363 | ATP-binding cassette, sub family C (CFTR/MRP), member 1 | Luciferase reporter assay//Western blot |
| hsa-miR-7 | *ARF4* | 378 | ADP-ribosylation fctor 4 | Microarray |
| hsa-miR-7 | *CAPZA1* | 829 | capping protein (actin filament) muscle Z-line, alpha 1 | Microarray |
| hsa-miR-7 | *CKAP4* | 10970 | cytoskeleton-associated protein 4 | Microarray |
| hsa-miR-7 | *CNN3* | 1266 | calponin 3, acidic | Microarray |
| hsa-miR-7 | *CNOT8* | 9337 | CCR4-NOT transcription complex, subunit 8 | Microarray |
| hsa-miR-7 | *EGFR* | 1956 | epidermal growth factor receptor | real-time RT-PCR//Reporter assay//Western blot |
| hsa-miR-7 | *HELLS* | 3070 | helicase, lymphoid-specific | Luciferase reporter assay |
| hsa-miR-7 | *IGF1R* | 3480 | insulin-like growth factor receptor | Flow//qRT-PCR//Western blot |
| hsa-miR-7 | *IRS1* | 3667 | insulin receptor substrate 1 | Western blot |
| hsa-miR-7 | *IRS2* | 8660 | insulin receptor substrate 2 | Reporter assay//Western blot |
| hsa-miR-7 | *PAK1* | 5058 | p21 protein (Cdc42/Rac)-activated kinase 1 | Luciferase reporter assay//Western blot |
| hsa-miR-7 | *PFN2* | 5217 | profilin 2 | Microarray |
| hsa-miR-7 | *PLEC* | 5339 | plectin | Microarray |
| hsa-miR-7 | *PSME3* | 10197 | proteasome (prosome, macropain) activator subunit 3 (PA28 gamma; Ki) | Microarray |
| hsa-miR-7 | *RAF1* | 5894 | v-raf-1 murine leukemia viral oncogene homolog 1 | Luciferase reporter assay//Microarray//qRT-PCR//Western blot |
| hsa-miR-7 | *SLC7A5* | 8140 | solute carrier family 7 (amino acid transporter light chain, L system), member 5 | Immunofluorescence//Luciferase reporter assay//qRT-PCR//Western blot//Immunoprecipitation |
| hsa-miR-7 | *SNCA* | 6622 | synuclein, alpha (non A4 component of amyloid precursor) | qRT-PCR//Luciferase reporter assay//Western blot |
| hsa-miR-7 | *SRSF1* | 6426 | serine/arginine-rich splicing factor 1 | qRT-PCR//Luciferase reporter assay//Western blot//Northern blot |
| hsa-miR-92 | *ARID4B* | 51742 | AT rich interactive domain 4B (RBP1-like) | Luciferase reporter assay |
| hsa-miR-92 | *BMPR2* | 659 | bone morphogenetic protein receptor, type II (serine/threonine kinase) | Luciferase reporter assay//qRT-PCR//Western blot |
| hsa-miR-92 | *CPEB2* | 132864 | cytoplasmic polyadenylation element binding protein 2 | Luciferase reporter assay//Northern blot//qRT-PCR |
| hsa-miR-92 | *ESR2* | 2100 | estrogen receptor 2 (ER beta) | GFP reporter assay |
| hsa-miR-92 | *HIPK3* | 10114 | homeodomain interacting protein kinase 3 | Luciferase reporter assay |
| hsa-miR-92 | *ITGA5* | 3678 | integrin, alpha 5 (fibronectin receptor, alpha polypeptide) | Luciferase reporter assay//Western blot//Microarray |
| hsa-miR-92 | *KAT2B* | 8850 | K(lysine) acetyltransferase 2B | Western blot//Luciferase reporter assay |
| hsa-miR-92 | *MYLIP* | 29116 | myosin regulatory light chain interacting protein | Luciferase reporter assay |
| hsa-miR-92 | *SMAD4* | 4089 | SMAD family member 4 | Microarray |
| hsa-miR-92 | *TGFBR2* | 7048 | transforming growth factor, beta receptor II (70/80kDa) | Microarray//qRT-PCR |
| hsa-miR-92 | *THBS1* | 7057 | thrombospondin 1 | Microarray//qRT-PCR |
| hsa-miR-92 | *TP63* | 8626 | tumor protein p63 | Luciferase reporter assay//qRT-PCR//Western blot |
| hsa-miR-92 | *BCL2L11* | 10018 | BCL2-like 11 (apoptosis facilitator) | qRT-PCR//Western blot |
| hsa-miR-92 | *CDH1* | 999 | cadherin 1, type 1, E-cadherin (epithelial) | Luciferase reporter assay//Northern blot//qRT-PCR |
| hsa-miR-92 | *KLF2* | 10365 | Kruppel-like factor 2 (lung) | Luciferase reporter assay |
| hsa-miR-93 | *CDKN1A* | 1026 | cyclin-dependent kinase inhibitor 1A (p21, Cip1) | Luciferase reporter assay//Microarray//Western blot |
| hsa-miR-93 | *E2F1* | 1869 | E2F transcription factor 1 | Luciferase reporter assay//Western blot |
| hsa-miR-93 | *ITGB8* | 3696 | integrin, beta 8 | Luciferase reporter assay//qRT-PCR//Western blot |
| hsa-miR-93 | *KAT2B* | 8850 | K(lysine) acetyltransferase 2B | Western blot//Luciferase reporter assay |
| hsa-miR-93 | *MAPK9* | 5601 | mitogen-activated protein kinase 9 | Luciferase reporter assay |
| hsa-miR-93 | *TP53INP1* | 94241 | tumor protein p53 inducible nuclear protein 1 | Luciferase reporter assay//Western blot |
| hsa-miR-93 | *TUSC2* | 11334 | tumor suppressor candidate 2 | Luciferase reporter assay//Western blot |
| hsa-miR-93 | *VEGFA* | 7422 | vascular endothelial growth factor A | ELISA//Luciferase reporter assay |
| hsa-miR-93 | *PTEN* | 5728 | phosphatase and tensin homolog | GFP reporter assay//qRT-PCR//Western blot |
| hsa-miR-99b | *RAVER2* | 55225 | ribonucleoprotein, PTB-binding 2 | Immunoprecipitaion//Luciferase reporter assay//Western blot |
| hsa-miR-99b | *MTOR* | 2475 | mechanistic target of rapamycin (serine/threonine kinase) | FACS//Luciferase reporter assay//qRT-PCR//Western blot |

List of experimentally validated targets from miRTarBase for miRNAs upregulated in gastric cancer. Total 675 redundant target genes with 489 non-redundant target genes are listed with MicroRNA, Target gene, Entrez gene ID, Gene name and Validation method. Abbreviation: ChiP: chromatin immunoprecipitation, ELISA: enzyme-linked immunosorbent assay, FACS: fluorescence-activated cell sorting, GFP: green fluorescent protein, qRT-PCR: quantitative RT-PCR

**Supplementary Table S4.** Experimentally validated target genes for microRNAs up- and down-regulated in gastric cancer

| **MicroRNA** | **Target gene** | **Entrez gene ID** | **Gene name** | **Validation method** |
| --- | --- | --- | --- | --- |
| hsa-let-7a | *AMMECR1* | 9949 | Alport syndrome, mental retardation, midface hypoplasia and elliptocytosis chromosomal region gene 1 | qRT-PCR |
| hsa-let-7a | *APP* | 351 | amyloid beta (A4) precursor protein | Luciferase reporter assay |
| hsa-let-7a | *BCL2* | 596 | B-cell CLL/lymphoma 2 | Microarray//qRT-PCR |
| hsa-let-7a | *CASP3* | 836 | caspase 3, apoptosis-related cysteine peptidase | Luciferase reporter assay//Western blot |
| hsa-let-7a | *CASP8* | 841 | caspase 8, apoptosis-related cysteine peptidase | Western blot |
| hsa-let-7a | *CASP9* | 842 | caspase 9, apoptosis-related cysteine peptidase | Western blot |
| hsa-let-7a | *CCND2* | 894 | cyclin D2 | Luciferase reporter assay//qRT-PCR//Western blot |
| hsa-let-7a | *DICER1* | 23405 | dicer 1, ribonuclease type III | Luciferase reporter assay//Western blot//Northern blot |
| hsa-let-7a | *E2F1* | 1869 | E2F transcription factor 1 | Western blot |
| hsa-let-7a | *E2F2* | 1870 | E2F transcription factor 2 | Luciferase reporter assay//qRT-PCR//Western blot |
| hsa-let-7a | *EGR3* | 1960 | early growth response 3 | qRT-PCR |
| hsa-let-7a | *EIF2C4* | 192670 | argonaute RISC catalytic component 4 | Luciferase reporter assay |
| hsa-let-7a | *FOXA1* | 3169 | forkhead box A1 | Luciferase reporter assay |
| hsa-let-7a | *HMGA1* | 3159 | high mobility group AT-hook 1 | Luciferase reporter assay//qRT-PCR |
| hsa-let-7a | *HMGA2* | 8091 | high mobility group AT-hook 2 | qRT-PCR//Western blot |
| hsa-let-7a | *HNRPDL* | 9987 | heterogeneous nuclear ribonucleoprotein D-like | qRT-PCR |
| hsa-let-7a | *HRAS* | 3265 | v-Ha-ras Harvey rat sarcoma viral oncogene homolog | Immunoblot//Luciferase assay//qRT-PCR//Western blot |
| hsa-let-7a | *IGF2* | 3481 | insulin-like growth factor 2 (somatomedin A) | Luciferase reporter assay |
| hsa-let-7a | *IL6* | 3569 | interleukin 6 (interferon, beta 2) | Luciferase reporter assay//qRT-PCR//Western blot |
| hsa-let-7a | *ITGB3* | 3690 | integrin, beta 3 (platelet glycoprotein IIIa, antigen CD61) | qRT-PCR//Luciferase reporter assay//Western blot |
| hsa-let-7a | *KRAS* | 3845 | v-Ki-ras2 Kirsten rat sarcoma viral oncogene homolog | Luciferase reporter assay//Microarray |
| hsa-let-7a | *LIN28A* | 79727 | lin-28 homolog A (C. elegans) | B-globin reporter assay//Luciferase reporter assay//Microarray |
| hsa-let-7a | *MEIS1* | 4211 | Meis homeobox 1 | qRT-PCR |
| hsa-let-7a | *MYC* | 4609 | v-myc myelocytomatosis viral oncogene homolog (avian) | Western blot |
| hsa-let-7a | *NEFM* | 4741 | neurofilament, medium polypeptide | qRT-PCR |
| hsa-let-7a | *NF2* | 4771 | neurofibromin 2 (merlin) | Luciferase reporter assay//Microarray//qRT-PCR//Western blot |
| hsa-let-7a | *NFKB1* | 4790 | nuclear factor of kappa light polypeptide gene enhancer in B-cells 1 | Microarray//qRT-PCR |
| hsa-let-7a | *NKIRAS2* | 28511 | NFKB inhibitor interacting Ras-like 2 | ELISA//qRT-PCR//Luciferase reporter assay//Western blot |
| hsa-let-7a | *NR1I2* | 8856 | nuclear receptor subfamily 1, group I, member 2 | Luciferase reporter assay |
| hsa-let-7a | *NRAS* | 4893 | neuroblastoma RAS viral (v-ras) oncogene homolog | Western blot |
| hsa-let-7a | *PRDM1* | 639 | PR domain containing 1, with ZNF domain | Luciferase reporter assay |
| hsa-let-7a | *RAVER2* | 55225 | ribonucleoprotein, PTB-binding 2 | Immunoprecipitaion//Luciferase reporter assay//Western blot |
| hsa-let-7a | *SLC20A1* | 6574 | solute carrier family 20 (phosphate transporter), member 1 | qRT-PCR |
| hsa-let-7a | *THBS1* | 7057 | thrombospondin 1 | qRT-PCR |
| hsa-let-7a | *TRIM71* | 131405 | tripartite motif containing 71, E3 ubiquitin protein ligase | Luciferase reporter assay |
| hsa-let-7a | *TUSC2* | 11334 | tumor suppressor candidate 2 | qRT-PCR |
| hsa-let-7a | *UHRF2* | 115426 | ubiquitin-like with PHD and ring finger domains 2, E3 ubiquitin protein ligase | qRT-PCR//Western blot |
| hsa-let-7a | *VDR* | 7421 | vitamin D (1,25- dihydroxyvitamin D3) receptor | Luciferase reporter assay |
| hsa-let-7a | *ZFP36L1* | 677 | ZFP36 ring finger protein-like 1 | qRT-PCR |
| hsa-let-7a | *CDC34* | 997 | cell division cycle 34 | Immunoblot//qRT-PCR//Western blot |
| hsa-let-7a | *EWSR1* | 2130 | EWS RNA-binding protein 1 | Luciferase reporter assay//Microarray//qRT-PCR//Western blot |
| hsa-let-7a | *IGF2BP1* | 10642 | insulin-like growth factor 2 mRNA binding protein 1 | Immunoblot//qRT-PCR//Western blot |
| hsa-let-7a | *MPL* | 4352 | myeloproliferative leukemia virus oncogene | Luciferase reporter assay |
| hsa-let-7d | *APP* | 351 | amyloid beta (A4) precursor protein | Luciferase reporter assay |
| hsa-let-7d | *DICER1* | 23405 | dicer 1, ribonuclease type III | Luciferase reporter assay//Western blot//Northern blot |
| hsa-let-7d | *HMGA2* | 8091 | high mobility group AT-hook 2 | Luciferase reporter assay |
| hsa-let-7d | *PDGFA* | 5154 | platelet-derived growth factor alpha polypeptide | ELISA//Immunoblot//Microarray//qRT-PCR |
| hsa-let-7d | *SLC11A2* | 4891 | solute carrier family 11 (proton-coupled divalent metal ion transporters), member 2 | qRT-PCR//Luciferase reporter assay//Western blot |
| hsa-let-7d | *IL13* | 3596 | interleukin 13 | Luciferase reporter assay |
| hsa-let-7d | *MPL* | 4352 | myeloproliferative leukemia virus oncogene | Luciferase reporter assay |
| hsa-let-7e | *EIF3J* | 8669 | eukaryotic translation initiation factor 3, subunit J | Luciferase reporter assay |
| hsa-let-7e | *HMGA2* | 8091 | high mobility group AT-hook 2 | Luciferase reporter assay//qRT-PCR |
| hsa-let-7e | *SMC1A* | 8243 | structural maintenance of chromosomes 1A | Luciferase reporter assay |
| hsa-let-7e | *WNT1* | 7471 | wingless-type MMTV integration site family, member 1 | Flow//Immunoblot//Luciferase reporter assay//Microarray//qRT-PCR |
| hsa-let-7e | *CCND1* | 595 | cyclin D1 | Luciferase reporter assay//qRT-PCR//Western blot |
| hsa-let-7f | *KLK10* | 5655 | kallikrein-related peptidase 10 | qRT-PCR//Luciferase reporter assay |
| hsa-let-7f | *KLK6* | 5653 | kallikrein-related peptidase 6 | ELISA//Luciferase reporter assay//qRT-PCR |
| hsa-let-7f | *PRDM1* | 639 | PR domain containing 1, with ZNF domain | Immunohistochemistry//Luciferase reporter assay//qRT-PCR//Western blot |
| hsa-let-7f | *IL13* | 3596 | interleukin 13 | Luciferase reporter assay |
| hsa-let-7f | *MPL* | 4352 | myeloproliferative leukemia virus oncogene | Luciferase reporter assay |
| hsa-let-7g | *BCL2L1* | 598 | BCL2-like 1 | Luciferase reporter assay//qRT-PCR//Western blot |
| hsa-let-7g | *CDKN2A* | 1029 | cyclin-dependent kinase inhibitor 2A | qRT-PCR//Luciferase reporter assay//Western blot |
| hsa-let-7g | *COL1A2* | 1278 | collagen, type I, alpha 2 | qRT-PCR//Luciferase reporter assay//Western blot |
| hsa-let-7g | *HMGA2* | 8091 | high mobility group AT-hook 2 | Western blot |
| hsa-let-7g | *IGF2BP1* | 10642 | insulin-like growth factor 2 mRNA binding protein 1 | Western blot |
| hsa-let-7g | *KRAS* | 3845 | v-Ki-ras2 Kirsten rat sarcoma viral oncogene homolog | Luciferase reporter assay |
| hsa-let-7g | *MYC* | 4609 | v-myc myelocytomatosis viral oncogene homolog (avian) | qRT-PCR//Luciferase reporter assay//Western blot |
| hsa-let-7g | *FN1* | 2335 | fibronectin 1 | Luciferase reporter assay//Microarray//Western blot |
| hsa-let-7g | *GAB2* | 9846 | GRB2-associated binding protein 2 | Luciferase reporter assay//Microarray//Western blot |
| hsa-let-7g | *IL13* | 3596 | interleukin 13 | Luciferase reporter assay |
| hsa-miR-103 | *CCNE1* | 898 | cyclin E1 | Luciferase reporter assay//qRT-PCR//Western blot |
| hsa-miR-103 | *CDK2* | 1017 | cyclin-dependent kinase 2 | Luciferase reporter assay//qRT-PCR//Western blot |
| hsa-miR-103 | *CREB1* | 1385 | cAMP responsive element binding protein 1 | Luciferase reporter assay//qRT-PCR//Western blot |
| hsa-miR-103 | *DICER1* | 23405 | dicer 1, ribonuclease type III | Luciferase reporter assay//qRT-PCR |
| hsa-miR-103 | *GPD1* | 2819 | glycerol-3-phosphate dehydrogenase 1 (soluble) | Luciferase reporter assay |
| hsa-miR-103 | *CAV1* | 857 | caveolin 1, caveolae protein, 22kDa | Luciferase reporter assay//Microarray |
| hsa-miR-107 | *ARNT* | Target | aryl hydrocarbon receptor nuclear translocator | qRT-PCR//Luciferase reporter assay//Western blot//Northern blot |
| hsa-miR-107 | *BACE1* | 23621 | beta-site APP-cleaving enzyme 1 | Western blot//Northern blot |
| hsa-miR-107 | *CCNE1* | 898 | cyclin E1 | Flow//Immunoblot//Microarray//qRT-PCR |
| hsa-miR-107 | *CDCA4* | 55038 | cell division cycle associated 4 | Flow//Immunoblot//Microarray//qRT-PCR |
| hsa-miR-107 | *CDK6* | 1021 | cyclin-dependent kinase 6 | Western blot |
| hsa-miR-107 | *CRKL* | 1399 | v-crk sarcoma virus CT10 oncogene homolog (avian)-like | Flow//Immunoblot//Microarray//qRT-PCR |
| hsa-miR-107 | *DICER1* | 23405 | dicer 1, ribonuclease type III | Luciferase reporter assay//qRT-PCR |
| hsa-miR-107 | *FBXW7* | 55294 | F-box and WD repeat domain containing 7, E3 ubiquitin protein ligase | Luciferase reporter assay//Microarray//Northern blot//Western blot |
| hsa-miR-107 | *GRN* | 2896 | granulin | Microarray//Western blot |
| hsa-miR-107 | *HIF1A* | 3091 | hypoxia inducible factor 1, alpha subunit (basic helix-loop-helix transcription factor) | qRT-PCR//Luciferase reporter assay//Western blot//Northern blot |
| hsa-miR-107 | *MYB* | 4602 | v-myb myeloblastosis viral oncogene homolog (avian) | Luciferase reporter assay//Western blot |
| hsa-miR-107 | *NFIA* | 4774 | nuclear factor I/A | Luciferase reporter assay |
| hsa-miR-107 | *PLAG1* | 5324 | pleiomorphic adenoma gene 1 | Luciferase reporter assay |
| hsa-miR-107 | *RAB1B* | 81876 | RAB1B, member RAS oncogene family | Flow//Immunoblot//Microarray//qRT-PCR |
| hsa-miR-107 | *VEGFA* | 7422 | vascular endothelial growth factor A | ELISA//Luciferase reporter assay |
| hsa-miR-125a | *ARID3B* | 10620 | AT rich interactive domain 3B (BRIGHT-like) | Immunofluorescence//qRT-PCR//Western blot |
| hsa-miR-125a | *BAK1* | 578 | BCL2-antagonist/killer 1 | Flow//Luciferase reporter assay//qRT-PCR//Western blot |
| hsa-miR-125a | *CCL5* | 6352 | chemokine (C-C motif) ligand 5 | ELISA |
| hsa-miR-125a | *CD34* | 947 | CD34 molecule | Luciferase reporter assay//Microarray//Western blot |
| hsa-miR-125a | *CDKN1A* | 1026 | cyclin-dependent kinase inhibitor 1A (p21, Cip1) | qRT-PCR//Luciferase reporter assay//Western blot |
| hsa-miR-125a | *ELAVL1* | 1994 | ELAV (embryonic lethal, abnormal vision, Drosophila)-like 1 (Hu antigen R) | Luciferase reporter assay//Western blot |
| hsa-miR-125a | *ERBB2* | 2064 | v-erb-b2 erythroblastic leukemia viral oncogene homolog 2, neuro/glioblastoma derived oncogene homolog (avian) | Luciferase reporter assay//Northern blot//qRT-PCR//Western blot |
| hsa-miR-125a | *ERBB3* | 2065 | v-erb-b2 erythroblastic leukemia viral oncogene homolog 3 (avian) | Luciferase reporter assay//Northern blot//qRT-PCR//Western blot |
| hsa-miR-125a | *KLF13* | 51621 | Kruppel-like factor 13 | ELISA//Luciferase reporter assay//qRT-PCR//Western blot |
| hsa-miR-125a | *LIF* | 3976 | leukemia inhibitory factor | ELISA |
| hsa-miR-125a | *LIN28A* | 79727 | lin-28 homolog A (C. elegans) | Luciferase reporter assay |
| hsa-miR-125a | *MTUS1* | 57509 | microtubule associated tumor suppressor 1 | Microarray//Northern blot |
| hsa-miR-125a | *NTRK3* | 4916 | neurotrophic tyrosine kinase, receptor, type 3 | Luciferase reporter assay//Western blot |
| hsa-miR-125a | *RARA* | 5914 | retinoic acid receptor, alpha | Microarray//Northern blot |
| hsa-miR-125a | *TNFAIP3* | 7128 | tumor necrosis factor, alpha-induced protein 3 | qRT-PCR//Western blot |
| hsa-miR-125a | *TP53* | 7157 | tumor protein p53 | Luciferase reporter assay//Northern blot//qRT-PCR//Western blot |
| hsa-miR-125a | *VEGFA* | 7422 | vascular endothelial growth factor A | ELISA//Luciferase reporter assay |
| hsa-miR-125b | *ABCC4* | 10257 | ATP-binding cassette, sub-family C (CFTR/MRP), member 4 | Microarray |
| hsa-miR-125b | *AKT1* | 207 | v-akt murine thymoma viral oncogene homolog 1 | qRT-PCR//Western blot |
| hsa-miR-125b | *ATXN1* | 6310 | ataxin 1 | Microarray |
| hsa-miR-125b | *BAK1* | 578 | BCL2-antagonist/killer 1 | Luciferase reporter assay//qRT-PCR//Western blot |
| hsa-miR-125b | *BBC3* | 27113 | BCL2 binding component 3 | Luciferase reporter assay//qRT-PCR//Western blot |
| hsa-miR-125b | *BMF* | 90427 | Bcl2 modifying factor | immunoblot//Luciferase reporter assay//qRT-PCR |
| hsa-miR-125b | *BMPR1B* | 658 | bone morphogenetic protein receptor, type IB | Luciferase reporter assay |
| hsa-miR-125b | *CASC3* | 22794 | cancer susceptibility candidate 3 | Microarray |
| hsa-miR-125b | *CBFB* | 865 | core-binding factor, beta subunit | Luciferase reporter assay//microarray//qRT-PCR//Western blot |
| hsa-miR-125b | *CDKN2A* | 1029 | cyclin-dependent kinase inhibitor 2A | Western blot |
| hsa-miR-125b | *CGN* | 57530 | cingulin | Microarray |
| hsa-miR-125b | *CYP24A1* | 1591 | cytochrome P450, family 24, subfamily A, polypeptide 1 | qRT-PCR//Luciferase reporter assay//Western blot//Northern blot |
| hsa-miR-125b | *E2F3* | 1871 | E2F transcription factor 3 | Immunohistochemistry//Luciferase reporter assay//Northern blot//qRT-PCR//Western blot |
| hsa-miR-125b | *EIF4EBP1* | 1978 | eukaryotic translation initiation factor 4E binding protein 1 | Microarray//qRT-PCR |
| hsa-miR-125b | *ERBB2* | 2064 | v-erb-b2 erythroblastic leukemia viral oncogene homolog 2, neuro/glioblastoma derived oncogene homolog (avian) | Western blot |
| hsa-miR-125b | *ERBB3* | 2065 | v-erb-b2 erythroblastic leukemia viral oncogene homolog 3 (avian) | Luciferase reporter assay//Northern blot//qRT-PCR//Western blot |
| hsa-miR-125b | *GLI1* | 2735 | GLI family zinc finger 1 | Luciferase reporter assay |
| hsa-miR-125b | *GRIN2A* | 2903 | glutamate receptor, ionotropic, N-methyl D-aspartate 2A | Luciferase reporter assay//qRT-PCR |
| hsa-miR-125b | *HMGA1* | 3159 | high mobility group AT-hook 1 | Luciferase reporter assay |
| hsa-miR-125b | *HMGA2* | 8091 | high mobility group AT-hook 2 | Luciferase reporter assay |
| hsa-miR-125b | *IGF2* | 3481 | insulin-like growth factor 2 (somatomedin A) | Luciferase reporter assay//Northern blot//Western blot |
| hsa-miR-125b | *IRF4* | 3662 | interferon regulatory factor 4 | qRT-PCR//Luciferase reporter assay |
| hsa-miR-125b | *KCNS3* | 3790 | potassium voltage-gated channel, delayed-rectifier, subfamily S, member 3 | Microarray |
| hsa-miR-125b | *KLF13* | 51621 | Kruppel-like factor 13 | Microarray//qRT-PCR//Western blot |
| hsa-miR-125b | *KRT7* | 3855 | keratin 7 | Immunoblot//qRT-PCR |
| hsa-miR-125b | *LACTB* | 114294 | lactamase, beta | Microarray |
| hsa-miR-125b | *LIF* | 3976 | leukemia inhibitory factor | ELISA |
| hsa-miR-125b | *LIN28A* | 79727 | lin-28 homolog A (C. elegans) | Immunoblot//Luciferase reporter assay//qRT-PCR |
| hsa-miR-125b | *LIN28B* | 389421 | lin-28 homolog B (C. elegans) | Luciferase reporter assay//qRT-PCR//Western blot |
| hsa-miR-125b | *LYPLA2* | 11313 | lysophospholipase II | Microarray |
| hsa-miR-125b | *MKNK2* | 2872 | MAP kinase interacting serine/threonine kinase 2 | Microarray |
| hsa-miR-125b | *NKIRAS2* | 28511 | NFKB inhibitor interacting Ras-like 2 | ELISA//qRT-PCR//Luciferase reporter assay//Western blot |
| hsa-miR-125b | *NTRK3* | 4916 | neurotrophic tyrosine kinase, receptor, type 3 | Luciferase reporter assay//Western blot |
| hsa-miR-125b | *PABPC1* | 26986 | poly(A) binding protein, cytoplasmic 1 | Microarray |
| hsa-miR-125b | *PLEKHA8* | 84725 | pleckstrin homology domain containing, family A (phosphoinositide binding specific) member 8 | Microarray |
| hsa-miR-125b | *PPAT* | 5471 | phosphoribosyl pyrophosphate amidotransferase | Microarray |
| hsa-miR-125b | *PRDM1* | 639 | PR domain containing 1, with ZNF domain | qRT-PCR//Luciferase reporter assay |
| hsa-miR-125b | *QSOX2* | 169714 | quiescin Q6 sulfhydryl oxidase 2 | Microarray |
| hsa-miR-125b | *RAF1* | 5894 | v-raf-1 murine leukemia viral oncogene homolog 1 | Western blot |
| hsa-miR-125b | *RNF144A* | 9781 | ring finger protein 144A | Microarray |
| hsa-miR-125b | *RPL29* | 6159 | ribosomal protein L29 | Microarray |
| hsa-miR-125b | *SAMD10* | 140700 | sterile alpha motif domain containing 10 | Microarray |
| hsa-miR-125b | *SEL1L* | 6400 | sel-1 suppressor of lin-12-like (C. elegans) | Microarray |
| hsa-miR-125b | *SLC35A4* | 113829 | solute carrier family 35, member A4 | Microarray |
| hsa-miR-125b | *SLC7A1* | 6541 | solute carrier family 7 (cationic amino acid transporter, y+ system), member 1 | Microarray |
| hsa-miR-125b | *SLC7A6* | 9057 | solute carrier family 7 (amino acid transporter light chain, y+L system), member 6 | Microarray |
| hsa-miR-125b | *SMARCD2* | 6603 | SWI/SNF related, matrix associated, actin dependent regulator of chromatin, subfamily d, member 2 | Microarray |
| hsa-miR-125b | *SMO* | 6608 | smoothened, frizzled family receptor | Luciferase reporter assay//Western blot |
| hsa-miR-125b | *STAT3* | 6774 | signal transducer and activator of transcription 3 (acute-phase response factor) | Microarray |
| hsa-miR-125b | *TP53* | 7157 | tumor protein p53 | Luciferase reporter assay//qRT-PCR//Western blot |
| hsa-miR-125b | *TP53INP1* | 94241 | tumor protein p53 inducible nuclear protein 1 | Luciferase reporter assay//Northern blot//Microarray//qRT-PCR//Western blot |
| hsa-miR-125b | *ULK3* | 25989 | unc-51-like kinase 3 (C. elegans) | Microarray |
| hsa-miR-125b | *VDR* | 7421 | vitamin D (1,25- dihydroxyvitamin D3) receptor | Luciferase reporter assay//Western blot |
| hsa-miR-125b | *BCL2* | 596 | B-cell CLL/lymphoma 2 | Luciferase reporter assay//Western blot |
| hsa-miR-125b | *BCL3* | 602 | B-cell CLL/lymphoma 3 | Luciferase reporter assay |
| hsa-miR-125b | *DGAT1* | 8694 | diacylglycerol O-acyltransferase 1 | Luciferase reporter assay |
| hsa-miR-125b | *ETS1* | 2113 | v-ets erythroblastosis virus E26 oncogene homolog 1 (avian) | Immunoblot//Luciferase reporter assay |
| hsa-miR-125b | *PIGF* | 5281 | phosphatidylinositol glycan anchor biosynthesis, class F | Luciferase reporter assay//qRT-PCR//Western blot |
| hsa-miR-125b | *PPP1CA* | 5499 | protein phosphatase 1, catalytic subunit, alpha isozyme | Luciferase reporter assay//qRT-PCR//Western blot |
| hsa-miR-125b | *PRKRA* | 8575 | protein kinase, interferon-inducible double stranded RNA dependent activator | Luciferase reporter assay//qRT-PCR//Western blot |
| hsa-miR-125b | *RPS6KA1* | 6195 | ribosomal protein S6 kinase, 90kDa, polypeptide 1 | Luciferase reporter assay |
| hsa-miR-125b | *SGPL1* | 8879 | sphingosine-1-phosphate lyase 1 | Luciferase reporter assay |
| hsa-miR-125b | *TBC1D1* | 23216 | TBC1 (tre-2/USP6, BUB2, cdc16) domain family, member 1 | Luciferase reporter assay |
| hsa-miR-125b | *TNFAIP3* | 7128 | tumor necrosis factor, alpha-induced protein 3 | qRT-PCR//Western blot |
| hsa-miR-138 | *ARHGEF3* | 50650 | Rho guanine nucleotide exchange factor (GEF) 3 | qRT-PCR//Luciferase reporter assay//Western blot |
| hsa-miR-138 | *CEBPA* | 1050 | CCAAT/enhancer binding protein (C/EBP), alpha | qRT-PCR |
| hsa-miR-138 | *EID1* | 23741 | EP300 interacting inhibitor of differentiation 1 | Luciferase reporter assay//qRT-PCR//Western blot//Quantitative proteomic approach |
| hsa-miR-138 | *FABP4* | 2167 | fatty acid binding protein 4, adipocyte | qRT-PCR |
| hsa-miR-138 | *IGF1R* | 3480 | insulin-like growth factor 1 receptor | Luciferase reporter assay |
| hsa-miR-138 | *LPL* | 4023 | lipoprotein lipase | qRT-PCR |
| hsa-miR-138 | *PPARG* | 5468 | peroxisome proliferator-activated receptor gamma | qRT-PCR |
| hsa-miR-138 | *RHOC* | 389 | ras homolog family member C | qRT-PCR//Luciferase reporter assay//Western blot |
| hsa-miR-138 | *ROCK2* | 9475 | Rho-associated, coiled-coil containing protein kinase 2 | qRT-PCR//Luciferase reporter assay//Western blot |
| hsa-miR-138 | *SLC45A3* | 85414 | solute carrier family 45, member 3 | Western blot//Luciferase reporter assay |
| hsa-miR-138 | *TERT* | 7015 | telomerase reverse transcriptase | qRT-PCR//Luciferase reporter assay//Western blot |
| hsa-miR-138 | *CCND3* | 896 | cyclin D3 | Luciferase reporter assay//qRT-PCR//Western blot |
| hsa-miR-138 | *FOSL1* | 8061 | FOS-like antigen 1 | Luciferase reporter assay//Western blot |
| hsa-miR-138 | *GNAI2* | 2771 | guanine nucleotide binding protein (G protein), alpha inhibiting activity polypeptide 2 | Microarray//qRT-PCR//Western blot |
| hsa-miR-138 | *H2AFX* | 3014 | H2A histone family, member X | Luciferase reporter assay//Western blot |
| hsa-miR-138 | *PTK2* | 5747 | protein tyrosine kinase 2 | Luciferase reporter assay//Western blot |
| hsa-miR-138 | *SIRT1* | 23411 | sirtuin 1 | Luciferase reporter assay |
| hsa-miR-141 | *BAP1* | 8314 | BRCA1 associated protein-1 (ubiquitin carboxy-terminal hydrolase) | Microarray |
| hsa-miR-141 | *BRD3* | 8019 | bromodomain containing 3 | Luciferase reporter assay//Western blot |
| hsa-miR-141 | *CLOCK* | 9575 | clock circadian regulator | Luciferase reporter assay |
| hsa-miR-141 | *DLX5* | 1749 | distal-less homeobox 5 | Luciferase reporter assay |
| hsa-miR-141 | *EIF4E* | 1977 | eukaryotic translation initiation factor 4E | Luciferase reporter assay//Northern blot//qRT-PCR//Western blot |
| hsa-miR-141 | *KLF5* | 688 | Kruppel-like factor 5 (intestinal) | Luciferase reporter assay |
| hsa-miR-141 | *PTEN* | 5728 | phosphatase and tensin homolog | Luciferase reporter assay//Western blot |
| hsa-miR-141 | *SFPQ* | 6421 | splicing factor proline/glutamine-rich | Immunoprecipitaion//Luciferase reporter assay//Western blot |
| hsa-miR-141 | *SIP1* | 8487 | gem (nuclear organelle) associated protein 2 | Luciferase reporter assay//qRT-PCR//Western blot |
| hsa-miR-141 | *STK3* | 6788 | serine/threonine kinase 3 | Luciferase reporter assay |
| hsa-miR-141 | *TGFB2* | 7042 | transforming growth factor, beta 2 | Immunoblot//Luciferase reporter assay//qRT-PCR |
| hsa-miR-141 | *TRAPPC2P1* | 10597 | trafficking protein particle complex 2 pseudogene 1 | Luciferase reporter assay |
| hsa-miR-141 | *UBAP1* | 51271 | ubiquitin associated protein 1 | Luciferase reporter assay//Western blot |
| hsa-miR-141 | *ZEB1* | 6935 | zinc finger E-box binding homeobox 1 | Luciferase reporter assay//qRT-PCR |
| hsa-miR-141 | *ZEB2* | 9839 | zinc finger E-box binding homeobox 2 | qRT-PCR |
| hsa-miR-141 | *ZFPM2* | 23414 | zinc finger protein, FOG family member 2 | Luciferase reporter assay//Western blot |
| hsa-miR-141 | *ACVR2B* | 93 | activin A receptor, type IIB | Luciferase reporter assay//Western blot |
| hsa-miR-141 | *CDYL* | 9425 | chromodomain protein, Y-like | Luciferase reporter assay//Western blot |
| hsa-miR-141 | *CTBP2* | 1488 | C-terminal binding protein 2 | Luciferase reporter assay//Western blot |
| hsa-miR-141 | *MAPK14* | 1432 | mitogen-activated protein kinase 14 | Luciferase reporter assay//Microarray//Western blot |
| hsa-miR-143 | *COL1A1* | 1277 | collagen, type I, alpha 1 | Immunoblot//Luciferase reporter assay//qRT-PCR |
| hsa-miR-143 | *COX2* | 4513 | mitochondrially encoded cytochrome c oxidase II | Microarray |
| hsa-miR-143 | *DNMT3A* | 1788 | DNA (cytosine-5-)-methyltransferase 3 alpha | qRT-PCR//Luciferase reporter assay//Western blot |
| hsa-miR-143 | *FNDC3B* | 64778 | fibronectin type III domain containing 3B | Luciferase reporter assay//qRT-PCR//Western blot |
| hsa-miR-143 | *FSCN1* | 6624 | fascin homolog 1, actin-bundling protein (Strongylocentrotus purpuratus) | Luciferase reporter assay//qRT-PCR//Western blot |
| hsa-miR-143 | *HRAS* | 3265 | v-Ha-ras Harvey rat sarcoma viral oncogene homolog | Luciferase reporter assay//Northern blot//qRT-PCR |
| hsa-miR-143 | *KRAS* | 3845 | v-Ki-ras2 Kirsten rat sarcoma viral oncogene homolog | qRT-PCR//Western blot |
| hsa-miR-143 | *MAPK7* | 5598 | mitogen-activated protein kinase 7 | In situ hybridization//qRT-PCR//Western blot |
| hsa-miR-143 | *MYO6* | 4646 | myosin VI | qRT-PCR//Luciferase reporter assay//Western blot |
| hsa-miR-143 | *FHIT* | 2272 | fragile histidine triad | Luciferase reporter assay |
| hsa-miR-143 | *HK2* | 3099 | hexokinase 2 | Luciferase reporter assay |
| hsa-miR-143 | *MACC1* | 346389 | metastasis associated in colon cancer 1 | Luciferase reporter assay//qRT-PCR//Western blot |
| hsa-miR-143 | *MAPK7* | 5598 | mitogen-activated protein kinase 7 | Luciferase reporter assay//qRT-PCR//Western blot |
| hsa-miR-143 | *SERPINE1* | 5054 | serpin peptidase inhibitor, clade E (nexin, plasminogen activator inhibitor type 1), member 1 | Immunoblot//Luciferase reporter assay//qRT-PCR//Western blot |
| hsa-miR-145 | *BNIP3* | 664 | BCL2/adenovirus E1B 19kDa interacting protein 3 | Luciferase reporter assay |
| hsa-miR-145 | *C11orf9* | 745 | myelin regulatory factor | Luciferase reporter assay |
| hsa-miR-145 | *CBFB* | 865 | core-binding factor, beta subunit | qRT-PCR//Luciferase reporter assay//Microarray |
| hsa-miR-145 | *CDK4* | 1019 | cyclin-dependent kinase 4 | Luciferase reporter assay//qRT-PCR//Western blot |
| hsa-miR-145 | *CDKN1A* | 1026 | cyclin-dependent kinase inhibitor 1A (p21, Cip1) | qRT-PCR//Luciferase reporter assay//Western blot |
| hsa-miR-145 | *CLINT1* | 9685 | clathrin interactor 1 | qRT-PCR//Luciferase reporter assay//Microarray |
| hsa-miR-145 | *CPEB4* | 80315 | cytoplasmic polyadenylation element binding protein 4 | Luciferase reporter assay |
| hsa-miR-145 | *DFFA* | 1676 | DNA fragmentation factor, 45kDa, alpha polypeptide | Luciferase reporter assay//qRT-PCR//Western blot |
| hsa-miR-145 | *EIF4E* | 1977 | eukaryotic translation initiation factor 4E | Luciferase reporter assay//qRT-PCR//Western blot |
| hsa-miR-145 | *FLI1* | 2313 | Friend leukemia virus integration 1 | qRT-PCR//Luciferase reporter assay//Western blot |
| hsa-miR-145 | *FSCN1* | 6624 | fascin homolog 1, actin-bundling protein (Strongylocentrotus purpuratus) | Luciferase reporter assay//qRT-PCR//Western blot |
| hsa-miR-145 | *FZD7* | 8324 | frizzled family receptor 7 | Luciferase reporter assay |
| hsa-miR-145 | *HOXA9* | 3205 | homeobox A9 | GFP reporter assay//qRT-PCR//Western blot |
| hsa-miR-145 | *IFNB1* | 3456 | interferon, beta 1, fibroblast | ELISA//Luciferase reporter assay//qRT-PCR |
| hsa-miR-145 | *IGF1R* | 3480 | insulin-like growth factor 1 receptor | qRT-PCR//Western blot |
| hsa-miR-145 | *IRS1* | 3667 | insulin receptor substrate 1 | Luciferase reporter assay//Northern blot//qRT-PCR//Western blot |
| hsa-miR-145 | *KLF4* | 9314 | Kruppel-like factor 4 (gut) | FACS//Flow//GFP reporter assay//In situ hybridization//Luciferase reporter assay//qRT-PCR |
| hsa-miR-145 | *KRT7* | 3855 | keratin 7 | Immunoblot//qRT-PCR |
| hsa-miR-145 | *MUC1* | 4582 | mucin 1, cell surface associated | Luciferase reporter assay//Western blot |
| hsa-miR-145 | *MYC* | 4609 | v-myc myelocytomatosis viral oncogene homolog (avian) | qRT-PCR//Luciferase reporter assay//Western blot |
| hsa-miR-145 | *MYO6* | 4646 | myosin VI | qRT-PCR//Luciferase reporter assay//Western blot |
| hsa-miR-145 | *PARP8* | 79668 | poly (ADP-ribose) polymerase family, member 8 | Luciferase reporter assay |
| hsa-miR-145 | *POU5F1* | 5460 | POU class 5 homeobox 1 | FACS//Flow//GFP reporter assay//In situ hybridization//Immunoflorescence//Luciferase reporter assay//qRT-PCR |
| hsa-miR-145 | *PPM1D* | 8493 | protein phosphatase, Mg2+/Mn2+ dependent, 1D | Immunoblot//Luciferase reporter assay |
| hsa-miR-145 | *PPP3CA* | 5530 | protein phosphatase 3, catalytic subunit, alpha isozyme | qRT-PCR//Luciferase reporter assay//Microarray |
| hsa-miR-145 | *ROBO2* | 6092 | roundabout, axon guidance receptor, homolog 2 (Drosophila) | In situ hybridization//Luciferase reporter assay//qRT-PCR//western blot |
| hsa-miR-145 | *SOX2* | 6657 | SRY (sex determining region Y)-box 2 | FACS//Flow//GFP reporter assay//In situ hybridization//Luciferase reporter assay//qRT-PCR |
| hsa-miR-145 | *SRGAP1* | 57522 | SLIT-ROBO Rho GTPase activating protein 1 | In situ hybridization//Luciferase reporter assay//qRT-PCR//western blot |
| hsa-miR-145 | *STAT1* | 6772 | signal transducer and activator of transcription 1, 91kDa | qRT-PCR//Luciferase reporter assay//Western blot//Microarray |
| hsa-miR-145 | *TIRAP* | 114609 | toll-interleukin 1 receptor (TIR) domain containing adaptor protein | Immunoprecipitaion//Western blot//Communoprecipitaion |
| hsa-miR-145 | *TMOD3* | 29766 | tropomodulin 3 (ubiquitous) | Luciferase reporter assay |
| hsa-miR-145 | *YES1* | 7525 | v-yes-1 Yamaguchi sarcoma viral oncogene homolog 1 | qRT-PCR//Luciferase reporter assay//Western blot//Microarray |
| hsa-miR-145 | *IRS2* | 8660 | insulin receptor substrate 2 | Luciferase reporter assay |
| hsa-miR-145 | *ITGB8* | 3696 | integrin, beta 8 | Immunofluorescence//Luciferase reporter assay//qRT-PCR |
| hsa-miR-145 | *SERPINE1* | 5054 | serpin peptidase inhibitor, clade E (nexin, plasminogen activator inhibitor type 1), member 1 | Immunoblot//Luciferase reporter assay//qRT-PCR//Western blot |
| hsa-miR-145 | *VEGFA* | 7422 | vascular endothelial growth factor A | Luciferase reporter assay//qRT-PCR//Western blot |
| hsa-miR-146b | *CDKN1A* | 1026 | cyclin-dependent kinase inhibitor 1A (p21, Cip1) | Luciferase reporter assay |
| hsa-miR-146b | *IRAK1* | 3654 | interleukin-1 receptor-associated kinase 1 | Luciferase reporter assay//Microarray//Northern blot//qRT-PCR |
| hsa-miR-146b | *KIT* | 3815 | v-kit Hardy-Zuckerman 4 feline sarcoma viral oncogene homolog | Northern blot//qRT-PCR//Western blot |
| hsa-miR-146b | *MMP16* | 4325 | matrix metallopeptidase 16 (membrane-inserted) | Luciferase reporter assay//qRT-PCR//Western blot |
| hsa-miR-146b | *NFKB1* | 4790 | nuclear factor of kappa light polypeptide gene enhancer in B-cells 1 | Luciferase reporter assay |
| hsa-miR-146b | *TRAF6* | 7189 | TNF receptor-associated factor 6, E3 ubiquitin protein ligase | Luciferase reporter assay//Microarray//Northern blot//qRT-PCR |
| hsa-miR-15b | *BCL2* | 596 | B-cell CLL/lymphoma 2 | Luciferase reporter assay//qRT-PCR//Western blot |
| hsa-miR-15b | *CCND1* | 595 | cyclin D1 | qRT-PCR//Western blot |
| hsa-miR-15b | *CCNE1* | 898 | cyclin E1 | Luciferase reporter assay//qRT-PCR//Western blot |
| hsa-miR-15b | *EIF4A1* | 1973 | eukaryotic translation initiation factor 4A1 | Luciferase reporter assay |
| hsa-miR-15b | *RECK* | 8434 | reversion-inducing-cysteine-rich protein with kazal motifs | Western blot//qRT-PCR//Luciferase reporter assay |
| hsa-miR-15b | *VEGFA* | 7422 | vascular endothelial growth factor A | ELISA//Luciferase reporter assay |
| hsa-miR-16 | *ABCF2* | 10061 | ATP-binding cassette, sub-family F (GCN20), member 2 | pSILAC |
| hsa-miR-16 | *ABHD10* | 55347 | abhydrolase domain containing 10 | pSILAC |
| hsa-miR-16 | *ACP2* | 53 | acid phosphatase 2, lysosomal | pSILAC |
| hsa-miR-16 | *ACTR1A* | 10121 | ARP1 actin-related protein 1 homolog A, centractin alpha (yeast) | proteomics analysis |
| hsa-miR-16 | *ACVR2A* | 92 | activin A receptor, type IIA | Luciferase reporter assay//Western blot |
| hsa-miR-16 | *ADSS* | 159 | adenylosuccinate synthase | pSILAC |
| hsa-miR-16 | *ALG3* | 10195 | ALG3, alpha-1,3- mannosyltransferase | pSILAC |
| hsa-miR-16 | *ANAPC16* | 119504 | anaphase promoting complex subunit 16 | Microarray |
| hsa-miR-16 | *ARHGDIA* | 396 | Rho GDP dissociation inhibitor (GDI) alpha | pSILAC |
| hsa-miR-16 | *ARL2* | 402 | ADP-ribosylation factor-like 2 | Luciferase reporter assay//qRT-PCR//pSILAC//Western blot |
| hsa-miR-16 | *ASXL2* | 55252 | additional sex combs like 2 (Drosophila) | Microarray |
| hsa-miR-16 | *ATG9A* | 79065 | autophagy related 9A | pSILAC |
| hsa-miR-16 | *AURKB* | 9212 | aurora kinase B | Microarray//qRT-PCR |
| hsa-miR-16 | *BCL2* | 596 | B-cell CLL/lymphoma 2 | Luciferase reporter assay |
| hsa-miR-16 | *BMI1* | 648 | BMI1 polycomb ring finger oncogene | Luciferase reporter assay//Western blot |
| hsa-miR-16 | *BRCA1* | 672 | breast cancer 1, early onset | Luciferase reporter assay |
| hsa-miR-16 | *C14orf109* | 26175 | transmembrane protein 251 | Microarray |
| hsa-miR-16 | *C17orf80* | 55028 | chromosome 17 open reading frame 80 | Microarray |
| hsa-miR-16 | *C2orf43* | 60526 | chromosome 2 open reading frame 43 | Microarray |
| hsa-miR-16 | *C2orf74* | 339804 | chromosome 2 open reading frame 74 | Microarray |
| hsa-miR-16 | *C4orf27* | 54969 | chromosome 4 open reading frame 27 | Microarray |
| hsa-miR-16 | *C9orf114* | 51490 | chromosome 9 open reading frame 114 | pSILAC |
| hsa-miR-16 | *C9orf167* | 54863 | torsin family 4, member A | pSILAC |
| hsa-miR-16 | *C9orf89* | 84270 | chromosome 9 open reading frame 89 | pSILAC |
| hsa-miR-16 | *CA12* | 771 | carbonic anhydrase XII | pSILAC |
| hsa-miR-16 | *CACNA2D1* | 781 | calcium channel, voltage-dependent, alpha 2/delta subunit 1 | pSILAC |
| hsa-miR-16 | *CADM1* | 23705 | cell adhesion molecule 1 | Reporter assay//qRT-PCR |
| hsa-miR-16 | *CAPRIN1* | 4076 | cell cycle associated protein 1 | Luciferase reporter assay//qRT-PCR//Western blot |
| hsa-miR-16 | *CARD8* | 22900 | caspase recruitment domain family, member 8 | Microarray |
| hsa-miR-16 | *CCDC109A* | 90550 | mitochondrial calcium uniporter | pSILAC |
| hsa-miR-16 | *CCDC111* | 201973 | coiled-coil domain containing 111 | Microarray |
| hsa-miR-16 | *CCDC76* | 54482 | tRNA methyltransferase 13 homolog (S. cerevisiae) | Microarray |
| hsa-miR-16 | *CCND1* | 595 | cyclin D1 | Western blot |
| hsa-miR-16 | *CCND3* | 896 | cyclin D3 | qRT-PCR//flow//Luciferase reporter assay//Western blot |
| hsa-miR-16 | *CCNE1* | 898 | cyclin E1 | Luciferase reporter assay//qRT-PCR//Western blot |
| hsa-miR-16 | *CCNT2* | 905 | cyclin T2 | Luciferase reporter assay |
| hsa-miR-16 | *CDC14B* | 8555 | cell division cycle 14B | Microarray |
| hsa-miR-16 | *CDK5RAP1* | 51654 | CDK5 regulatory subunit associated protein 1 | pSILAC |
| hsa-miR-16 | *CDK6* | 1021 | cyclin-dependent kinase 6 | qRT-PCR//flow//Luciferase reporter assay//Western blot |
| hsa-miR-16 | *CENPJ* | 55835 | centromere protein J | Microarray |
| hsa-miR-16 | *CEP63* | 80254 | centrosomal protein 63kDa | Microarray |
| hsa-miR-16 | *CFL2* | 1073 | cofilin 2 (muscle) | proteomics analysis |
| hsa-miR-16 | *CHORDC1* | 26973 | cysteine and histidine-rich domain (CHORD) containing 1 | pSILAC |
| hsa-miR-16 | *CHUK* | 1147 | conserved helix-loop-helix ubiquitous kinase | Luciferase reporter assay//qRT-PCR//Western blot |
| hsa-miR-16 | *CREBL2* | 1389 | cAMP responsive element binding protein-like 2 | Microarray |
| hsa-miR-16 | *CRHBP* | 1393 | corticotropin releasing hormone binding protein | proteomics analysis |
| hsa-miR-16 | *CSHL1* | 1444 | chorionic somatomammotropin hormone-like 1 | proteomics analysis |
| hsa-miR-16 | *DNAJB4* | 11080 | DnaJ (Hsp40) homolog, subfamily B, member 4 | pSILAC |
| hsa-miR-16 | *ECHDC1* | 55862 | enoyl CoA hydratase domain containing 1 | Microarray |
| hsa-miR-16 | *EGFR* | 1956 | epidermal growth factor receptor | pSILAC |
| hsa-miR-16 | *EIF4E* | 1977 | eukaryotic translation initiation factor 4E | pSILAC |
| hsa-miR-16 | *EPT1* | 85465 | ethanolaminephosphotransferase 1 (CDP-ethanolamine-specific) | pSILAC |
| hsa-miR-16 | *F2* | 2147 | coagulation factor II (thrombin) | pSILAC |
| hsa-miR-16 | *FAM122C* | 159091 | family with sequence similarity 122, member C | Microarray |
| hsa-miR-16 | *FAM69A* | 388650 | family with sequence similarity 69, member A | Microarray |
| hsa-miR-16 | *FGF2* | 2247 | fibroblast growth factor 2 (basic) | pSILAC |
| hsa-miR-16 | *FNDC3B* | 64778 | fibronectin type III domain containing 3B | pSILAC |
| hsa-miR-16 | *GALNT7* | 51809 | UDP-N-acetyl-alpha-D-galactosamine:polypeptide N-acetylgalactosaminyltransferase 7 (GalNAc-T7) | pSILAC |
| hsa-miR-16 | *GFM1* | 85476 | G elongation factor, mitochondrial 1 | pSILAC |
| hsa-miR-16 | *GFPT1* | 2673 | glutamine--fructose-6-phosphate transaminase 1 | pSILAC |
| hsa-miR-16 | *GNL3L* | 54552 | guanine nucleotide binding protein-like 3 (nucleolar)-like | pSILAC |
| hsa-miR-16 | *GOLGA5* | 9950 | golgin A5 | Microarray |
| hsa-miR-16 | *GOLPH3L* | 55204 | golgi phosphoprotein 3-like | Microarray |
| hsa-miR-16 | *GPAM* | 57678 | glycerol-3-phosphate acyltransferase, mitochondrial | pSILAC |
| hsa-miR-16 | *GSTM4* | 2948 | glutathione S-transferase mu 4 | pSILAC |
| hsa-miR-16 | *GTF2H1* | 2965 | general transcription factor IIH, polypeptide 1, 62kDa | Microarray |
| hsa-miR-16 | *H3F3B* | 3021 | H3 histone, family 3B (H3.3B) | Microarray |
| hsa-miR-16 | *HACE1* | 57531 | HECT domain and ankyrin repeat containing E3 ubiquitin protein ligase 1 | Microarray |
| hsa-miR-16 | *HARS* | 3035 | histidyl-tRNA synthetase | pSILAC |
| hsa-miR-16 | *HARS2* | 23438 | histidyl-tRNA synthetase 2, mitochondrial | pSILAC |
| hsa-miR-16 | *HBXIP* | 10542 | late endosomal/lysosomal adaptor, MAPK and MTOR activator 5 | pSILAC |
| hsa-miR-16 | *HDHD2* | 84064 | haloacid dehalogenase-like hydrolase domain containing 2 | Microarray |
| hsa-miR-16 | *HERC6* | 55008 | HECT and RLD domain containing E3 ubiquitin protein ligase family member 6 | Microarray |
| hsa-miR-16 | *HMGA1* | 3159 | high mobility group AT-hook 1 | Luciferase reporter assay//qRT-PCR//Western blot |
| hsa-miR-16 | *HMOX1* | 3162 | heme oxygenase (decycling) 1 | pSILAC |
| hsa-miR-16 | *HRSP12* | 10247 | heat-responsive protein 12 | Microarray |
| hsa-miR-16 | *HSDL2* | 84263 | hydroxysteroid dehydrogenase like 2 | Microarray |
| hsa-miR-16 | *HSP90B1* | 7184 | heat shock protein 90kDa beta (Grp94), member 1 | Microarray |
| hsa-miR-16 | *HSPA1A* | 3303 | heat shock 70kDa protein 1A | Microarray |
| hsa-miR-16 | *IFRD1* | 3475 | interferon-related developmental regulator 1 | pSILAC |
| hsa-miR-16 | *IFRD2* | 7866 | interferon-related developmental regulator 2 | pSILAC |
| hsa-miR-16 | *IGF2R* | 3482 | insulin-like growth factor 2 receptor | pSILAC |
| hsa-miR-16 | *IPO4* | 79711 | importin 4 | pSILAC |
| hsa-miR-16 | *ITGA2* | 3673 | integrin, alpha 2 (CD49B, alpha 2 subunit of VLA-2 receptor) | pSILAC |
| hsa-miR-16 | *JUN* | 3725 | jun proto-oncogene | Microarray |
| hsa-miR-16 | *KCNN4* | 3783 | potassium intermediate/small conductance calcium-activated channel, subfamily N, member 4 | pSILAC |
| hsa-miR-16 | *KPNA3* | 3839 | karyopherin alpha 3 (importin alpha 4) | pSILAC |
| hsa-miR-16 | *LAMC1* | 3915 | laminin, gamma 1 (formerly LAMB2) | pSILAC |
| hsa-miR-16 | *LAMTOR2* | 28956 | late endosomal/lysosomal adaptor, MAPK and MTOR activator 2 | pSILAC |
| hsa-miR-16 | *LAMTOR3* | 8649 | late endosomal/lysosomal adaptor, MAPK and MTOR activator 3 | pSILAC |
| hsa-miR-16 | *LUZP1* | 7798 | leucine zipper protein 1 | pSILAC |
| hsa-miR-16 | *LYPLA2* | 11313 | lysophospholipase II | pSILAC |
| hsa-miR-16 | *MCL1* | 4170 | myeloid cell leukemia sequence 1 (BCL2-related) | Microarray |
| hsa-miR-16 | *MLLT11* | 10962 | myeloid/lymphoid or mixed-lineage leukemia (trithorax homolog, Drosophila); translocated to, 11 | pSILAC |
| hsa-miR-16 | *MMS19* | 64210 | MMS19 nucleotide excision repair homolog (S. cerevisiae) | pSILAC |
| hsa-miR-16 | *MRPL20* | 55052 | mitochondrial ribosomal protein L20 | pSILAC |
| hsa-miR-16 | *MSH2* | 4436 | mutS homolog 2, colon cancer, nonpolyposis type 1 (E. coli) | Microarray |
| hsa-miR-16 | *MYB* | 4602 | v-myb myeloblastosis viral oncogene homolog (avian) | Luciferase reporter assay//Microarray//qRT-PCR |
| hsa-miR-16 | *NAA15* | 80155 | N(alpha)-acetyltransferase 15, NatA auxiliary subunit | pSILAC |
| hsa-miR-16 | *NAA25* | 80018 | N(alpha)-acetyltransferase 25, NatB auxiliary subunit | pSILAC |
| hsa-miR-16 | *NAPG* | 8774 | N-ethylmaleimide-sensitive factor attachment protein, gamma | pSILAC |
| hsa-miR-16 | *NIPAL2* | 79815 | NIPA-like domain containing 2 | Microarray |
| hsa-miR-16 | *NOB1* | 28987 | NIN1/RPN12 binding protein 1 homolog (S. cerevisiae) | pSILAC |
| hsa-miR-16 | *NOTCH2* | 4853 | notch 2 | pSILAC |
| hsa-miR-16 | *NPR3* | 4883 | natriuretic peptide receptor C/guanylate cyclase C (atrionatriuretic peptide receptor C) | pSILAC |
| hsa-miR-16 | *NT5DC1* | 221294 | 5'-nucleotidase domain containing 1 | Microarray |
| hsa-miR-16 | *OMA1* | 115209 | OMA1 zinc metallopeptidase | Microarray |
| hsa-miR-16 | *OSGEPL1* | 64172 | O-sialoglycoprotein endopeptidase-like 1 | Microarray |
| hsa-miR-16 | *PAFAH1B2* | 5049 | platelet-activating factor acetylhydrolase 1b, catalytic subunit 2 (30kDa) | pSILAC |
| hsa-miR-16 | *PANX1* | 24145 | pannexin 1 | pSILAC |
| hsa-miR-16 | *PDCD4* | 27250 | programmed cell death 4 (neoplastic transformation inhibitor) | qRT-PCR |
| hsa-miR-16 | *PDCD6IP* | 10015 | programmed cell death 6 interacting protein | Microarray |
| hsa-miR-16 | *PHKB* | 5257 | phosphorylase kinase, beta | Microarray |
| hsa-miR-16 | *PHLDB2* | 90102 | pleckstrin homology-like domain, family B, member 2 | pSILAC |
| hsa-miR-16 | *PISD* | 23761 | phosphatidylserine decarboxylase | pSILAC |
| hsa-miR-16 | *PLK1* | 5347 | polo-like kinase 1 | pSILAC |
| hsa-miR-16 | *PMS1* | 5378 | PMS1 postmeiotic segregation increased 1 (S. cerevisiae) | Microarray |
| hsa-miR-16 | *PNN* | 5411 | pinin, desmosome associated protein | Microarray |
| hsa-miR-16 | *PNPLA6* | 10908 | patatin-like phospholipase domain containing 6 | pSILAC |
| hsa-miR-16 | *PPIF* | 10105 | peptidylprolyl isomerase F | pSILAC |
| hsa-miR-16 | *PPM1D* | 8493 | protein phosphatase, Mg2+/Mn2+ dependent, 1D | Northern blot//qRT-PCR//Western blot |
| hsa-miR-16 | *PPP2R5C* | 5527 | protein phosphatase 2, regulatory subunit B', gamma | pSILAC |
| hsa-miR-16 | *PRIM1* | 5557 | primase, DNA, polypeptide 1 (49kDa) | Microarray |
| hsa-miR-16 | *PSAT1* | 29968 | phosphoserine aminotransferase 1 | pSILAC |
| hsa-miR-16 | *PTCD3* | 55037 | pentatricopeptide repeat domain 3 | pSILAC |
| hsa-miR-16 | *PTGS2* | 5743 | prostaglandin-endoperoxide synthase 2 (prostaglandin G/H synthase and cyclooxygenase) | pSILAC |
| hsa-miR-16 | *PURA* | 5813 | purine-rich element binding protein A | pSILAC |
| hsa-miR-16 | *PWWP2A* | 114825 | PWWP domain containing 2A | Microarray |
| hsa-miR-16 | *RAB21* | 23011 | RAB21, member RAS oncogene family | qRT-PCR |
| hsa-miR-16 | *RAB30* | 27314 | RAB30, member RAS oncogene family | pSILAC |
| hsa-miR-16 | *RAB9B* | 51209 | RAB9B, member RAS oncogene family | proteomics analysis |
| hsa-miR-16 | *RAD51C* | 5889 | RAD51 homolog C (S. cerevisiae) | Microarray |
| hsa-miR-16 | *RARS* | 5917 | arginyl-tRNA synthetase | Northern blot//qRT-PCR |
| hsa-miR-16 | *RFT1* | 91869 | RFT1 homolog (S. cerevisiae) | pSILAC |
| hsa-miR-16 | *RHOT1* | 55288 | ras homolog family member T1 | Microarray |
| hsa-miR-16 | *RNASEL* | 6041 | ribonuclease L (2',5'-oligoisoadenylate synthetase-dependent) | Microarray |
| hsa-miR-16 | *RTN4* | 57142 | reticulon 4 | pSILAC |
| hsa-miR-16 | *SEC24A* | 10802 | SEC24 family, member A (S. cerevisiae) | pSILAC |
| hsa-miR-16 | *SERPINE2* | 5270 | serpin peptidase inhibitor, clade E (nexin, plasminogen activator inhibitor type 1), member 2 | pSILAC |
| hsa-miR-16 | *SHOC2* | 8036 | soc-2 suppressor of clear homolog (C. elegans) | pSILAC |
| hsa-miR-16 | *SKAP2* | 8935 | src kinase associated phosphoprotein 2 | Microarray//qRT-PCR |
| hsa-miR-16 | *SLC12A2* | 6558 | solute carrier family 12 (sodium/potassium/chloride transporters), member 2 | pSILAC |
| hsa-miR-16 | *SLC16A3* | 9123 | solute carrier family 16, member 3 (monocarboxylic acid transporter 4) | pSILAC |
| hsa-miR-16 | *SLC25A22* | 79751 | solute carrier family 25 (mitochondrial carrier: glutamate), member 22 | pSILAC |
| hsa-miR-16 | *SLC35A1* | 10559 | solute carrier family 35 (CMP-sialic acid transporter), member A1 | Microarray |
| hsa-miR-16 | *SLC35B3* | 51000 | solute carrier family 35, member B3 | Microarray |
| hsa-miR-16 | *SLC38A1* | 81539 | solute carrier family 38, member 1 | pSILAC |
| hsa-miR-16 | *SLC38A5* | 92745 | solute carrier family 38, member 5 | pSILAC |
| hsa-miR-16 | *SLC7A1* | 6541 | solute carrier family 7 (cationic amino acid transporter, y+ system), member 1 | pSILAC |
| hsa-miR-16 | *SPTLC1* | 10558 | serine palmitoyltransferase, long chain base subunit 1 | pSILAC |
| hsa-miR-16 | *SQSTM1* | 8878 | sequestosome 1 | pSILAC |
| hsa-miR-16 | *SRPR* | 6734 | signal recognition particle receptor (docking protein) | pSILAC |
| hsa-miR-16 | *SRPRB* | 58477 | signal recognition particle receptor, B subunit | pSILAC |
| hsa-miR-16 | *TIA1* | 7072 | TIA1 cytotoxic granule-associated RNA binding protein | Microarray |
| hsa-miR-16 | *TMEM109* | 79073 | transmembrane protein 109 | pSILAC |
| hsa-miR-16 | *TMEM43* | 79188 | transmembrane protein 43 | pSILAC |
| hsa-miR-16 | *TNFSF9* | 8744 | tumor necrosis factor (ligand) superfamily, member 9 | pSILAC |
| hsa-miR-16 | *TOMM34* | 10953 | translocase of outer mitochondrial membrane 34 | pSILAC |
| hsa-miR-16 | *TP53* | 7157 | tumor protein p53 | Immunoblot//Luciferase reporter assay |
| hsa-miR-16 | *TPI1* | 7167 | triosephosphate isomerase 1 | proteomics analysis |
| hsa-miR-16 | *TPM3* | 7170 | tropomyosin 3 | pSILAC |
| hsa-miR-16 | *TPPP3* | 51673 | tubulin polymerization-promoting protein family member 3 | Luciferase reporter assay |
| hsa-miR-16 | *TXN2* | 25828 | thioredoxin 2 | pSILAC |
| hsa-miR-16 | *UBE2S* | 27338 | ubiquitin-conjugating enzyme E2S | pSILAC |
| hsa-miR-16 | *UBE2V1* | 7335 | ubiquitin-conjugating enzyme E2 variant 1 | pSILAC |
| hsa-miR-16 | *UBE4A* | 9354 | ubiquitination factor E4A | pSILAC |
| hsa-miR-16 | *UGDH* | 7358 | UDP-glucose 6-dehydrogenase | Microarray |
| hsa-miR-16 | *UGP2* | 7360 | UDP-glucose pyrophosphorylase 2 | Microarray |
| hsa-miR-16 | *UTP15* | 84135 | UTP15, U3 small nucleolar ribonucleoprotein, homolog (S. cerevisiae) | pSILAC |
| hsa-miR-16 | *VEGFA* | 7422 | vascular endothelial growth factor A | Luciferase reporter assay |
| hsa-miR-16 | *VPS45* | 11311 | vacuolar protein sorting 45 homolog (S. cerevisiae) | Microarray |
| hsa-miR-16 | *VTI1B* | 10490 | vesicle transport through interaction with t-SNAREs 1B | pSILAC |
| hsa-miR-16 | *WIPF1* | 7456 | WAS/WASL interacting protein family, member 1 | Microarray |
| hsa-miR-16 | *WNT3A* | 89780 | wingless-type MMTV integration site family, member 3A | Luciferase reporter assay |
| hsa-miR-16 | *WT1* | 7490 | Wilms tumor 1 | qRT-PCR//proteomics analysis |
| hsa-miR-16 | *YIF1B* | 90522 | Yip1 interacting factor homolog B (S. cerevisiae) | pSILAC |
| hsa-miR-16 | *ZNF384* | 171017 | zinc finger protein 384 | pSILAC |
| hsa-miR-16 | *ZNF559* | 84527 | zinc finger protein 559 | Microarray |
| hsa-miR-16 | *ZNF622* | 90441 | zinc finger protein 662 | pSILAC |
| hsa-miR-16 | *NFKB1* | 4790 | nuclear factor of kappa light polypeptide gene enhancer in B-cells 1 | ChIP-seq |
| hsa-miR-16 | *ZYX* | 7791 | zyxin | Luciferase reporter assay//qRT-PCR//Western blot |
| hsa-miR-181b | *BCL2* | 596 | B-cell CLL/lymphoma 2 | Luciferase reporter assay//qRT-PCR//Western blot |
| hsa-miR-181b | *CDX2* | 1045 | caudal type homeobox 2 | Luciferase reporter assay |
| hsa-miR-181b | *CYLD* | 1540 | cylindromatosis (turban tumor syndrome) | Luciferase reporter assay//qRT-PCR |
| hsa-miR-181b | *GATA6* | 2627 | GATA binding protein 6 | Luciferase reporter assay |
| hsa-miR-181b | *GRIA2* | 2891 | glutamate receptor, ionotropic, AMPA 2 | Luciferase assay/RT-PCR |
| hsa-miR-181b | *KAT2B* | 8850 | K(lysine) acetyltransferase 2B | Western blot//Luciferase reporter assay |
| hsa-miR-181b | *MAP3K10* | 4294 | mitogen-activated protein kinase kinase kinase 10 | qRT-PCR//Western blot |
| hsa-miR-181b | *NLK* | 51701 | nemo-like kinase | Luciferase reporter assay |
| hsa-miR-181b | *PLAG1* | 5324 | pleiomorphic adenoma gene 1 | Western blot//Luciferase reporter assay//Microarray |
| hsa-miR-181b | *TCL1A* | 8115 | T-cell leukemia/lymphoma 1A | Luciferase reporter assay//Microarray//qRT-PCR//Western blot |
| hsa-miR-181b | *TIMP3* | 7078 | TIMP metallopeptidase inhibitor 3 | Luciferase reporter assay//qRT-PCR//Western blot |
| hsa-miR-181b | *VSNL1* | 7447 | visinin-like 1 | Luciferase assay/RT-PCR |
| hsa-miR-181b | *CBX7* | 23492 | chromobox homolog 7 | Luciferase reporter assay |
| hsa-miR-181b | *RNF2* | 6045 | ring finger protein 2 | Luciferase reporter assay//Microarray//qRT-PCR//Western blot |
| hsa-miR-181b | *SIRT1* | 23411 | sirtuin 1 | Luciferase reporter assay |
| hsa-miR-181b | *TMED7* | 51014 | transmembrane emp24 protein transport domain containing 7 | Luciferase reporter assay//qRT-PCR//Western blot |
| hsa-miR-181c | *BCL2* | 596 | B-cell CLL/lymphoma 2 | Luciferase reporter assay//qRT-PCR//Western blot |
| hsa-miR-181c | *CDX2* | 1045 | caudal type homeobox 2 | Luciferase reporter assay |
| hsa-miR-181c | *GATA6* | 2627 | GATA binding protein 6 | Luciferase reporter assay |
| hsa-miR-181c | *KRAS* | 3845 | v-Ki-ras2 Kirsten rat sarcoma viral oncogene homolog | Luciferase reporter assay//qRT-PCR//Western blot |
| hsa-miR-181c | *NLK* | 51701 | nemo-like kinase | Luciferase reporter assay |
| hsa-miR-181c | *NOTCH2* | 4853 | notch 2 | Luciferase reporter assay//qRT-PCR//Western blot |
| hsa-miR-181c | *NOTCH4* | 4855 | notch 4 | Luciferase reporter assay//qRT-PCR//Western blot |
| hsa-miR-182 | *ADCY6* | 112 | adenylate cyclase 6 | Luciferase reporter assay |
| hsa-miR-182 | *CDKN1A* | 1026 | cyclin-dependent kinase inhibitor 1A (p21, Cip1) | qRT-PCR//Luciferase reporter assay//Western blot |
| hsa-miR-182 | *CLOCK* | 9575 | clock circadian regulator | Luciferase reporter assay//qRT-PCR |
| hsa-miR-182 | *EP300* | 2033 | E1A binding protein p300 | Microarray |
| hsa-miR-182 | *FOXO1* | 2308 | forkhead box O1 | qRT-PCR//Luciferase reporter assay//Western blot |
| hsa-miR-182 | *FOXO3* | 2309 | forkhead box O3 | Luciferase reporter assay//Western blot |
| hsa-miR-182 | *MITF* | 4286 | microphthalmia-associated transcription factor | Luciferase reporter assay |
| hsa-miR-182 | *RARG* | 5916 | retinoic acid receptor, gamma | Luciferase reporter assay//Western blot |
| hsa-miR-182 | *TSC22D3* | 1831 | TSC22 domain family, member 3 | Luciferase reporter assay//qRT-PCR |
| hsa-miR-182 | *CREB1* | 1385 | cAMP responsive element binding protein 1 | GFP reporter assay |
| hsa-miR-182 | *MTSS1* | 9788 | metastasis suppressor 1 | Luciferase reporter assay |
| hsa-miR-183 | *AKAP12* | 9590 | A kinase (PRKA) anchor protein 12 | Immunohistochemistry//Luciferase reporter assay//Microarray//qRT-PCR//Western blot |
| hsa-miR-183 | *BTRC* | 8945 | beta-transducin repeat containing E3 ubiquitin protein ligase | 5'RACE//qRT-PCR//Western blot |
| hsa-miR-183 | *DAP* | 1611 | death-associated protein | qRT-PCR |
| hsa-miR-183 | *EZR* | 7430 | ezrin | Luciferase reporter assay |
| hsa-miR-183 | *FOXO1* | 2308 | forkhead box O1 | Immunohistochemistry//Northern blot//qRT-PCR//Western blot |
| hsa-miR-183 | *ITGB1* | 3688 | integrin, beta 1 (fibronectin receptor, beta polypeptide, antigen CD29 includes MDF2, MSK12) | Immunoblot//Immunocytochemistry//Luciferase reporter assay//qRT-PCR |
| hsa-miR-183 | *KIF2A* | 3796 | kinesin heavy chain member 2A | Immunoblot//Immunocytochemistry//Luciferase reporter assay//qRT-PCR |
| hsa-miR-183 | *PDCD4* | 27250 | programmed cell death 4 (neoplastic transformation inhibitor) | Luciferase reporter assay//qRT-PCR//Western blot |
| hsa-miR-183 | *PDCD6* | 10016 | programmed cell death 6 | qRT-PCR |
| hsa-miR-183 | *SRSF2* | 6427 | serine/arginine-rich splicing factor 2 | Immunoblot//Microarray//qRT-PCR |
| hsa-miR-183 | *EGR1* | 1958 | early growth response 1 | Luciferase reporter assay |
| hsa-miR-193b | *CCND1* | 595 | cyclin D1 | qRT-PCR//Luciferase reporter assay//Western blot//Microarray |
| hsa-miR-193b | *ESR1* | 2099 | estrogen receptor 1 | Luciferase reporter assay//Microarray |
| hsa-miR-193b | *ETS1* | 2113 | v-ets erythroblastosis virus E26 oncogene homolog 1 (avian) | Luciferase reporter assay//qRT-PCR//Western blot |
| hsa-miR-193b | *MCL1* | 4170 | myeloid cell leukemia sequence 1 (BCL2-related) | Luciferase reporter assay//Western blot |
| hsa-miR-193b | *PLAU* | 5328 | plasminogen activator, urokinase | Western blot//Luciferase reporter assay |
| hsa-miR-193b | *PRAP1* | 118471 | proline-rich acidic protein 1 | Luciferase reporter assay |
| hsa-miR-19b | *ARID4B* | 51742 | AT rich interactive domain 4B (RBP1-like) | Luciferase reporter assay |
| hsa-miR-19b | *ATXN1* | 6310 | ataxin 1 | Luciferase reporter assay//qRT-PCR//Western blot |
| hsa-miR-19b | *BACE1* | 23621 | beta-site APP-cleaving enzyme 1 | Luciferase reporter assay |
| hsa-miR-19b | *BCL2L11* | 10018 | BCL2-like 11 (apoptosis facilitator) | Luciferase reporter assay |
| hsa-miR-19b | *BMPR2* | 659 | bone morphogenetic protein receptor, type II (serine/threonine kinase) | Luciferase reporter assay//qRT-PCR//Western blot |
| hsa-miR-19b | *ESR1* | 2099 | estrogen receptor 1 | Western blot |
| hsa-miR-19b | *HIPK3* | 10114 | homeodomain interacting protein kinase 3 | Luciferase reporter assay |
| hsa-miR-19b | *KAT2B* | 8850 | K(lysine) acetyltransferase 2B | Western blot//Luciferase reporter assay |
| hsa-miR-19b | *MYLIP* | 29116 | myosin regulatory light chain interacting protein | Luciferase reporter assay |
| hsa-miR-19b | *NCOA3* | 8202 | nuclear receptor coactivator 3 | Western blot |
| hsa-miR-19b | *PTEN* | 5728 | phosphatase and tensin homolog | Luciferase reporter assay//qRT-PCR//Western blot |
| hsa-miR-19b | *SOCS1* | 8651 | suppressor of cytokine signaling 1 | Luciferase reporter assay//Western blot |
| hsa-miR-19b | *TGFBR2* | 7048 | transforming growth factor, beta receptor II (70/80kDa) | Microarray//qRT-PCR |
| hsa-miR-19b | *CUL5* | 8065 | cullin 5 | qRT-PCR//Western blot |
| hsa-miR-203 | *ABCE1* | 6059 | ATP-binding cassette, sub-family E (OABP), member 1 | Luciferase reporter assay//qRT-PCR//Western blot |
| hsa-miR-203 | *ABL1* | 25 | c-abl oncogene 1, non-receptor tyrosine kinase | Luciferase reporter assay |
| hsa-miR-203 | *BCL2L2* | 599 | BCL2-like 2 | Luciferase reporter assay//qRT-PCR//Western blot |
| hsa-miR-203 | *CDK6* | 1021 | cyclin-dependent kinase 6 | Luciferase reporter assay//qRT-PCR//Western blot |
| hsa-miR-203 | *EDNRA* | 1909 | endothelin receptor type A | Immunofluorescence//In situ hybridization//Luciferase reporter assay//Northern blot//qRT-PCR//Western blot |
| hsa-miR-203 | *EYA4* | 2070 | eyes absent homolog 4 (Drosophila) | Immunofluorescence//In situ hybridization//Luciferase reporter assay//Northern blot//qRT-PCR//Western blot |
| hsa-miR-203 | *GDAP1* | 54332 | ganglioside induced differentiation associated protein 1 | Immunofluorescence//In situ hybridization//Luciferase reporter assay//Northern blot//qRT-PCR |
| hsa-miR-203 | *PPM1D* | 8493 | protein phosphatase, Mg2+/Mn2+ dependent, 1D | Immunoblot//Luciferase reporter assay |
| hsa-miR-203 | *SOCS3* | 9021 | suppressor of cytokine signaling 3 | Immunohistochemistry//In situ hybridization//Luciferase reporter assay//Microarray//qRT-PCRWestern blot |
| hsa-miR-203 | *TP63* | 8626 | tumor protein p63 | Luciferase reporter assay |
| hsa-miR-203 | *AKT2* | 208 | v-akt murine thymoma viral oncogene homolog 2 | Northern blot//qRT-PCR//Western blot |
| hsa-miR-203 | *BIRC5* | 332 | baculoviral IAP repeat containing 5 | Luciferase reporter assay |
| hsa-miR-203 | *BMI1* | 648 | BMI1 polycomb ring finger oncogene | Immunoblot//In situ hybridization//Luciferase reporter assay//qRT-PCR//Western blot |
| hsa-miR-203 | *CREB1* | 1385 | cAMP responsive element binding protein 1 | Luciferase reporter assay//qRT-PCR |
| hsa-miR-203 | *DLX5* | 1749 | distal-less homeobox 5 | Immunoblot//In situ hybridization//Luciferase reporter assay//qRT-PCR//Western blot |
| hsa-miR-203 | *E2F1* | 1869 | E2F transcription factor 1 | Immunoblot//In situ hybridization//Luciferase reporter assay//qRT-PCR//Western blot |
| hsa-miR-203 | *E2F3* | 1871 | E2F transcription factor 3 | Luciferase reporter assay |
| hsa-miR-203 | *IL6* | 3569 | interleukin 6 (interferon, beta 2) | ELISA//qRT-PCR |
| hsa-miR-203 | *MMP1* | 4312 | matrix metallopeptidase 1 (interstitial collagenase) | ELISA//qRT-PCR |
| hsa-miR-203 | *MMP10* | 4319 | matrix metallopeptidase 10 (stromelysin 2) | Immunoblot//In situ hybridization//Luciferase reporter assay//qRT-PCR//Western blot |
| hsa-miR-203 | *RUNX2* | 860 | runt-related transcription factor 2 | Immunoblot//In situ hybridization//Luciferase reporter assay//qRT-PCR//Western blot |
| hsa-miR-203 | *SMAD4* | 4089 | SMAD family member 4 | Immunoblot//In situ hybridization//Luciferase reporter assay//qRT-PCR//Western blot |
| hsa-miR-203 | *ZEB2* | 9839 | zinc finger E-box binding homeobox 2 | Immunoblot//In situ hybridization//Luciferase reporter assay//qRT-PCR//Western blot |
| hsa-miR-203 | *ZNF148* | 7707 | zinc finger protein 148 | Luciferase reporter assay |
| hsa-miR-210 | *ABCB9* | 23457 | ATP-binding cassette, sub-family B (MDR/TAP), member 9 | immunoprecipitaion//Microarray//qRT-PCR |
| hsa-miR-210 | *ACVR1B* | 91 | activin A receptor, type IB | immunoprecipitaion//qRT-PCR |
| hsa-miR-210 | *AIFM3* | 150209 | apoptosis-inducing factor, mitochondrion-associated, 3 | Luciferase reporter assay |
| hsa-miR-210 | *APC* | 324 | adenomatosis polyposis coli | immunoprecipitaion//Microarray//qRT-PCR |
| hsa-miR-210 | *ATP11C* | 286410 | ATPase, class VI, type 11C | immunoprecipitaion//Microarray//qRT-PCR |
| hsa-miR-210 | *BDNF* | 627 | brain-derived neurotrophic factor | 2DGE//immunoprecipitaion//Luciferase reporter assay//Mass spectrometry//Microarray//qRT-PCR//Western blot |
| hsa-miR-210 | *CASP8AP2* | 9994 | caspase 8 associated protein 2 | Luciferase reporter assay |
| hsa-miR-210 | *CBX1* | 10951 | chromobox homolog 1 | immunoprecipitaion//Microarray//qRT-PCR |
| hsa-miR-210 | *CDK10* | 8558 | cyclin-dependent kinase 10 | immunoprecipitaion//Microarray//qRT-PCR |
| hsa-miR-210 | *CHD9* | 80205 | chromodomain helicase DNA binding protein 9 | immunoprecipitaion//Microarray//qRT-PCR |
| hsa-miR-210 | *CLASP2* | 23122 | cytoplasmic linker associated protein 2 | immunoprecipitaion//Microarray//qRT-PCR |
| hsa-miR-210 | *CPEB2* | 132864 | cytoplasmic polyadenylation element binding protein 2 | immunoprecipitaion//Luciferase reporter assay//Microarray//qRT-PCR |
| hsa-miR-210 | *DDAH1* | 23576 | dimethylarginine dimethylaminohydrolase 1 | immunoprecipitaion//Luciferase reporter assay//Microarray//qRT-PCR |
| hsa-miR-210 | *E2F3* | 1871 | E2F transcription factor 3 | immunoprecipitaion//qRT-PCR |
| hsa-miR-210 | *EFNA3* | 1944 | ephrin-A3 | Luciferase reporter assay//Western blot |
| hsa-miR-210 | *ELK3* | 2004 | ELK3, ETS-domain protein (SRF accessory protein 2) | immunoprecipitaion//Microarray//qRT-PCR |
| hsa-miR-210 | *FAM116A* | 201627 | DENN/MADD domain containing 6A | immunoprecipitaion//Microarray//qRT-PCR |
| hsa-miR-210 | *FGFRL1* | 53834 | fibroblast growth factor receptor-like 1 | Luciferase reporter assay//Microarray//qRT-PCR//Western blot |
| hsa-miR-210 | *GPD1L* | 23171 | glycerol-3-phosphate dehydrogenase 1-like | immunoprecipitaion//Luciferase reporter assay//Microarray//qRT-PCR |
| hsa-miR-210 | *HECTD1* | 25831 | HECT domain containing E3 ubiquitin protein ligase 1 | immunoprecipitaion//Microarray//qRT-PCR |
| hsa-miR-210 | *HOXA1* | 3198 | homeobox A1 | Luciferase reporter assay |
| hsa-miR-210 | *HOXA3* | 3200 | homeobox A3 | immunoprecipitaion//Microarray//qRT-PCR |
| hsa-miR-210 | *HOXA9* | 3205 | homeobox A9 | Luciferase reporter assay |
| hsa-miR-210 | *ISCU* | 23479 | iron-sulfur cluster scaffold homolog (E. coli) | immunoprecipitaion//Microarray//qRT-PCR |
| hsa-miR-210 | *KIAA1161* | 57462 | KIAA1161 | immunoprecipitaion//Microarray//qRT-PCR |
| hsa-miR-210 | *MDGA1* | 266727 | MAM domain containing glycosylphosphatidylinositol anchor 1 | immunoprecipitaion//Microarray//qRT-PCR |
| hsa-miR-210 | *MIB1* | 57534 | mindbomb E3 ubiquitin protein ligase 1 | immunoprecipitaion//Microarray//qRT-PCR |
| hsa-miR-210 | *MID1IP1* | 58526 | MID1 interacting protein 1 | immunoprecipitaion//Microarray//qRT-PCR |
| hsa-miR-210 | *MNT* | 4335 | MNT, MAX dimerization protein | Luciferase reporter assay//qRT-PCR//Western blot |
| hsa-miR-210 | *MRE11A* | 4361 | MRE11 meiotic recombination 11 homolog A (S. cerevisiae) | Western blot |
| hsa-miR-210 | *NCAM1* | 4684 | neural cell adhesion molecule 1 | immunoprecipitaion//Luciferase reporter assay//Microarray//qRT-PCR |
| hsa-miR-210 | *NIPBL* | 25836 | Nipped-B homolog (Drosophila) | immunoprecipitaion//Microarray//qRT-PCR |
| hsa-miR-210 | *NPTX1* | 4884 | neuronal pentraxin I | Luciferase reporter assay |
| hsa-miR-210 | *P4HB* | 5034 | prolyl 4-hydroxylase, beta polypeptide | 2DGE//immunoprecipitaion//Mass spectrometry//Microarray//qRT-PCR//Western blot |
| hsa-miR-210 | *PIM1* | 5292 | pim-1 oncogene | Luciferase reporter assay |
| hsa-miR-210 | *PTAR1* | 375743 | protein prenyltransferase alpha subunit repeat containing 1 | immunoprecipitaion//Microarray//qRT-PCR |
| hsa-miR-210 | *PTPN1* | 5770 | protein tyrosine phosphatase, non-receptor type 1 | 2DGE//immunoprecipitaion//Luciferase reporter assay//Mass spectrometry//Microarray//qRT-PCR//Western blot |
| hsa-miR-210 | *RAD52* | 5893 | RAD52 homolog (S. cerevisiae) | Luciferase reporter assay//qRT-PCR//Western blot |
| hsa-miR-210 | *SEH1L* | 81929 | SEH1-like (S. cerevisiae) | immunoprecipitaion//Microarray//qRT-PCR |
| hsa-miR-210 | *SERTAD2* | 9792 | SERTA domain containing 2 | immunoprecipitaion//Microarray//qRT-PCR |
| hsa-miR-210 | *SMCHD1* | 23347 | structural maintenance of chromosomes flexible hinge domain containing 1 | immunoprecipitaion//Microarray//qRT-PCR |
| hsa-miR-210 | *TNPO1* | 3842 | transportin 1 | immunoprecipitaion//Microarray//qRT-PCR |
| hsa-miR-210 | *TP53I11* | 9537 | tumor protein p53 inducible protein 11 | Luciferase reporter assay |
| hsa-miR-210 | *UBQLN1* | 29979 | ubiquilin 1 | immunoprecipitaion//Microarray//qRT-PCR |
| hsa-miR-210 | *XIST* | 7503 | X inactive specific transcript (non-protein coding) | immunoprecipitaion//Luciferase reporter assay//Microarray//qRT-PCR |
| hsa-miR-210 | *XPA* | 7507 | xeroderma pigmentosum, complementation group A | Western blot |
| hsa-miR-212 | *BRCA1* | 672 | breast cancer 1, early onset | Microarray |
| hsa-miR-212 | *MECP2* | 4204 | methyl CpG binding protein 2 (Rett syndrome) | Immunohistochemistry//Luciferase reporter assay//Microarray//qRT-PCR//Western blot |
| hsa-miR-212 | *PEA15* | 8682 | phosphoprotein enriched in astrocytes 15 | qRT-PCR//Luciferase reporter assay//Western blot |
| hsa-miR-212 | *TJP1* | 7082 | tight junction protein 1 | Western blot |
| hsa-miR-212 | *WT1* | 7490 | Wilms tumor 1 | Microarray |
| hsa-miR-212 | *PTCH1* | 5727 | patched homolog 1 | Luciferase reporter assay//qRT-PCR//Western blot |
| hsa-miR-212 | *RB1* | 5925 | retinoblastoma 1 | Luciferase reporter assay//Western blot |
| hsa-miR-222 | *BBC3* | 27113 | BCL2 binding component 3 | Immunohistochemistry//In situ hybridization//Luciferase reporter assay//Northern blot//Western blot |
| hsa-miR-222 | *CDKN1B* | 1027 | cyclin-dependent kinase inhibitor 1B (p27, Kip1) | Luciferase reporter assay//Western blot |
| hsa-miR-222 | *CDKN1C* | 1028 | cyclin-dependent kinase inhibitor 1C (p57, Kip2) | Luciferase reporter assay//Western blot |
| hsa-miR-222 | *CORO1A* | 11151 | coronin, actin binding protein, 1A | Western blot |
| hsa-miR-222 | *ESR1* | 2099 | estrogen receptor 1 | Luciferase reporter assay//qRT-PCR//Western blot |
| hsa-miR-222 | *FOS* | 2353 | FBJ murine osteosarcoma viral oncogene homolog | qRT-PCR//Luciferase reporter assay//Western blot//Northern blot |
| hsa-miR-222 | *FOXO3* | 2309 | forkhead box O3 | qRT-PCR//ChIP//Luciferase reporter assay//Western blot//Northern blot |
| hsa-miR-222 | *ICAM1* | 3383 | intercellular adhesion molecule 1 | Luciferase reporter assay |
| hsa-miR-222 | *KIT* | 3815 | v-kit Hardy-Zuckerman 4 feline sarcoma viral oncogene homolog | qRT-PCR//Western blot |
| hsa-miR-222 | *MMP1* | 4312 | matrix metallopeptidase 1 (interstitial collagenase) | Flow//Luciferase reporter assay//Microarray//qRT-PCR//Western blot |
| hsa-miR-222 | *PPP2R2A* | 5520 | protein phosphatase 2, regulatory subunit B, alpha | Luciferase reporter assay//Western blot |
| hsa-miR-222 | *PTEN* | 5728 | phosphatase and tensin homolog | FACS//Flow//Luciferase reporter assay//Northern blot//Western blot |
| hsa-miR-222 | *SELE* | 6401 | phosphatase and tensin homolog | Luciferase reporter assay |
| hsa-miR-222 | *SOD2* | 6648 | superoxide dismutase 2, mitochondrial | Flow//Luciferase reporter assay//Microarray//qRT-PCR//Western blot |
| hsa-miR-222 | *SSSCA1* | 10534 | Sjogren syndrome/scleroderma autoantigen 1 | Western blot |
| hsa-miR-222 | *STAT5A* | 6776 | signal transducer and activator of transcription 5A | qRT-PCR//Luciferase reporter assay//Western blot |
| hsa-miR-222 | *TCEAL1* | 9338 | transcription elongation factor A (SII)-like 1 | Western blot |
| hsa-miR-222 | *TNFSF10* | 8743 | tumor necrosis factor (ligand) superfamily, member 10 | Western blot |
| hsa-miR-222 | *TP53* | 7157 | tumor protein p53 | Western blot |
| hsa-miR-222 | *DICER1* | 23405 | dicer 1, ribonuclease type III | Luciferase reporter assay |
| hsa-miR-222 | *DIRAS3* | 9077 | DIRAS family, GTP-binding RAS-like 3 | Immunohistochemistry//Luciferase reporter assay//qRT-PCR//Western blot |
| hsa-miR-222 | *ETS1* | 2113 | v-ets erythroblastosis virus E26 oncogene homolog 1 (avian) | Luciferase reporter assay//Northern blot//qRT-PCR//Western blot |
| hsa-miR-222 | *PTEN* | 5728 | phosphatase and tensin homolog | Flow//Immunohistochemistry//Luciferase reporter assay//qRT-PCR//Western blot |
| hsa-miR-222 | *TIMP3* | 7078 | TIMP metallopeptidase inhibitor 3 | Flow//Immunohistochemistry//Luciferase reporter assay//qRT-PCR//Western blot |
| hsa-miR-24 | *ACVR1B* | 91 | activin A receptor, type IB | Western blot//Luciferase reporter assay |
| hsa-miR-24 | *AURKB* | 9212 | aurora kinase B | Luciferase reporter assay//Microarray//qRT-PCR//Western blot |
| hsa-miR-24 | *BRCA1* | 672 | breast cancer 1, early onset | Luciferase reporter assay//Microarray//qRT-PCR//Western blot |
| hsa-miR-24 | *CCNA2* | 890 | cyclin A2 | Luciferase reporter assay//Microarray//qRT-PCR//Western blot |
| hsa-miR-24 | *CDK4* | 1019 | cyclin-dependent kinase 4 | Luciferase reporter assay//Microarray//qRT-PCR//Western blot |
| hsa-miR-24 | *CDKN1B* | 1027 | cyclin-dependent kinase inhibitor 1B (p27, Kip1) | Luciferase reporter assay |
| hsa-miR-24 | *CDKN2A* | 1029 | cyclin-dependent kinase inhibitor 2A | qRT-PCR//Western blot |
| hsa-miR-24 | *DHFR* | 1719 | dihydrofolate reductase | Western blot |
| hsa-miR-24 | *DND1* | 373863 | dead end homolog 1 (zebrafish) | Luciferase reporter assay//qRT-PCR//Western blot |
| hsa-miR-24 | *E2F2* | 1870 | E2F transcription factor 2 | Luciferase reporter assay//Microarray//qRT-PCR//Western blot |
| hsa-miR-24 | *FEN1* | 2237 | flap structure-specific endonuclease 1 | Luciferase reporter assay//Microarray//qRT-PCR//Western blot |
| hsa-miR-24 | *FURIN* | 5045 | furin (paired basic amino acid cleaving enzyme) | Luciferase reporter assay//Microarray//qRT-PCR//Western blot |
| hsa-miR-24 | *HNF4A* | 3172 | hepatocyte nuclear factor 4, alpha | ELISA//Flow//Immunohistochemistry//Immunoprecipitaion//In situ hybridization//Luciferase reporter assay//Microarray//qRT-PCR//Western blot |
| hsa-miR-24 | *KHSRP* | 8570 | KH-type splicing regulatory protein | qRT-PCR |
| hsa-miR-24 | *MAPK14* | 1432 | mitogen-activated protein kinase 14 | Luciferase reporter assay |
| hsa-miR-24 | *MLEC* | 9761 | malectin | Luciferase reporter assay |
| hsa-miR-24 | *MYC* | 4609 | v-myc myelocytomatosis viral oncogene homolog (avian) | Luciferase reporter assay//Microarray//qRT-PCR//Western blot |
| hsa-miR-24 | *NFAT5* | 10725 | nuclear factor of activated T-cells 5, tonicity-responsive | qRT-PCR |
| hsa-miR-24 | *POLD1* | 5424 | polymerase (DNA directed), delta 1, catalytic subunit | Luciferase reporter assay//Microarray//qRT-PCR//Western blot |
| hsa-miR-24 | *TGFB1* | 7040 | transforming growth factor, beta 1 | Luciferase reporter assay//Microarray//qRT-PCR//Western blot |
| hsa-miR-24 | *TRIB3* | 57761 | tribbles homolog 3 (Drosophila) | qRT-PCR//Western blot |
| hsa-miR-24 | *FAF1* | 11124 | Fas (TNFRSF6) associated factor 1 | Luciferase reporter assay//Western blot |
| hsa-miR-26b | *EP300* | 2033 | E1A binding protein p300 | Microarray |
| hsa-miR-26b | *EPHA2* | 1969 | EPH receptor A2 | Luciferase reporter assay//qRT-PCR//Western blot |
| hsa-miR-26b | *PTGS2* | 5743 | prostaglandin-endoperoxide synthase 2 (prostaglandin G/H synthase and cyclooxygenase) | Luciferase reporter assay//Western blot |
| hsa-miR-26b | *CCNE1* | 898 | cyclin E1 | Luciferase reporter assay//Western blot |
| hsa-miR-26b | *CDK6* | 1021 | cyclin-dependent kinase 6 | Luciferase reporter assay//Western blot |
| hsa-miR-28 | *CDKN1A* | 1026 | cyclin-dependent kinase inhibitor 1A (p21, Cip1) | qRT-PCR//Luciferase reporter assay//Western blot |
| hsa-miR-28 | *E2F6* | 1876 | E2F transcription factor 6 | Luciferase reporter assay |
| hsa-miR-28 | *MAPK1* | 5594 | mitogen-activated protein kinase 1 | Luciferase reporter assay |
| hsa-miR-28 | *MPL* | 4352 | myeloproliferative leukemia virus oncogene | Luciferase reporter assay//qRT-PCR//Western blot |
| hsa-miR-28 | *N4BP1* | 9683 | NEDD4 binding protein 1 | Luciferase reporter assay |
| hsa-miR-28 | *OTUB1* | 55611 | OTU domain, ubiquitin aldehyde binding 1 | Luciferase reporter assay |
| hsa-miR-28 | *TEX261* | 113419 | testis expressed 261 | Luciferase reporter assay |
| hsa-miR-29b | *ADAM12* | 8038 | ADAM metallopeptidase domain 12 | Luciferase reporter assay//Microarray//Northern blot//qRT-PCR |
| hsa-miR-29b | *BACE1* | 23621 | beta-site APP-cleaving enzyme 1 | Luciferase reporter assay//Western blot |
| hsa-miR-29b | *BCL2* | 596 | B-cell CLL/lymphoma 2 | Luciferase reporter assay//qRT-PCR//Western blot |
| hsa-miR-29b | *CDK6* | 1021 | cyclin-dependent kinase 6 | qRT-PCR//Luciferase reporter assay//Western blot |
| hsa-miR-29b | *COL1A1* | 1277 | collagen, type I, alpha 1 | Immunoblot//Luciferase reporter assay//qRT-PCR |
| hsa-miR-29b | *COL3A1* | 1281 | collagen, type III, alpha 1 | Luciferase reporter assay//qRT-PCR//Western blot |
| hsa-miR-29b | *COL4A1* | 1282 | collagen, type IV, alpha 1 | Luciferase reporter assay//qRT-PCR//Western blot |
| hsa-miR-29b | *DNAJB11* | 51726 | DnaJ (Hsp40) homolog, subfamily B, member 11 | Immunoprecipitaion//Luciferase reporter assay//Western blot |
| hsa-miR-29b | *DNMT1* | 1786 | DNA (cytosine-5-)-methyltransferase 1 | immunoblot//Luciferase reporter assay//qRT-PCR |
| hsa-miR-29b | *DNMT3A* | 1788 | DNA (cytosine-5-)-methyltransferase 3 alpha | Luciferase reporter assay//Western blot//qRT-PCR |
| hsa-miR-29b | *DNMT3B* | 1789 | DNA (cytosine-5-)-methyltransferase 3 beta | Luciferase reporter assay//Western blot//qRT-PCR |
| hsa-miR-29b | *ESR1* | 2099 | estrogen receptor 1 | Western blot |
| hsa-miR-29b | *FGA* | 2243 | fibrinogen alpha chain | Luciferase reporter assay |
| hsa-miR-29b | *FGB* | 2244 | fibrinogen beta chain | Luciferase reporter assay |
| hsa-miR-29b | *FGG* | 2266 | fibrinogen gamma chain | ELISA//Flow//Luciferase reporter assay |
| hsa-miR-29b | *GRN* | 2896 | granulin | ELISA//Luciferase reporter assay//qRT-PCR//Western blot |
| hsa-miR-29b | *MCL1* | 4170 | myeloid cell leukemia sequence 1 (BCL2-related) | Luciferase reporter assay//Western blot//Immunofluorescence |
| hsa-miR-29b | *MMP15* | 4324 | matrix metallopeptidase 15 (membrane-inserted) | Luciferase reporter assay//qRT-PCR |
| hsa-miR-29b | *MMP2* | 4313 | matrix metallopeptidase 2 (gelatinase A, 72kDa gelatinase, 72kDa type IV collagenase) | Luciferase reporter assay//qRT-PCR//Western blot |
| hsa-miR-29b | *MMP24* | 10893 | matrix metallopeptidase 24 (membrane-inserted) | Luciferase reporter assay//qRT-PCR |
| hsa-miR-29b | *NCOA3* | 8202 | nuclear receptor coactivator 3 | Western blot |
| hsa-miR-29b | *NID1* | 4811 | nidogen 1 | Luciferase reporter assay//Microarray//Northern blot//qRT-PCR |
| hsa-miR-29b | *S100B* | 6285 | S100 calcium binding protein B | Luciferase reporter assay//qRT-PCR//Northern blot |
| hsa-miR-29b | *SFPQ* | 6421 | splicing factor proline/glutamine-rich | Immunoprecipitaion//Luciferase reporter assay//Western blot |
| hsa-miR-29b | *SP1* | 6667 | Sp1 transcription factor | Immunoblot//Luciferase reporter assay//qRT-PCR |
| hsa-miR-29b | *TCL1A* | 8115 | T-cell leukemia/lymphoma 1A | Luciferase reporter assay |
| hsa-miR-29b | *TET1* | 80312 | tet methylcytosine dioxygenase 1 | qRT-PCR//Luciferase reporter assay//Western blot |
| hsa-miR-29b | *VEGFA* | 7422 | vascular endothelial growth factor A | ELISA//Luciferase reporter assay |
| hsa-miR-29b | *BMP1* | 649 | bone morphogenetic protein 1 | ELISA//GFP reporter assay//qRT-PCR |
| hsa-miR-29b | *CDC42* | 998 | cell division cycle 42 | Luciferase reporter assay//Western blot |
| hsa-miR-29b | *HMGA2* | 8091 | high mobility group AT-hook 2 | Immunofluorescence//Immunohistochemistry//Luciferase reporter assay//qRT-PCR//Western blot |
| hsa-miR-29b | *PPP1R13B* | 23368 | protein phosphatase 1, regulatory subunit 13B | Luciferase reporter assay//Western blot |
| hsa-miR-29b | *PTEN* | 5728 | phosphatase and tensin homolog | Immunoblot//Immunofluorescence//qRT-PCR//Western blot |
| hsa-miR-29b | *TGFB1* | 7040 | transforming growth factor, beta 1 | ELISA//GFP reporter assay//qRT-PCR |
| hsa-miR-29b | *TGFB2* | 7042 | transforming growth factor, beta 2 | ELISA//GFP reporter assay//qRT-PCR |
| hsa-miR-30a | *ADPGK* | 83440 | ADP-dependent glucokinase | pSILAC |
| hsa-miR-30a | *ANPEP* | 290 | alanyl (membrane) aminopeptidase | pSILAC |
| hsa-miR-30a | *AP2A1* | 160 | adaptor-related protein complex 2, alpha 1 subunit | pSILAC |
| hsa-miR-30a | *ATP2A2* | 488 | ATPase, Ca++ transporting, cardiac muscle, slow twitch 2 | pSILAC |
| hsa-miR-30a | *ATRX* | 546 | alpha thalassemia/mental retardation syndrome X-linked | pSILAC |
| hsa-miR-30a | *BDNF* | 627 | brain-derived neurotrophic factor | Luciferase reporter assay |
| hsa-miR-30a | *BECN1* | 8678 | beclin 1, autophagy related | Luciferase reporter assay |
| hsa-miR-30a | *C1D* | 10438 | C1D nuclear receptor corepressor | pSILAC |
| hsa-miR-30a | *C5orf15* | 56951 | chromosome 5 open reading frame 15 | pSILAC |
| hsa-miR-30a | *CBFB* | 865 | core-binding factor, beta subunit | pSILAC |
| hsa-miR-30a | *CDCP1* | 64866 | CUB domain containing protein 1 | pSILAC |
| hsa-miR-30a | *CEP72* | 55722 | centrosomal protein 72kDa | pSILAC |
| hsa-miR-30a | *CHD1* | 1105 | chromodomain helicase DNA binding protein 1 | pSILAC |
| hsa-miR-30a | *CNPY4* | 245812 | canopy 4 homolog (zebrafish) | pSILAC |
| hsa-miR-30a | *CPNE8* | 144402 | copine VIII | pSILAC |
| hsa-miR-30a | *CTSL1* | 1514 | cathepsin L1 | pSILAC |
| hsa-miR-30a | *DOCK7* | 85440 | dedicator of cytokinesis 7 | pSILAC |
| hsa-miR-30a | *DPY19L1* | 23333 | dpy-19-like 1 (C. elegans) | pSILAC |
| hsa-miR-30a | *ELMOD2* | 255520 | ELMO/CED-12 domain containing 2 | pSILAC |
| hsa-miR-30a | *FRG1* | 2483 | FSHD region gene 1 | pSILAC |
| hsa-miR-30a | *FXR2* | 9513 | fragile X mental retardation, autosomal homolog 2 | pSILAC |
| hsa-miR-30a | *G3BP1* | 10146 | GTPase activating protein (SH3 domain) binding protein 1 | pSILAC |
| hsa-miR-30a | *GALNT1* | 2589 | UDP-N-acetyl-alpha-D-galactosamine:polypeptide N-acetylgalactosaminyltransferase 1 (GalNAc-T1) | pSILAC |
| hsa-miR-30a | *GALNT7* | 51809 | UDP-N-acetyl-alpha-D-galactosamine:polypeptide N-acetylgalactosaminyltransferase 7 (GalNAc-T7) | pSILAC |
| hsa-miR-30a | *GNAI2* | 2771 | guanine nucleotide binding protein (G protein), alpha inhibiting activity polypeptide 2 | pSILAC |
| hsa-miR-30a | *GPD2* | 2820 | glycerol-3-phosphate dehydrogenase 2 (mitochondrial) | pSILAC |
| hsa-miR-30a | *HNRNPM* | 4670 | heterogeneous nuclear ribonucleoprotein M | pSILAC |
| hsa-miR-30a | *IDH1* | 3417 | isocitrate dehydrogenase 1 (NADP+), soluble | pSILAC |
| hsa-miR-30a | *IFRD1* | 3475 | interferon-related developmental regulator 1 | pSILAC |
| hsa-miR-30a | *ITGA2* | 3673 | integrin, alpha 2 (CD49B, alpha 2 subunit of VLA-2 receptor) | pSILAC |
| hsa-miR-30a | *JUN* | 3725 | jun proto-oncogene | pSILAC |
| hsa-miR-30a | *KDELC2* | 143888 | KDEL (Lys-Asp-Glu-Leu) containing 2 | pSILAC |
| hsa-miR-30a | *KRT85* | 3891 | keratin 85 | pSILAC |
| hsa-miR-30a | *LIMCH1* | 22998 | LIM and calponin homology domains 1 | pSILAC |
| hsa-miR-30a | *LMNB2* | 84823 | lamin B2 | pSILAC |
| hsa-miR-30a | *LRRC8C* | 84230 | leucine rich repeat containing 8 family, member C | pSILAC |
| hsa-miR-30a | *LRRFIP1* | 9208 | leucine rich repeat (in FLII) interacting protein 1 | pSILAC |
| hsa-miR-30a | *LTN1* | 26046 | listerin E3 ubiquitin protein ligase 1 | pSILAC |
| hsa-miR-30a | *MAT2A* | 4144 | methionine adenosyltransferase II, alpha | pSILAC |
| hsa-miR-30a | *MBNL1* | 4154 | muscleblind-like splicing regulator 1 | pSILAC |
| hsa-miR-30a | *MET* | 4233 | met proto-oncogene (hepatocyte growth factor receptor) | pSILAC |
| hsa-miR-30a | *MLLT11* | 10962 | myeloid/lymphoid or mixed-lineage leukemia (trithorax homolog, Drosophila); translocated to, 11 | pSILAC |
| hsa-miR-30a | *MPDU1* | 9526 | myeloid/lymphoid or mixed-lineage leukemia (trithorax homolog, Drosophila); translocated to, 11 | pSILAC |
| hsa-miR-30a | *MTX3* | 345778 | metaxin 3 | pSILAC |
| hsa-miR-30a | *MYO10* | 4651 | myosin X | pSILAC |
| hsa-miR-30a | *NAPG* | 8774 | N-ethylmaleimide-sensitive factor attachment protein, gamma | pSILAC |
| hsa-miR-30a | *NCEH1* | 57552 | neutral cholesterol ester hydrolase 1 | pSILAC |
| hsa-miR-30a | *NCL* | 4691 | nucleolin | pSILAC |
| hsa-miR-30a | *NOTCH1* | 4851 | notch 1 | Luciferase reporter assay//Microarray |
| hsa-miR-30a | *NPR3* | 4883 | natriuretic peptide receptor C/guanylate cyclase C (atrionatriuretic peptide receptor C) | pSILAC |
| hsa-miR-30a | *NT5C3* | 51251 | 5'-nucleotidase, cytosolic IIIA | pSILAC |
| hsa-miR-30a | *NT5E* | 4907 | 5'-nucleotidase, ecto (CD73) | pSILAC |
| hsa-miR-30a | *NUCB1* | 4924 | nucleobindin 1 | pSILAC |
| hsa-miR-30a | *NUFIP2* | 57532 | nuclear fragile X mental retardation protein interacting protein 2 | pSILAC |
| hsa-miR-30a | *P4HA2* | 8974 | prolyl 4-hydroxylase, alpha polypeptide II | pSILAC |
| hsa-miR-30a | *PAFAH1B2* | 5049 | platelet-activating factor acetylhydrolase 1b, catalytic subunit 2 (30kDa) | pSILAC |
| hsa-miR-30a | *PBRM1* | 55193 | polybromo 1 | pSILAC |
| hsa-miR-30a | *PEX11B* | 8799 | peroxisomal biogenesis factor 11 beta | pSILAC |
| hsa-miR-30a | *PGM1* | 5236 | phosphoglucomutase 1 | pSILAC |
| hsa-miR-30a | *PPP2R4* | 5524 | protein phosphatase 2A activator, regulatory subunit 4 | pSILAC |
| hsa-miR-30a | *PPP3CA* | 5530 | protein phosphatase 3, catalytic subunit, alpha isozyme | pSILAC |
| hsa-miR-30a | *PPP3R1* | 5534 | protein phosphatase 3, regulatory subunit B, alpha | pSILAC |
| hsa-miR-30a | *PRPF40A* | 55660 | PRP40 pre-mRNA processing factor 40 homolog A (S. cerevisiae) | pSILAC |
| hsa-miR-30a | *PTGFRN* | 5738 | prostaglandin F2 receptor inhibitor | pSILAC |
| hsa-miR-30a | *PTPRK* | 5796 | protein tyrosine phosphatase, receptor type, K | pSILAC |
| hsa-miR-30a | *PTRH1* | 138428 | peptidyl-tRNA hydrolase 1 homolog (S. cerevisiae) | pSILAC |
| hsa-miR-30a | *RAB27B* | 5874 | RAB27B, member RAS oncogene family | pSILAC |
| hsa-miR-30a | *RAD23B* | 5887 | RAD23 homolog B (S. cerevisiae) | pSILAC |
| hsa-miR-30a | *RBMS1* | 5937 | RNA binding motif, single stranded interacting protein 1 | pSILAC |
| hsa-miR-30a | *RQCD1* | 9125 | RCD1 required for cell differentiation1 homolog (S. pombe) | pSILAC |
| hsa-miR-30a | *RRP36* | 88745 | ribosomal RNA processing 36 homolog (S. cerevisiae) | pSILAC |
| hsa-miR-30a | *SEC11C* | 90701 | SEC11 homolog C (S. cerevisiae) | pSILAC |
| hsa-miR-30a | *SEC23A* | 10484 | Sec23 homolog A (S. cerevisiae) | pSILAC |
| hsa-miR-30a | *SEC62* | 7095 | SEC62 homolog (S. cerevisiae) | pSILAC |
| hsa-miR-30a | *SLC12A4* | 6560 | solute carrier family 12 (potassium/chloride transporters), member 4 | pSILAC |
| hsa-miR-30a | *SLC38A1* | 81539 | solute carrier family 38, member 1 | pSILAC |
| hsa-miR-30a | *SLC38A2* | 54407 | solute carrier family 38, member 2 | pSILAC |
| hsa-miR-30a | *SLC4A10* | 57282 | solute carrier family 4, sodium bicarbonate transporter, member 10 | pSILAC |
| hsa-miR-30a | *SLC4A7* | 9497 | solute carrier family 4, sodium bicarbonate cotransporter, member 7 | pSILAC |
| hsa-miR-30a | *SLC7A11* | 23657 | solute carrier family 4, sodium borate transporter, member 11 | pSILAC |
| hsa-miR-30a | *SLC9A3R2* | 9351 | solute carrier family 9, subfamily A (NHE3, cation proton antiporter 3), member 3 regulator 2 | pSILAC |
| hsa-miR-30a | *STMN1* | 3925 | stathmin 1 | pSILAC |
| hsa-miR-30a | *STRN* | 6801 | striatin, calmodulin binding protein | pSILAC |
| hsa-miR-30a | *STX7* | 8417 | syntaxin 7 | pSILAC |
| hsa-miR-30a | *SYPL1* | 6856 | synaptophysin-like 1 | pSILAC |
| hsa-miR-30a | *TBCB* | 1155 | tubulin folding cofactor B | pSILAC |
| hsa-miR-30a | *THEM4* | 117145 | thioesterase superfamily member 4 | pSILAC |
| hsa-miR-30a | *TMCO1* | 54499 | transmembrane and coiled-coil domains 1 | pSILAC |
| hsa-miR-30a | *TMED10* | 10972 | transmembrane emp24-like trafficking protein 10 (yeast) | pSILAC |
| hsa-miR-30a | *TMED2* | 10959 | transmembrane emp24 domain trafficking protein 2 | pSILAC |
| hsa-miR-30a | *TMED3* | 23423 | transmembrane emp24 protein transport domain containing 3 | pSILAC |
| hsa-miR-30a | *TMED7* | 51014 | transmembrane emp24 protein transport domain containing 7 | pSILAC |
| hsa-miR-30a | *TMEM41B* | 440026 | transmembrane protein 41B | pSILAC |
| hsa-miR-30a | *TMEM59* | 9528 | transmembrane protein 59 | pSILAC |
| hsa-miR-30a | *TMEM87A* | 25963 | transmembrane protein 87A | pSILAC |
| hsa-miR-30a | *TNFAIP2* | 7127 | tumor necrosis factor, alpha-induced protein 2 | pSILAC |
| hsa-miR-30a | *TNFRSF10B* | 8795 | tumor necrosis factor receptor superfamily, member 10b | pSILAC |
| hsa-miR-30a | *TNRC6A* | 27327 | trinucleotide repeat containing 6A | Luciferase reporter assay |
| hsa-miR-30a | *TWF1* | 5756 | twinfilin actin-binding protein 1 | pSILAC |
| hsa-miR-30a | *UAP1* | 6675 | UDP-N-acteylglucosamine pyrophosphorylase 1 | pSILAC |
| hsa-miR-30a | *WDR82* | 80335 | WD repeat domain 82 | pSILAC |
| hsa-miR-30a | *WNT5A* | 7474 | wingless-type MMTV integration site family, member 5A | pSILAC |
| hsa-miR-30a | *DTL* | 51514 | denticleless E3 ubiquitin protein ligase homolog (Drosophila) | Flow//Luciferase reporter assay//qRT-PCR//Western blot |
| hsa-miR-30a | *SMAD1* | 4086 | SMAD family member 1 | Luciferase reporter assay//Western blot |
| hsa-miR-30b | *BCL6* | 604 | B-cell CLL/lymphoma 6 | Luciferase reporter assay//qRT-PCR//Western blot |
| hsa-miR-30b | *CCNE2* | 9134 | cyclin E2 | Luciferase reporter assay//qRT-PCR |
| hsa-miR-30b | *SMAD1* | 4086 | SMAD family member 1 | Luciferase reporter assay//Western blot |
| hsa-miR-30b | *SOCS1* | 8651 | suppressor of cytokine signaling 1 | Luciferase reporter assay//qRT-PCR//Western blot |
| hsa-miR-320 | *AQP1* | 358 | aquaporin 1 (Colton blood group) | Luciferase reporter assay |
| hsa-miR-320 | *AQP4* | 361 | aquaporin 4 | Luciferase reporter assay |
| hsa-miR-320 | *HSPB6* | 126393 | heat shock protein, alpha-crystallin-related, B6 | Luciferase reporter assay |
| hsa-miR-320 | *MCL1* | 4170 | myeloid cell leukemia sequence 1 (BCL2-related) | Luciferase reporter assay |
| hsa-miR-320 | *NPR1* | 4881 | natriuretic peptide receptor A/guanylate cyclase A (atrionatriuretic peptide receptor A) | Luciferase reporter assay//Western blot |
| hsa-miR-320 | *POLR3D* | 661 | polymerase (RNA) III (DNA directed) polypeptide D, 44kDa | TaqMan miRNA assay/RT-PCR |
| hsa-miR-320 | *TAC1* | 6863 | tachykinin, precursor 1 | Luciferase reporter assay |
| hsa-miR-320 | *TFRC* | 7037 | transferrin receptor (p90, CD71) | Luciferase reporter assay |
| hsa-miR-338 | *DAB2IP* | 153090 | DAB2 interacting protein | qRT-PCR//Western blot |
| hsa-miR-338 | *MAP1A* | 4130 | microtubule-associated protein 1A | qRT-PCR//Western blot |
| hsa-miR-338 | *NOVA1* | 4857 | neuro-oncological ventral antigen 1 | qRT-PCR//Western blot |
| hsa-miR-338 | *PLA2G4B* | 100137049 | phospholipase A2, group IVB (cytosolic) | Immunoblot//Immunocytochemistry//Luciferase reporter assay//Microarray//qRT-PCR |
| hsa-miR-338 | *SMO* | 6608 | smoothened, frizzled family receptor | Luciferase reporter assay//qRT-PCR//Western blot |
| hsa-miR-338 | *UBE2Q1* | 55585 | ubiquitin-conjugating enzyme E2Q family member 1 | qRT-PCR//Western blot |
| hsa-miR-338 | *ZNF238* | 10472 | zinc finger and BTB domain containing 18 | qRT-PCR//Western blot |
| hsa-miR-339 | *BCL6* | 604 | B-cell CLL/lymphoma 6 | qRT-PCR |
| hsa-miR-33a | *ABCA1* | 19 | ATP-binding cassette, sub-family A (ABC1), member 1 | Luciferase reporter assay |
| hsa-miR-33a | *NPC1* | 4864 | Niemann-Pick disease, type C1 | Luciferase reporter assay |
| hsa-miR-345 | *ABCC1* | 4363 | ATP-binding cassette, sub-family C (CFTR/MRP), member 1 | Luciferase reporter assay//Western blot |
| hsa-miR-345 | *CDKN1A* | 1026 | cyclin-dependent kinase inhibitor 1A (p21, Cip1) | qRT-PCR//Luciferase reporter assay//Western blot |
| hsa-miR-345 | *NTRK3* | 4916 | neurotrophic tyrosine kinase, receptor, type 3 | Luciferase reporter assay |
| hsa-miR-34a | *AXIN2* | 8313 | axin 2 | Luciferase reporter assay |
| hsa-miR-34a | *BCL2* | 596 | B-cell CLL/lymphoma 2 | Luciferase reporter assay |
| hsa-miR-34a | *BIRC3* | 330 | baculoviral IAP repeat containing 3 | Microarray//Northern blot |
| hsa-miR-34a | *CCND1* | 595 | cyclin D1 | qRT-PCR//Luciferase reporter assay//Western blot |
| hsa-miR-34a | *CCND3* | 896 | cyclin D3 | Western blot |
| hsa-miR-34a | *CCNE2* | 9134 | cyclin E2 | Luciferase reporter assay |
| hsa-miR-34a | *CD44* | 960 | CD44 molecule (Indian blood group) | Immunohistochemistry//qRT-PCR//Western blot |
| hsa-miR-34a | *CDC25A* | 993 | cell division cycle 25A | Western blot |
| hsa-miR-34a | *CDC25C* | 995 | cell division cycle 25C | Microarray |
| hsa-miR-34a | *CDK4* | 1019 | cyclin-dependent kinase 4 | Luciferase reporter assay |
| hsa-miR-34a | *CDK6* | 1021 | cyclin-dependent kinase 6 | qRT-PCR//Luciferase reporter assay//Western blot |
| hsa-miR-34a | *CEBPB* | 1051 | CCAAT/enhancer binding protein (C/EBP), beta | Luciferase reporter assay |
| hsa-miR-34a | *DLL1* | 28514 | delta-like 1 (Drosophila) | Luciferase reporter assay |
| hsa-miR-34a | *E2F1* | 1869 | E2F transcription factor 1 | Immunoblot//Immunohistochemistry//qRT-PCR |
| hsa-miR-34a | *E2F3* | 1871 | E2F transcription factor 3 | Luciferase reporter assay//Western blot |
| hsa-miR-34a | *E2F5* | 1875 | E2F transcription factor 5, p130-binding | Microarray |
| hsa-miR-34a | *EMP1* | 2012 | epithelial membrane protein 1 | Microarray//Northern blot |
| hsa-miR-34a | *FOXP1* | 27086 | forkhead box P1 | Flow//Immunoblot//Luciferase reporter assay |
| hsa-miR-34a | *GRM7* | 2917 | glutamate receptor, metabotropic 7 | qRT-PCR |
| hsa-miR-34a | *HMGA2* | 8091 | high mobility group AT-hook 2 | Microarray |
| hsa-miR-34a | *HNF4A* | 3172 | hepatocyte nuclear factor 4, alpha | Luciferase reporter assay//qRT-PCR//Western blot |
| hsa-miR-34a | *IFNB1* | 3456 | interferon, beta 1, fibroblast | ELISA//Luciferase reporter assay//qRT-PCR |
| hsa-miR-34a | *JAG1* | 182 | jagged 1 | Luciferase reporter assay//qRT-PCR//Western blot |
| hsa-miR-34a | *MAGEA12* | 4111 | melanoma antigen family A, 12 | Immunohistochemistry//Luciferase reporter assay//qRT-PCR//Western blot |
| hsa-miR-34a | *MAGEA2* | 4101 | melanoma antigen family A, 2 | Immunocytochemistry//Luciferase reporter assay//qRT-PCR//Western blot |
| hsa-miR-34a | *MAGEA3* | 4102 | melanoma antigen family A, 3 | Immunocytochemistry//Luciferase reporter assay//qRT-PCR//Western blot |
| hsa-miR-34a | *MAGEA6* | 4105 | melanoma antigen family A, 6 | Immunocytochemistry//Luciferase reporter assay//qRT-PCR//Western blot |
| hsa-miR-34a | *MAP2K1* | 5604 | mitogen-activated protein kinase kinase 1 | qRT-PCR//Luciferase reporter assay//Western blot//Northern blot |
| hsa-miR-34a | *MAP3K9* | 4293 | mitogen-activated protein kinase kinase kinase 9 | Luciferase reporter assay//qRT-PCR//Western blot |
| hsa-miR-34a | *MDM4* | 4194 | Mdm4 p53 binding protein homolog (mouse) | Microarray |
| hsa-miR-34a | *MET* | 4233 | met proto-oncogene (hepatocyte growth factor receptor) | Luciferase reporter assay |
| hsa-miR-34a | *MYB* | 4602 | v-myb myeloblastosis viral oncogene homolog (avian) | qRT-PCR//Western blot//Luciferase reporter assay |
| hsa-miR-34a | *MYC* | 4609 | v-myc myelocytomatosis viral oncogene homolog (avian) | Luciferase reporter assay//qRT-PCR//Western blot |
| hsa-miR-34a | *MYCN* | 4613 | v-myc myelocytomatosis viral related oncogene, neuroblastoma derived (avian) | Luciferase reporter assay//Western blot |
| hsa-miR-34a | *NOTCH1* | 4851 | notch 1 | Immunofluorescence//Immunohistochemistry//Luciferase reporter assay//Microarray//qRT-PCR//Western blot |
| hsa-miR-34a | *NOTCH2* | 4853 | notch 2 | Immunoblot//Immunocytochemistry//Luciferase reporter assay//Microarray//qRT-PCR//Western blot |
| hsa-miR-34a | *PEA15* | 8682 | phosphoprotein enriched in astrocytes 15 | Western blot |
| hsa-miR-34a | *SIRT1* | 23411 | sirtuin 1 | qRT-PCR//Western blot |
| hsa-miR-34a | *SPI1* | 6688 | spleen focus forming virus (SFFV) proviral integration oncogene spi1 | Luciferase reporter assay |
| hsa-miR-34a | *TNFRSF6B* | 8771 | tumor necrosis factor receptor superfamily, member 6b, decoy | Microarray//Northern blot |
| hsa-miR-34a | *VAMP2* | 6844 | vesicle-associated membrane protein 2 (synaptobrevin 2) | Luciferase reporter assay |
| hsa-miR-34a | *VEGFA* | 7422 | vascular endothelial growth factor A | ELISA//Luciferase reporter assay |
| hsa-miR-34a | *WNT1* | 7471 | wingless-type MMTV integration site family, member 1 | Luciferase reporter assay |
| hsa-miR-34a | *YY1* | 7528 | YY1 transcription factor | Luciferase reporter assay//Western blot |
| hsa-miR-34a | *ZAP70* | 7535 | zeta-chain (TCR) associated protein kinase 70kDa | Immunoblot//Luciferase reporter assay |
| hsa-miR-34a | *EPHA5* | 2044 | EPH receptor A5 | Luciferase reporter assay//Western blot |
| hsa-miR-34a | *FOSL1* | 8061 | FOS-like antigen 1 | Immunoprecipitaion//Luciferase reporter assay//qRT-PCR//Western blot |
| hsa-miR-34a | *IMPA1* | 3612 | inositol(myo)-1(or 4)-monophosphatase 1 | Luciferase reporter assay |
| hsa-miR-34a | *IMPDH2* | 3615 | IMP (inosine 5'-monophosphate) dehydrogenase 2 | Luciferase reporter assay |
| hsa-miR-34a | *MET* | 4233 | met proto-oncogene (hepatocyte growth factor receptor) | Luciferase reporter assay//qRT-PCR//Western blot |
| hsa-miR-34a | *NANOG* | 79923 | Nanog homeobox | Luciferase reporter assay//Western blot |
| hsa-miR-34a | *PDGFRA* | 5156 | platelet-derived growth factor receptor, alpha polypeptide | Immunoblot//Luciferase reporter assay//Microarray//qRT-PCR |
| hsa-miR-34a | *SOX2* | 6657 | SRY (sex determining region Y)-box 2 | Luciferase reporter assay//Western blot |
| hsa-miR-34a | *STX1A* | 6804 | syntaxin 1A (brain) | Luciferase reporter assay//Western blot |
| hsa-miR-34a | *SYT1* | 6857 | synaptotagmin I | Luciferase reporter assay//Western blot |
| hsa-miR-34a | *ULBP2* | 80328 | UL16 binding protein 2 | Flow//Luciferase reporter assay//Western blot |
| hsa-miR-429 | *EP300* | 2033 | E1A binding protein p300 | Microarray |
| hsa-miR-429 | *RERE* | 473 | arginine-glutamic acid dipeptide (RE) repeats | Luciferase reporter assay |
| hsa-miR-429 | *SIP1* | 8487 | gem (nuclear organelle) associated protein 2 | Luciferase reporter assay |
| hsa-miR-429 | *WASF3* | 10810 | WAS protein family, member 3 | Luciferase reporter assay |
| hsa-miR-429 | *ZEB1* | 6935 | zinc finger E-box binding homeobox 1 | Luciferase reporter assay//qRT-PCR |
| hsa-miR-429 | *ZEB2* | 9839 | zinc finger E-box binding homeobox 2 | Luciferase reporter assay//qRT-PCR |
| hsa-miR-429 | *ZFPM2* | 23414 | zinc finger protein, FOG family member 2 | Luciferase reporter assay//Western blot |
| hsa-miR-451 | *ABCB1* | 5243 | ATP-binding cassette, sub-family B (MDR/TAP), member 1 | qRT-PCR//Luciferase reporter assay//Western blot |
| hsa-miR-451 | *AKT1* | 207 | v-akt murine thymoma viral oncogene homolog 1 | qRT-PCR//Western blot |
| hsa-miR-451 | *BCL2* | 596 | B-cell CLL/lymphoma 2 | qRT-PCR//Western blot |
| hsa-miR-451 | *CAB39* | 51719 | calcium binding protein 39 | qRT-PCR//Luciferase reporter assay//Western blot//Microarray |
| hsa-miR-451 | *MIF* | 4282 | macrophage migration inhibitory factor (glycosylation-inhibiting factor) | ELISA//Luciferase reporter assay//Microarray//qRT-PCR//Western blot |
| hsa-miR-451 | *MMP2* | 4313 | matrix metallopeptidase 2 (gelatinase A, 72kDa gelatinase, 72kDa type IV collagenase) | qRT-PCR//Western blot |
| hsa-miR-451 | *MMP9* | 4318 | matrix metallopeptidase 9 (gelatinase B, 92kDa gelatinase, 92kDa type IV collagenase) | qRT-PCR//Western blot |
| hsa-miR-451 | *SSSCA1* | 10534 | Sjogren syndrome/scleroderma autoantigen 1 | qRT-PCR//Western blot |
| hsa-miR-451 | *MYC* | 4609 | v-myc myelocytomatosis viral oncogene homolog (avian) | Luciferase reporter assay |
| hsa-miR-451 | *RAB14* | 51552 | RAB14, member RAS oncogene family | Flow//Luciferase reporter assay//Microarray//qRT-PCR//Western blot |
| hsa-miR-486-5p | *CD40* | 958 | CD40 molecule, TNF receptor superfamily member 5 | Microarray//qRT-PCR |
| hsa-miR-503 | *ANLN* | 54443 | anillin, actin binding protein | Luciferase reporter assay |
| hsa-miR-503 | *ATF6* | 22926 | activating transcription factor 6 | Luciferase reporter assay |
| hsa-miR-503 | *CCND1* | 595 | cyclin D1 | Luciferase reporter assay//qRT-PCR//Western blot |
| hsa-miR-503 | *CCNE1* | 898 | cyclin E1 | Luciferase reporter assay |
| hsa-miR-503 | *CCNE2* | 9134 | cyclin E2 | Luciferase reporter assay |
| hsa-miR-503 | *CCNF* | 899 | cyclin F | Luciferase reporter assay |
| hsa-miR-503 | *CDC14A* | 8556 | cell division cycle 14A | Luciferase reporter assay |
| hsa-miR-503 | *CDC25A* | 993 | cell division cycle 25A | Luciferase reporter assay |
| hsa-miR-503 | *CDKN1A* | 1026 | cyclin-dependent kinase inhibitor 1A (p21, Cip1) | Luciferase reporter assay |
| hsa-miR-503 | *CHEK1* | 1111 | checkpoint kinase 1 | Luciferase reporter assay |
| hsa-miR-503 | *EIF2C1* | 26523 | argonaute RISC catalytic component 1 | Luciferase reporter assay |
| hsa-miR-503 | *WEE1* | 7465 | WEE1 homolog (S. pombe) | Luciferase reporter assay |
| hsa-miR-545 | *LRP1* | 4035 | low density lipoprotein receptor-related protein 1 | qRT-PCR//Luciferase reporter assay//Western blot |
| hsa-miR-626 | *SLC7A5* | 8140 | solute carrier family 7 (amino acid transporter light chain, L system), member 5 | Immunofluorescence//Luciferase reporter assay//qRT-PCR//Western blot//Immunoprecipitation |
| hsa-miR-96 | *ADCY6* | 112 | adenylate cyclase 6 | Luciferase reporter assay |
| hsa-miR-96 | *CDKN1A* | 1026 | cyclin-dependent kinase inhibitor 1A (p21, Cip1) | Luciferase reporter assay//Microarray//qRT-PCR//Western blot |
| hsa-miR-96 | *FOXO1* | 2308 | forkhead box O1 | qRT-PCR//Luciferase reporter assay//Western blot |
| hsa-miR-96 | *FOXO3* | 2309 | forkhead box O3 | Luciferase reporter assay//qRT-PCR//Western blot |
| hsa-miR-96 | *HTR1B* | 3351 | 5-hydroxytryptamine (serotonin) receptor 1B, G protein-coupled | Reporter assay |
| hsa-miR-96 | *KRAS* | 3845 | v-Ki-ras2 Kirsten rat sarcoma viral oncogene homolog | Immunoblot//Immunohistochemistry//Northern blot//Quantitative proteomic approach//Western blot |
| hsa-miR-96 | *MITF* | 4286 | microphthalmia-associated transcription factor | Luciferase reporter assay |
| hsa-miR-96 | *PRMT5* | 10419 | protein arginine methyltransferase 5 | Western blot |

List of experimentally validated targets from miRTarBase for miRNAs upregulated and downregulated in gastric cancer. Total 912 redundant target genes with 701 non-redundant target genes are listed with MicroRNA, Target gene, Entrez gene ID, Gene name and Validation method. Abbreviation: ChiP: chromatin immunoprecipitation, ELISA: enzyme-linked immunosorbent assay, FACS: fluorescence-activated cell sorting, GFP: green fluorescent protein, pSILAC: pulsed stable isotope labeling with amino acids in culture, qRT-PCR: quantitative RT-PCR

**Supplementary Table S5.** Gene ontology terms of target genes for down-regulated miRNAs in gastric cancer

| **GO term** | **Count** | **p-value** |
| --- | --- | --- |
| GO:0042127- regulation of cell proliferation | 57 | 1.34E-13 |
| GO:0042981- regulation of apoptosis | 57 | 3.27E-13 |
| GO:0043067- regulation of programmed cell death | 57 | 4.92E-13 |
| GO:0010941- regulation of cell death | 57 | 5.71E-13 |
| GO:0010033- response to organic substance | 53 | 6.83E-13 |
| GO:0007242- intracellular signaling cascade | 71 | 1.10E-11 |
| GO:0051726- regulation of cell cycle | 32 | 8.19E-11 |
| GO:0010604- positive regulation of macromolecule metabolic process | 53 | 4.28E-10 |
| GO:0043066- negative regulation of apoptosis | 32 | 4.39E-10 |
| GO:0043069- negative regulation of programmed cell death | 32 | 6.18E-10 |
| GO:0060548- negative regulation of cell death | 32 | 6.65E-10 |
| GO:0007167- enzyme linked receptor protein signaling pathway | 31 | 8.17E-10 |
| GO:0043065- positive regulation of apoptosis | 35 | 9.02E-10 |
| GO:0043068- positive regulation of programmed cell death | 35 | 1.08E-09 |
| GO:0010942- positive regulation of cell death | 35 | 1.22E-09 |
| GO:0031328- positive regulation of cellular biosynthetic process | 45 | 2.01E-09 |
| GO:0051173- positive regulation of nitrogen compound metabolic process | 43 | 3.11E-09 |
| GO:0009891- positive regulation of biosynthetic process | 45 | 3.13E-09 |
| GO:0001775- cell activation | 27 | 5.89E-09 |
| GO:0045597- positive regulation of cell differentiation | 24 | 6.53E-09 |

**Supplementary Table S6**. KEGG pathways of target genes for down-regulated miRNAs in gastric cancer

| **KEGG Pathway** | **Count** | **p-value** |
| --- | --- | --- |
| hsa05200: Pathways in cancer | 47 | 3.81E-18 |
| hsa05210: Colorectal cancer | 19 | 1.41E-10 |
| hsa05222: Small cell lung cancer | 16 | 7.21E-08 |
| hsa05212: Pancreatic cancer | 13 | 3.33E-06 |
| hsa04722: Neutrophin signaling pathway | 16 | 1.22E-05 |
| hsa04210: Apoptosis | 13 | 2.49E-05 |
| hsa05220: Chronic myeloid leukemia | 12 | 3.05E-05 |
| hsa04510: Focal adhesion | 20 | 3.07E-05 |
| hsa05215: Prostate cancer | 13 | 3.15E-05 |
| hsa05213: Endometrial cancer | 10 | 4.30E-05 |
| hsa04660: T cell receptor signaling pathway | 14 | 5.01E-05 |
| hsa05219: Bladder cancer | 9 | 5.67E-05 |
| hsa05218: Melanoma | 11 | 1.01E-04 |
| hsa04620: Toll-like receptor signaling pathway | 13 | 1.12E-04 |
| hsa04010: MAPK signaling pathway | 22 | 1.74E-04 |
| hsa05120: Epithelial cell signaling in Helicobacter pylori infection | 10 | 3.65E-04 |
| hsa04520: Adherens junction | 10 | 9.24E-04 |
| hsa05214: Glioma | 9 | 1.02E-03 |
| hsa04115: p53 signaling pathway | 9 | 1.70E-03 |
| hsa05223: Non-small cell lung cancer | 8 | 1.87E-03 |

**Supplementary Table S7**. The number of microRNAs mentioned in two references with inconsistent direction (study-study inconsistent miRNA matrix)

| **Study No^*^.** | 14 | 15 | 16 | 17 | 18 | 19 | 20 | 21 | 22 | 23 | 24 | 25 | 26 | 27 |
| --- | --- | --- | --- | --- | --- | --- | --- | --- | --- | --- | --- | --- | --- | --- |
| 14 |  | 1 | 2 | 0 | 1 | 0 | 0 | 0 | 0 | 0 | 0 | 0 | 0 | 0 |
| 15 | 1 |  | 3 | 2 | 13 | 4 | 0 | 2 | 1 | 7 | 6 | 3 | 2 | 1 |
| 16 | 2 | 3 |  | 1 | 4 | 4 | 1 | 3 | 1 | 5 | 2 | 1 | 2 | 3 |
| 17 | 0 | 2 | 1 |  | 2 | 4 | 0 | 0 | 1 | 2 | 1 | 1 | 1 | 0 |
| 18 | 1 | 13 | 4 | 2 |  | 1 | 0 | 1 | 4 | 5 | 1 | 0 | 0 | 1 |
| 19 | 0 | 4 | 4 | 4 | 1 |  | 0 | 0 | 1 | 3 | 0 | 0 | 0 | 0 |
| 20 | 0 | 0 | 1 | 0 | 0 | 0 |  | 0 | 0 | 0 | 0 | 0 | 0 | 0 |
| 21 | 0 | 2 | 3 | 0 | 1 | 0 | 0 |  | 0 | 4 | 1 | 0 | 0 | 0 |
| 22 | 0 | 1 | 1 | 1 | 4 | 1 | 0 | 0 |  | 0 | 2 | 2 | 0 | 0 |
| 23 | 0 | 7 | 5 | 2 | 5 | 3 | 0 | 4 | 0 |  | 2 | 2 | 2 | 4 |
| 24 | 0 | 6 | 2 | 1 | 1 | 0 | 0 | 1 | 2 | 2 |  | 0 | 1 | 0 |
| 25 | 0 | 3 | 1 | 1 | 0 | 0 | 0 | 0 | 2 | 2 | 0 |  | 1 | 0 |
| 26 | 0 | 2 | 2 | 1 | 0 | 0 | 0 | 0 | 0 | 2 | 1 | 1 |  | 0 |
| 27 | 0 | 1 | 3 | 0 | 1 | 0 | 0 | 0 | 0 | 4 | 0 | 0 | 0 |  |
| Sum | 4 | 45 | 32 | 15 | 33 | 17 | 1 | 11 | 12 | 36 | 16 | 10 | 9 | 9 |

* The study number is the same as the reference number shown in the main manuscript
